# Characterization of blood serum and cell lines by an automated high-throughput well plate sampling system using laser ablation-rapid evaporative ionization mass spectrometry

**DOI:** 10.1007/s00216-026-06484-4

**Published:** 2026-04-28

**Authors:** Gabriel S. Horkovics-Kováts, Daniel Simon, Richárd Schäffer, István Pap, Zahra Nozari, Alexander König, Sonja Decking-Paede, Roland Szand, Adrienn Molnár, Gitta Schlosser, Nóra Kucsma, Gergely Szakács, Zoltán Takáts, Júlia Balog

**Affiliations:** 1 Hevesy György PhD School of Chemistry, ELTE Eötvös Loránd University, 1117 Budapest, Hungary; 2 Waters Research Center, 1031 Budapest, Hungary; 3 MTA-ELTE Lendület (Momentum) Ion Mobility Mass Spectrometry Research Group, Institute of Chemistry, ELTE Eötvös Loránd University, 1117 Budapest, Hungary; 4 Department of Immunomedicine, University of Regensburg, 93053 Regensburg, Germany; 5 Department of Otorhinolaryngology, University Hospital Regensburg, 93053 Regensburg, Germany; 6 Institute of Molecular Life Sciences, HUN-REN Research Centre for Natural Sciences, 1117 Budapest, Hungary; 7 Center for Cancer Research, Medical University of Vienna, 1090 Vienna, Austria; 8 Department of Metabolism, Digestion and Reproduction, Imperial College London, London, SW7 2AZ UK

**Keywords:** Ambient ionization, Mass spectrometry, Laser ablation, High-throughput

## Abstract

We introduce a novel automated well plate sampling system (AmbiSampler) using laser ablation-rapid evaporative ionization mass spectrometry (LA-REIMS) for label-free high-throughput biochemical screening for small volume biological fluid samples and cell line panels from multiwell plates. The human blood serum samples featured intensive signals in the low m/z range including fatty acids and metabolites and in the higher (m/z > 600) range associated with complex lipids, peptides, and proteins. We demonstrate the current quantification performance of the system through blood serum samples spiked with labelled amino acid mix. Cell line analysis was demonstrated by analyzing the NCI60 human cell lines panel grown in multiwell plates. Without any sample preparation, these experiments yielded rich metabolic and lipidomic mass spectrometry profiles, with classification results showing a 98.7% correct classification rate. We demonstrated that the new high-throughput setup using LA-REIMS technology provides an efficient and versatile approach for ambient ionization mass spectrometric analysis of various biological samples in a high-throughput manner.

**Graphical abstract:**

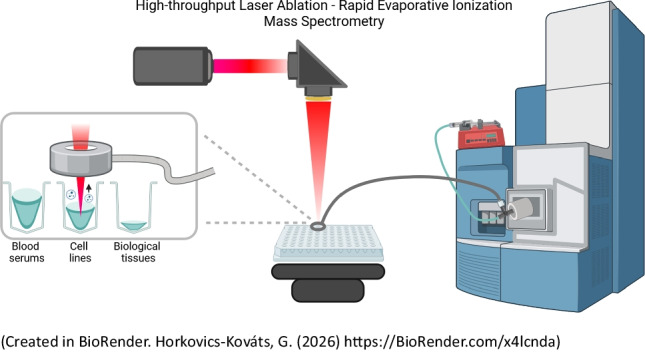

**Supplementary Information:**

The online version contains supplementary material available at 10.1007/s00216-026-06484-4.

## Introduction

The high-throughput and precise analysis of various biological samples is a basic requirement for many analytical laboratories worldwide. Since the introduction of multiwell microtiter plates in the 1960 s, numerous high-throughput screening methods have been developed using mass spectrometry in combination with automation technology for a variety of sample types [1, 2]. Important applications include the analysis of compound libraries, biological fluid samples with small volumes, or cell line panels [3–5], which can be screened against combinatorial drug libraries, reaction products, and affinity [6–8]. Today, there are multiple commercially available high-throughput platforms/ion sources that can be used in various application areas, ranging from standard autosamplers handling microplates to more innovative solutions like RapidFire (Agilent), DART JumpShot (Bruker), ECHO (Sciex), and Luxon (Phytronix). Several other desorption ionization techniques have also been further optimized for high-throughput applications, including the well-known matrix-assisted laser desorption ionization (MALDI) and desorption electrospray ionization (DESI) [8–10].

The RapidFire platform uses online solid-phase extraction (SPE) to extract compounds of interest and make the samples more suitable for liquid chromatography-mass spectrometry (LC-MS) [11]. Although this technique provides extensive information about the samples, the inclusion of SPE and LC (if present) significantly extends measurement time. Introduction of shotgun methods eliminated the use of LC, providing even higher throughput. With the emergence of ambient ionization methods, the requirement for sample preparation was also lifted, and the direct analysis of the cell cultures became possible. Direct analysis in real-time mass spectrometry (DART MS) is an ambient technique where sample ionization is achieved through a combination of Penning and chemical ionization at atmospheric pressure [12, 13]. By using a pulsed gas flow, ion formation in the sample environment is minimized, which improves the peak shape and signal-to-noise ratio of the measurement while reducing matrix effects and background noise. The ECHO system works with acoustic droplet ejection (ADE) to electrospray ionization (ESI)-MS via an open port interface (OPI) [14].

Introduction of lasers into high-throughput MS measurements represented a significant advancement. The ultrafast Luxon system, for instance, uses laser diode thermal desorption (LDTD) technology [15–17]. The samples are usually dried and positioned in wells on a metal sample holder, where they are desorbed by the heat produced by an infrared laser diode across the metal surface. The resulting gaseous neutral species are then transported into an atmospheric pressure chemical ionization (APCI) source mounted on a tandem mass spectrometer for ionization and subsequent analysis. This technique can provide information about the samples in seconds but is often hampered by thermal decomposition of the analytes of interest.

Although these commercially available techniques are widely used in forensics, quality control, food analysis, and the pharmaceutical industry, they still often require time-consuming and laborious sample preparation. Similar considerations apply in the case of matrix-assisted laser desorption ionization (MALDI) and a handful of other desorption ionization techniques. MALDI applications are among the most common examples, with both ultraviolet (UV) and IR-MALDI [18] being developed for high-throughput analysis of peptides and proteins. While there is still a need for sample preparation in the form of matrix application, MALDI-MS can directly analyze the prepared samples. The wide range of available laser wavelengths also enables the sampling and analysis of a broad range of samples [19, 20].

Moving further to increasingly popular ambient ionization techniques, desorption electrospray ionization (DESI) systems for instance require no sample preparation and have also been used in the field of high-throughput analysis [21]. Using pin tool transfer, samples ranging from substrates to tissue microarrays (TMA) were deposited on DESI plates for subsequent automated sampling [8, 22, 23]. Additional promising innovations in the field of ambient MS include hybrid laser and electrospray-based techniques like laser ablation electrospray ionization (LAESI) and infrared matrix-assisted laser desorption electrospray ionization (IR-MALDESI). The LAESI DP-1000 System from Protea Biosciences used mid-IR laser ablation for sample mobilization and combined this with ESI for post-ablation ionization. Also without the need of any sample preparation, the technique has been pushed towards high-throughput single-cell analysis among many other applications [24]. IR-MALDESI is a high-throughput technique that leverages the advantages of MALDI and ESI and is on the verge of commercial breakthrough [25–27]. By creating a thin layer of ice on the sample as an absorbing matrix, the mid-IR laser can desorb neutral molecules, which are subsequently ionized by electrospray in this case. The utility of the method has been proven in metabolite fingerprinting, detection of protein-ligand complexes and direct screening of enzyme activity [28–30]. The latest IR-MALDESI platforms have been equipped with a collaborative robotic plate transfer system to fully utilize the capabilities of high-throughput multiwell plate sampling [31].

**Fig. 1:**
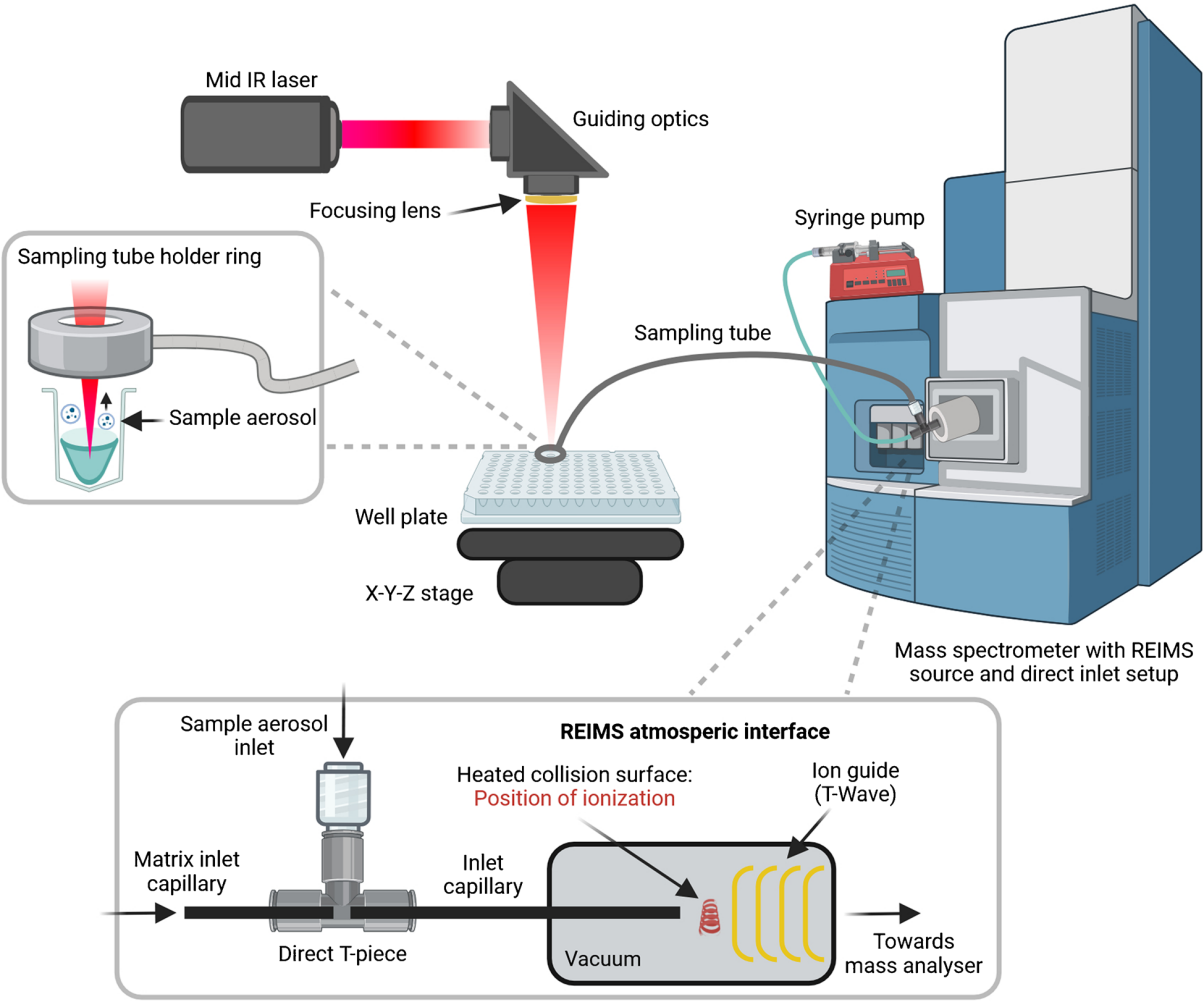
High-throughput LA-REIMS schematic showcase. LA-REIMS captures the generated sample aerosol after laser ablation through the vacuum suction of the mass spectrometer, mixes it with a carrier matrix in the direct T-piece, and guides the result to a heated collision surface (Kanthal D coil created from 1-mm-wide ribbon, coiled in a 3-mm-diameter coil to 5 mm long [34]) for ionization in the atmospheric interface before reaching the ion guide (Created in BioRender. Horkovics-Kováts, G. (2026) https://BioRender.com/s47votu)

Laser ablation-rapid evaporative ionization mass spectrometry (LA-REIMS) utilizing various lasers from the UV to IR range was originally developed for biological tissue analysis [32]. As an ambient MS technique, it requires no sample preparation due to the mobilization of the samples by means of laser ablation. This sample mobilization approach is similar to LAESI and IR-MALDESI. Yet, the method is fundamentally different due to the completely different ionization approach as LA-REIMS utilizes a heated metal surface embedded into the atmospheric interface for ionization [33] (Fig. 1). Collision of the sample with this heated metal surface induces molecule de-clustering leading to improved signal intensities and less contamination of ion optics. The exact function, aerosol kinetics, and the optimal interface geometry of the heated metal surface have been described by Simon et al. [34].

The first automated high-throughput REIMS setup was published in 2016, using electrically conductive stainless steel sampling probes on agar culture plates [35]. We have improved the system subsequently and equipped it with a carbon dioxide laser for the high-throughput analysis of bacterial cultures [36]. In the current study, we have constructed a new automated well plate sampling system (AmbiSampler) specifically designed to analyze sample sets in microplate format. The system uses an optical parametric oscillator (OPO) laser source operating at 2940 nm wavelength targeting the main infrared absorption band of water, providing additional improvement regarding sensitivity [37]. Earlier prototypes of the platform were described for coffee powder analysis [38], high-throughput drug stability assessment [39], and metabolic and lipidomic profiling of live cells [40]; however, the exact mechanisms of the aerosol formation, the aerosol-laser interactions, and high-throughput biological applications were not described in this context.

In this manuscript, we present our work in developing a robust and reliable technological platform that can perform the rapid analysis of biological samples from common sample carrier formats, without the need for additional sample preparation steps. First, we show that we can analyze multiple different molecule types with our technology. Once the optimal setup was established, to demonstrate the system’s capabilities, proof-of-concept studies were performed using HEK, K562 cell lines, and the NCI60 cell panel. These experiments demonstrate that the AmbiSampler is a powerful analytical tool capable of rapid, time-effective, and cost-effective metabolic classification of complex biological systems.

## Experimental

### Materials

Roswell Park Memorial Institute (RPMI) 1640 medium, phosphate buffer saline (PBS) (Gibco, Thermo Fisher Scientific, Waltham, MA, USA), penicillin, and streptomycin (Life Technologies, Carlsbad, CA, USA) were used for cell culturing. The mass spectrometers used in the experiments were calibrated according to manufacturer protocols (Waters) using sodium formate solution (Alfa Aesar GmbH & Co KG, Karlsruhe, Germany) (500 µM in isopropanol:water 9:1) induced by a Harvard Elite plus 11 syringe pump (Harvard Apparatus, Holliston, MA 01746, USA) with a 0.05 mL/min flow rate using a 1-mL gastight syringe (Hamilton Bonudaz AG Europe, Via Crusch 8 CH-7402 Bonudaz, GR, Switzerland) into the REIMS source. Only calibration profiles where residual mass at 95% has > 3 ppm accuracy were accepted. To ensure mass measurement accuracy, lock mass from known internal standards was applied on the measurements, which were performed on the calibrated instruments. As internal standard, either leucine-enkephalin (exact mass 554.2615 m/z in negative ion mode) (Thermo Fisher Scientific, Waltham, MA, USA) was added to the sample matrix or usually encountered compounds like palmitic acid (exact mass 255.2324 in negative ion mode) were used as lock mass.

### Instrumentation

The AmbiSampler platform was constructed using a 3D-printed well plate holder socket on motorized X-Y translational stages to perform high-throughput mass spectrometry measurements. A commercially available Opolette HE 2940 Optical Parametric Oscillator (Opotek LLC, Carlsbad, CA, USA) laser was used for sample mobilization from well plates at a wavelength of 2940 nm and pulse peak energy of 6 mJ at 20 Hz. The optical path consisted of a mirror (PFE-P01, Thorlabs Inc., Newton, NJ, USA) and plano convex CaF_2_ focusing lens (1″, 50 mm focal length (LA5763, Thorlabs Inc., Newton, NJ, USA). A 1-m-long polytetrafluoroethylene (PTFE) tube with an outer diameter of 2.2 mm (OD2.2) and an inner diameter of 1.6 mm (ID1.6) was positioned above the well for aerosol collection, achieved through the vacuum suction of the mass spectrometer, and connected to the REIMS interface through a custom-built direct T inlet (Waters Research Center, Budapest, Hungary). Here, the aerosol was mixed with isopropanol containing 180 µM leucine-enkephalin in front of the MS inlet capillary at a flow rate of 150 µL/min. The setup was coupled to a Xevo™ G2-XS Q-TOF MS (Waters Corporation, Milford, MA, USA) for sample analysis. Corresponding sample acquisition time is stated at the experiments where needed.

### High-throughput and carryover assessment

To assess the high-throughput capabilities and sample carryover of the AmbiSampler system, eight compounds (creatine, glucose, glutamine, leucine-enkephalin, 9-aminoacridine, indigo carmine, acid yellow 3, and green S) were measured from filter bottom 96-well plates (Thermo Fisher Scientific, Waltham, MA, USA) in negative ion mode. All compounds were solvated in water. Then, 10 µL solution of each compound was pipetted to the corresponding well from 1 to 12 in a row. Wells 3, 6, 9, and 12 were left as blanks. This experiment was repeated on three different settings: 8 samples (including 4 blanks) in 12, 6, and 3 s. In the post-processing, the signals corresponding to individual compounds were extracted from the chromatogram to assess sample carryover.

### Application demonstration: pilot compound coverage study

To demonstrate the capability of analyzing different samples with the AmbiSampler system, the following samples were measured in negative ion mode: commercially acquired randomized AB serum from healthy donors (Bayrische Rote Kreuz, Regensburg, Germany), NCI60 cell line panel purchased from DCTD Tumor Repository (National Cancer Institute, Frederick, MD), Mouse brain was taken and sectioned by trained personnel under the regulations of the German Animal Welfare Act (Tierschutzgesetz, TierSchG, §4, section 3) from a mouse that was bred in-house at the animal facility of the University of Regensburg and maintained under standard laboratory conditions; for positive ion mode representation, the MassPREP Peptide Mixture (Waters, Milford, MA, USA) was used.

### Quantitative blood serum analysis approach

Commercially acquired randomized AB serum from healthy donors (Bayrische Rote Kreuz, Regensburg, Germany) was used for the blood serum analysis experiment containing 3 parallels and 8 wells per run in a standard shallow, round-shaped, V-bottom 96 well plate (Merck, Darmstadt, Germany). Each well was filled with 45 µL serum and supplemented with 5 µL differently diluted canonical amino acid mix, comprising 20 uniformly 13C/15N labelled amino acids (Cambridge Isotope Laboratories Inc., MA, USA), with LC-MS grade water. The mix covers glycine, l-alanine, l-arginine, l-asparagine, l-aspartic acid, l-cystine, l-glutamic acid, l-glutamine, l-histidine, l-isoleucine, L-leucine, l-lysine, l-methionine, l-phenylalanine, l-proline, l-serine, l-threonine, l-tryptophan, l-tyrosine, and l-valine. The final concentrations of each amino acid mix/water in the serum per well were the following: well 1 = 5 µM, well 2 = 25 µM, well 3 = 50 µM, well 4 = 75 µM, well 5 = 100 µM, well 6 = 150 µM, well 7 = 200 µM, well 8 = 250 µM. The in-total 50-µL filled wells were measured with the AmbiSampler system.

### Experimental setup optimization for cell analysis

To determine the lower limit of evaluable spectra from cells, HEK cells (ATCC Collection, Manassas, VA) were measured with the AmbiSampler at 90 mW, 20 Hz laser emission for 3 s on various cell counts (31,000; 62,000; 125,000; 250,000; and 500,000 cells) in 50 µL water in standard shallow, round shaped, V-bottom 96 well plates (Merck, Darmstadt, Germany).

To visualize aerosol formation from liquid (water), the LA-REIMS system was extended with a green laser operated on 100 mW and was spread in a horizontal plane by a line-generating optics. With this, the generated aerosol was scanned in the vertical direction in the 0–50 mm range above the sample surface. The results were video recorded.

To understand the behavior of liquid samples in wells under laser exposure and to visualize the corresponding droplet distribution, filter paper was positioned 10 mm above the well during the experiment to capture flying back droplets. Here, the experiment was performed with a standard square-shaped 96 deep well plate with V-bottom (Merck, Darmstadt, Germany). The filter paper was punctured in the middle to ensure obstacle-free passage of the laser beam. The exact size of the hole was not documented; however, care was taken to ensure that the laser beam did not collide with the filter paper and thus lose energy. This was always verified by measuring the laser energy before each measurement. Here, the laser beam was focused onto the sample surface for each setting. The following result figures will represent the distance of well surface to the sample surface as sample level. Thus, the following sample levels were used in this experiment: 1.8 mm, 8 mm, 24 mm, and 36 mm. To aid the visualization, sodium fluorescein (Merck, Darmstadt, Germany) was dissolved in water and the resulting fluorescent droplet traces on the filter paper were visualized using an LED operating at 405 nm and documented by photography.

In order to elucidate the influence of the sample level on MS signal intensity and to determine a potentially optimal sample level, the aerosol generated by LA-REIMS from 250,000 cells/50 µL water K562 cell line (ATCC Collection, Manassas, VA) was introduced into the mass spectrometer and the signal was monitored. Here, various sample levels from standard square-shaped 96 deep well plates with V-bottom (Merck, Darmstadt, Germany) (investigated sample levels: 1.8 mm, 5 mm, 8 mm, 10.9 mm, 13.9 mm, 17.0 mm, 20.3 mm, 23.7 mm, 27.2 mm, 30.7 mm, 34.2 mm, and 36.1 mm) and standard square-shaped 384 deep well plates with V-bottom (Merck, Darmstadt, Germany) were investigated (tested sample levels: 1 mm, 2 mm, 4 mm, 8 mm, 11 mm, 13 mm).

Lastly, the influence of the focal position of the optical lens was observed by monitoring MS signal from 250,000 cells/50 µL water K562 cell line (ATCC Collection, Manassas, VA) sampling focusing on and below sample surface from standard square shaped 96 deep well plate with V-bottom (Merck, Darmstadt, Germany) (focus below sample surface: 13 mm, 11 mm, 9 mm, 7 mm, 5 mm, 3 mm, 1 mm and laser focus directly on sample).

### NCI60 cell line analysis

NCI60 cell lines were purchased from DCTD Tumor Repository (National Cancer Institute, Frederick, MD). Cells were cultured in RPMI 1640 medium supplemented with 10% (V/V) fetal bovine serum (Gibco, Thermo Fisher Scientific, Waltham, MA, USA), 2 mM l-glutamine, 100 units/mL penicillin, and 100 µg/mL streptomycin. Cells were cultured at 37 °C with 5% CO_2_ until ~ 90% confluency. Each cell line was grown in three different 75 cm^2^ flasks and was harvested two times with 1 week difference. The harvested cells were collected in a Falcon tube and centrifuged with 350 g for 5 min, the supernatant was discarded, the cells were resuspended in 13 mL PBS, then centrifuged again, and then the supernatant was completely removed by vacuum aspiration. The residual cell pellet (~ 40 µL, 6–8 × 10^6^ cells) was resuspended in 100 µL sterile MQ water, and 50 µL of this sample was pipetted into two 96 shallow bottom well plates, the first plate being the training set and the second plate being the test set. Here, cells were measured with the LA-REIMS AmbiSampler setup with 40-s-long laser emission for each well at 0.5 s MS scan time, to achieve as much data as possible for database building.

### Data processing and analysis

Mass spectrometric data was acquired in negative ion mode, 0.5 s MS scan time (unless stated otherwise) 50–1200 mass to charge range (unless stated otherwise), visualized using MassLynx (Waters Corporation, Wilmslow, UK), and processed with an in-house built software Abstract Model Builder (AMX, [Beta] version 1.0.2273.0, Waters Research Center, Budapest, Hungary) using multivariate statistics including principal component analysis (PCA) followed by linear discriminant analysis (LDA). Data was acquired in positive ion mode and 1.0 s/scan time for compound coverage of peptides. Molecules from cell lines were identified by using exact mass and MS/MS measurements [41].

## Results and discussion

### High-throughput and carryover assessment

**Fig. 2:**
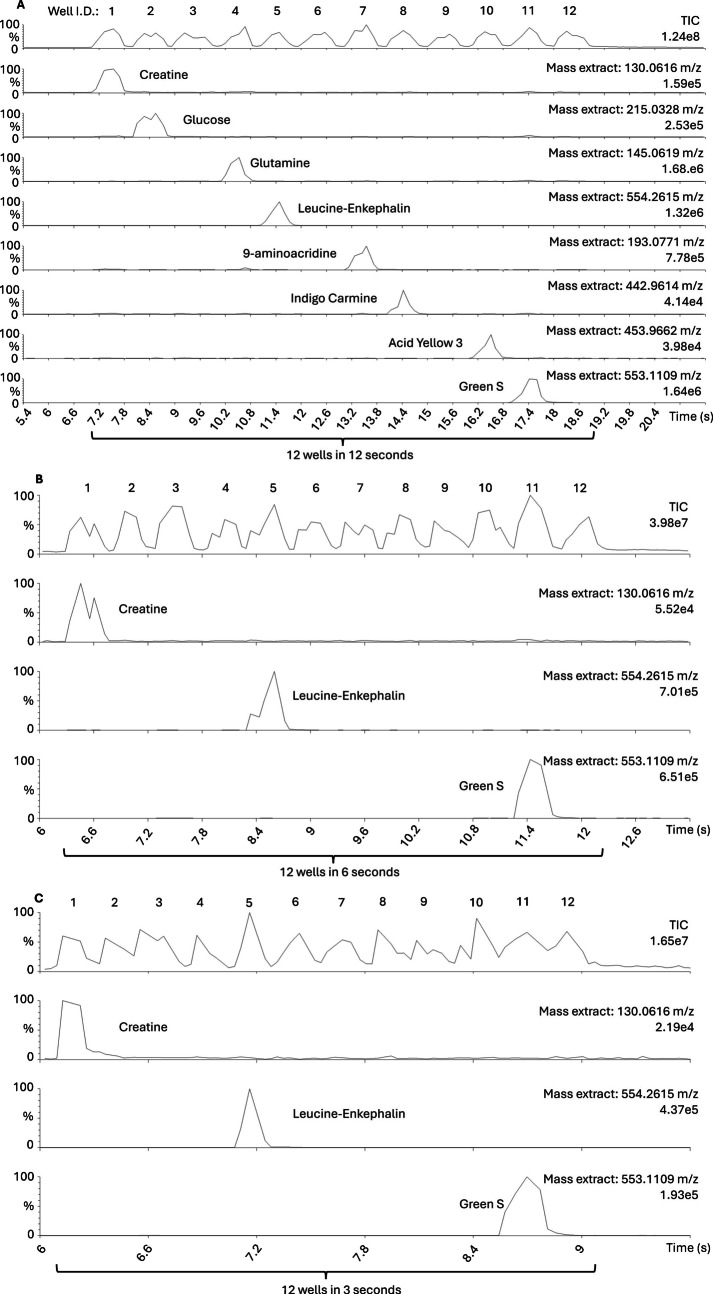
Mass spectrometry analysis via AmbiSampler of compound set containing creatine, glucose, glutamine, leucine-enkephalin, 9-aminoacridine, indigo carmine, acid yellow 3, and green S with 4 additional blanks. **A** 12 wells were measured in 12 s at 20 scans/s acquisition speed. **B** 12 wells were measured in 6 s at 20 scans/s acquisition speed (extracted compounds focusing on creatine, leucine-enkephalin, and green S). **C** 12 wells were measured in 3 s at 30 scans/s acquisition speed (extracted compounds focusing on creatine, leucine-enkephalin, and green S)

Measuring 12 wells in 12 s (Table 1) showed a good baseline retention without any observable sample carryover in the total ion count (TIC) representation (Fig. 2A, Fig. S1).

**Table 1:** Compound list for high-throughput and carryover assessment: measured compounds in filter bottom 96 well plates with corresponding concentration and well position including blanks

Compound	Adduct	Exact mass	Concentration	Well ID
Creatine	[M-H]^−^	130.0616	1 mg/mL	1
Glucose	[M-Cl]^−^	215.0328	1 mg/mL	2
Blank control	-	-	-	3
Glutamine	[M-H]^−^	145.0619	1 mg/mL	4
Leucine-Enkephalin	[M-H]^−^	554.2615	0.1 mg/mL	5
Blank control	-	-	-	6
9-Aminoacridine	[M-H]^−^	193.0771	1 mg/mL	7
Indigo carmine	[M-2H + Na]^−^	442.9614	1 mg/mL	8
Blank control	-	-	-	9
Acid yellow 3	[M-2H + Na]^−^	453.9662 m/z	1 mg/mL	10
Green S	[M-Na]⁻	553.1109 m/z	1 mg/mL	11
Blank control	-	-	-	12

Measuring 12 wells in 6 s (Fig. 2B) also showed good baseline retention without any observable sample carryover. The last setting with 12 wells in 3 s (Fig. 2C) showed limited baseline retention but still without observable sample carryover. Further triplicate measurements of 12 wells in 4.8 s also showed limited baseline retention but still without observable sample carryover (Fig. S2A-C).

### Application demonstration: pilot compound coverage study

**Fig. 3:**
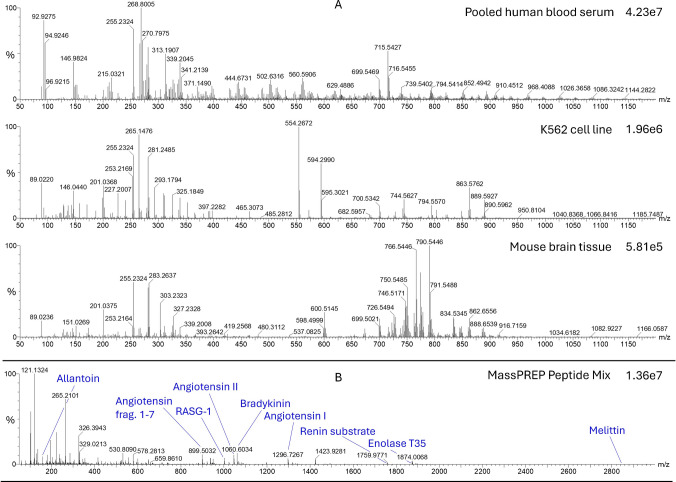
**A** Measured biological samples: pooled human blood serum, cell line K562, and mouse brain tissue (negative ion mode, combined spectra of 5 scans, lock massed on palmitic acid—255.2324, 50–1200 m/z). **B** MassPREP Peptide Mix (positive ion mode, combined spectra of 5 scans, 50–3000 m/z)

The AmbiSampler system is capable of LA-REIMS analysis of arbitrary samples, including biological fluids, cells, and tissues, without sample preparation. Figure 3A shows spectra of pooled human blood serum, cell line K562, and mouse brain samples in negative ion mode featuring intensive signals in the low m/z range associated with fatty acids and metabolites and in the higher (m/z > 600) range associated with complex lipids and peptides. The corresponding peak annotations are provided in the supplementary material and are based on our in-house database, which has been validated through liquid chromatography (Table S1, Table S2, Table S3) [40]. Furthermore, a combined spectrum is presented of detected compounds from the MassPREP peptide standard mix in positive ion mode. Expected compounds according to the manufacturer are Allantoin, RASG-1, Angiotensin frag. 1–7, Bradykinin, Angiotensin II, Angiotensin I, Renin substrate, Enolase T35, Enolase T37 (Table S4). As the data demonstrates, the method is capable of comprehensively detecting protein-forming amino acids and many lipid species having a significant role in mammalian metabolism, illustrating the ability of the system to provide broad lipidomic and metabolic profiles for various biomedical sample types in negative and positive ion modes. The spectra include some commonly known components which are represented as follows: either as full names (e.g., lactate, glutamate) or commonly used abbreviations containing lipid class (total number of carbons: total number of double bonds) or lipid class (P—total number of carbons: total number of double bonds) while P stands for the plasmalogen form (vinyl ether linkage at the sn-1 position). Lipid species encountered within this study were abbreviated as follows: phosphatidic acid (PA), phosphatidylcholine (PC), phosphatidylethanolamine (PE), phosphatidylserine (PS), phosphatidylglycerol (PG), phosphatidylinositol (PI), phosphatidyl threonine (PT), ceramides (Cer), sphingomyelin (SM), diacylglycerol (DG).

To check the respective amino acid content of pooled human blood serum, cell line K562, and mouse brain tissue, the spectra were analyzed and the corresponding signal intensities were extracted (Table 2).

**Table 2:** Detected amino acids in pooled human blood serum, cell line K562, and mouse brain tissue with corresponding detected signal intensities (-, not detected; o, detected, but very low signal intensity)

Detected amino acid	Exact mass in negative ion mode (m/z)	Pooled human blood serum	K562 cell line	Mouse brain tissue
Glycine	74.0247	1.96e4	-	1.20e3
Alanine	88.0404	3.59e4	4.01e3	5.77e3
Serine	104.0353	9.89e4	3.70e3	5.27e3
Proline	114.0560	5.39e4	o	o
Valine	116.0717	1.74e5	-	-
Threonine	118.0509	2.64e5	-	3.66e3
Cysteine	120.0124	-	-	1.22e3
Iso/Leucine	130.0873	3.67e5	7.81e3	7.73e3
Asparagine	131.0462	-	1.21e4	-
Aspartate	132.0302	-	3.65e4	9.51e3
Glutamine	145.0618	1.04e5	9.77e4	4.86e4
Lysine	145.0982	1.04e5	o	o
Glutamate	146.0458	2.51e5	5.87e5	2.49e4
Methionine	148.0437	6.08e5	-	3.52e3
Histidine	154.0622	5.83e5	1.38e4	2.41e3
Phenylalanine	164.0717	2.45e5	2.11e3	8.23e3
Arginine	173.1043	1.14e5	-	-
Tyrosine	180.0666	1.23e5	3.26e3	3.46e3
Tryptophan	203.0826	-	-	-

### Quantitative blood serum analysis approach

**Fig. 4:**
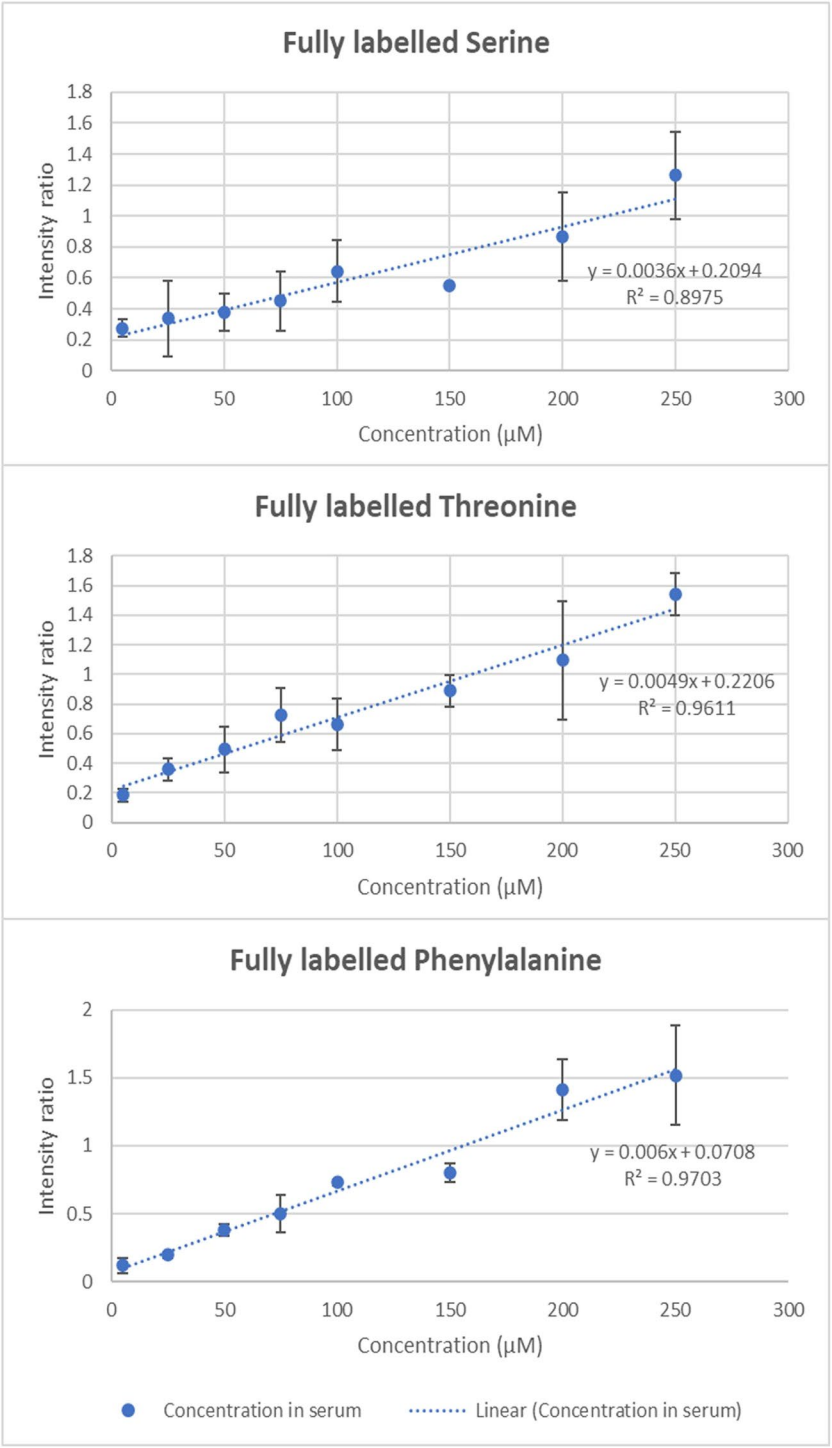
Calibration curves for serine, threonine, and phenylalanine using blood serum samples spiked with labelled amino acid mix with the automated LA-REIMS methodology. The curves depict the intensity ratio of labelled amino acid added to aliquots of a pooled serum sample and the native amino acid present in the sample

The quantification performance of the system was tested using blood serum samples spiked with fully labelled amino acid mix (Fig. 4). Since we could not remove the native (unlabelled) amino acid from the pooled serum sample, we added the mixed isotope labelled amino acid mixture at different levels and used the native molecule as an internal standard present at the same concentration in all samples. The calibration curves depict the labelled amino acid intensity divided by the intensity of the unlabelled molecule. This approach was taken because no other separation procedures were performed and concerns arose about potentially uncontrolled adduct formation and matrix effects due to the complex biological matrix and its impact on signal stability when using a non-targeted method. In addition, isotopically labelled amino acid is chemically equivalent, so the adduct reaction factors, adduct formation, ionization yield, and non-specific binding should all be the same. In this way, the external standard should also be irrelevant. The data clearly demonstrates that the method can successfully be calibrated in case of metabolites in their native concentration range, illustrating the feasibility of using the method and instrument for chemical analysis. Deeper insights into semi-quantitative capabilities of the system have been described using HPLC-UV-MS in high-throughput drug stability assessment [39].

### Experimental setup optimization for cell analysis

**Fig. 5:**
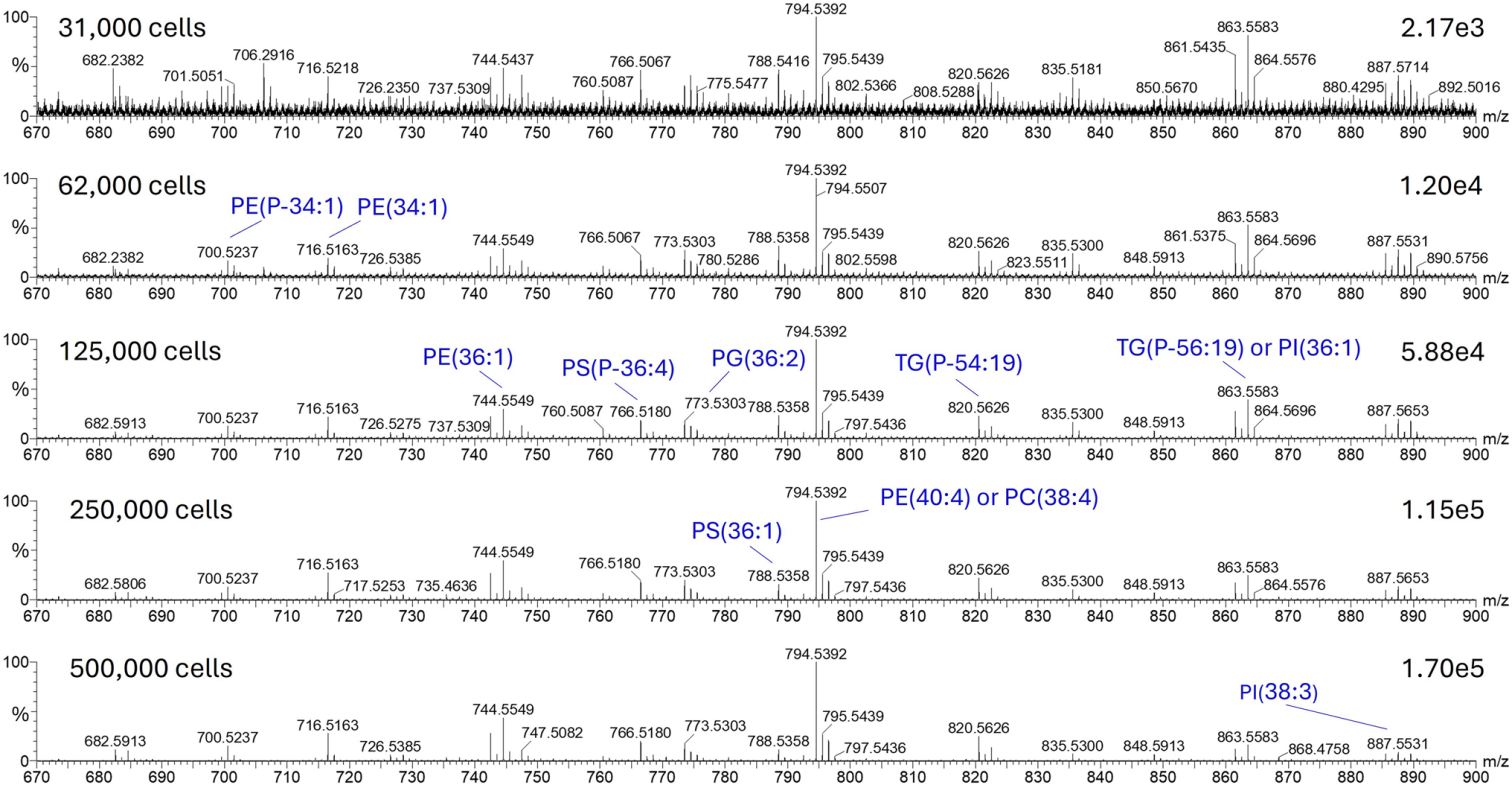
LA-REIMS cell analysis of HEK cell line with different cell counts: 31,000; 62,000; 125,000; 250,000; and 500,000 cells/50 µL water (negative ion mode, combined spectra of 5 scans with the showcase in the range of 670–900 m/z to focus on phospholipid region, 20 Hz sampling frequency, 3 s sampling time)

To demonstrate the capability of the system for high-throughput cell analysis, we have analyzed cell pellets from suspensions of K562 cells directly from standard shallow, V-bottom 96 well plates (Merck, Darmstadt, Germany). Better quality signal was obtained for K562 than for HEK, demonstrating the variance in signal intensity and detection limit between cell types. Good quality signal was observed from 62,000 cells/50 µL water onwards (Fig. 5). This indicates that the absolute cell number can be reduced to 12,400 cells by analyzing only 10 µL of sample. It is important to note that from this amount only a limited amount of scans can be acquired. In all further experiments, 250,000 cells/50 µL water were used to not work in the lower limit of detection.

**Fig. 6:**
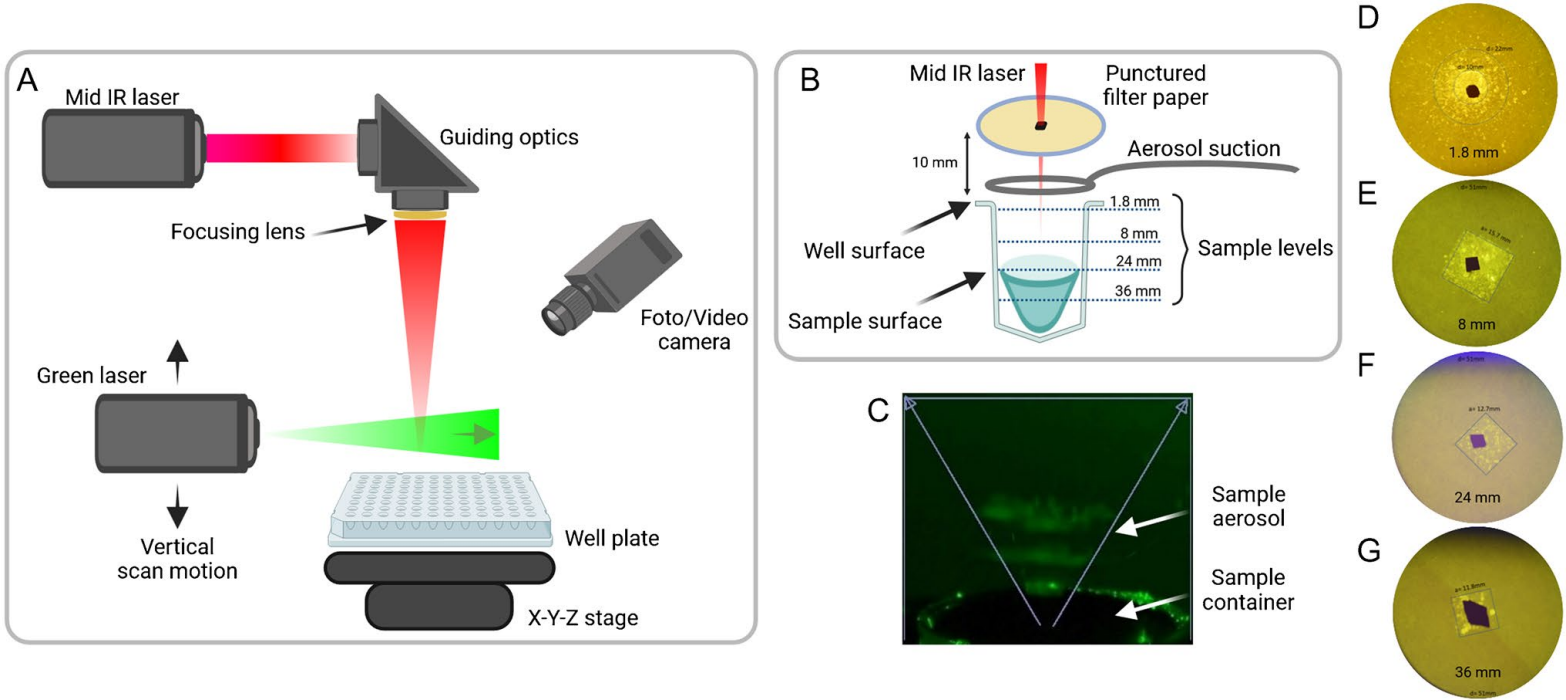
**A** LA-REIMS setup schematic for monitoring the generated aerosol behavior via green laser and video/photo capture in the area above the liquid irradiated by the mid IR laser. **B** Tested sample levels (1.8 mm, 8 mm, 24 mm, 36 mm) from well with a punctured filter paper above the well plate (distance 10 mm). As the laser was focused on the sample surface, the well plate was moved accordingly; therefore, larger punctured holes were needed to prevent laser energy loss. **C** Monitored aerosol jet with 70° spread within the well caught on punctured filter paper placed above the well for **D** 1. 8 mm sample level, **E** 8 mm sample level, **F** 24 mm sample level, and **G** 36 mm sample level (Created in BioRender. Horkovics-Kováts, G. (2026) https://BioRender.com/fp4j1rk)

During the experiment, to understand the behavior of liquid samples in wells under laser exposure and to visualize the corresponding droplet distribution, a standard square-shaped 96 deep well plate with V-bottom was used (Merck, Darmstadt, Germany). In a full well, the jet from the sample surface extended upwards at an angle of 70° resulting in a circular pattern on the filter paper (Fig. 6C, D). On the contrary, at deeper sample levels, the distribution of the droplets showed a square-shaped form on the filter paper due to the restricting effect of the wall of the well (Fig. 6E–G).

**Fig. 7:**
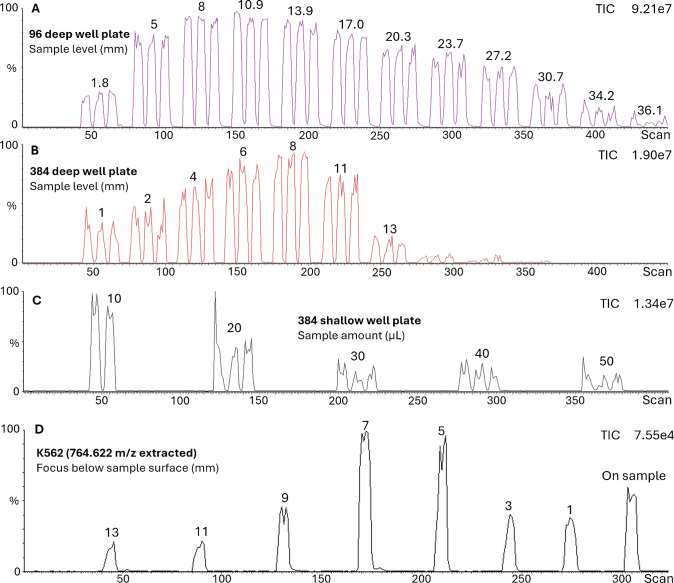
TIC representation of LA-REIMS sampling from **A** 96 and **B** 384 deep well plate on various sample levels, from **C** 384 micro-well plate with various sample amounts and from **D** K562 suspensions directly from 96 well plates with different laser focus settings below the sample surface

The most intensive MS signal was recorded with the sample level 8 to 10 mm for 96 and 384 well plate (Fig. 7A, B). When the well is full, the aerosol is less effectively collected by the sampling tube which is in the ring (sampling point) due to the angle of the initial aerosol plume formation. If the sample level is near the bottom (e.g., sample level 36 mm in 96 deep well plate), the droplet formation occurs deep in the well and the efficiency of the sample collection decreases. For shallow well plates, it is important to note that the optimal sample level of 8 mm can only be set with minimal sample volume which is limited by the well capacity. This volume is only enough for a few seconds of sampling within a well (Fig. 7C). At an aerosol jet angle of about 70°, the theoretical diameter of the aerosol jet should be 2.8 mm when it reaches the height of the sampling point, when the well is full. Note that the exact sampling point position (~ 2 mm above well surface) is the suction tube opening on the side of the sampling tube holder ring (Fig. 1) which has an inner diameter of ~ 8.6 mm similar to the inner diameter of a well. Calculating further, the diameter of the aerosol jet at a sample level of 6.4 mm should be equal to the diameter of the well (~ 8.6 mm). More efficient detection is achieved with deeper sample levels as the jet angle spread hits the wall of the well and therefore exits the plate in the well’s shape (Fig. 6D–G). This aerosol shape harmonizes with the inner diameter of the ring, resulting in higher acquired signal intensities. Measuring with laser focus at and below sample surface showed an increase in signal intensity when focusing below the sample surface between 5 and 7 mm (sampling depth) (Fig. 7D). This parameter can be considered for further optimization in case of deep well plates and suggests that a lower energy density due to laser defocusing can also be sufficient for sample mobilization.

For each well plate type, there is an optimal sample level, which was determined to be 8 mm in correlation with our findings using different sample types in deep well plates (Fig. 7A–C). Furthermore, focusing below sample surface can improve signal quality (Fig. 7D). Even though the AmbiSampler can deliver adequate spectral results under non-optimized conditions, we recommend optimizing the system for the use of different well plate types where desired or necessary.

After examining this parameter space, we performed a large-scale study on cancer cell lines for the evaluation of reproducibility and repeatability.

### NCI60 cell line analysis

**Fig. 8:**
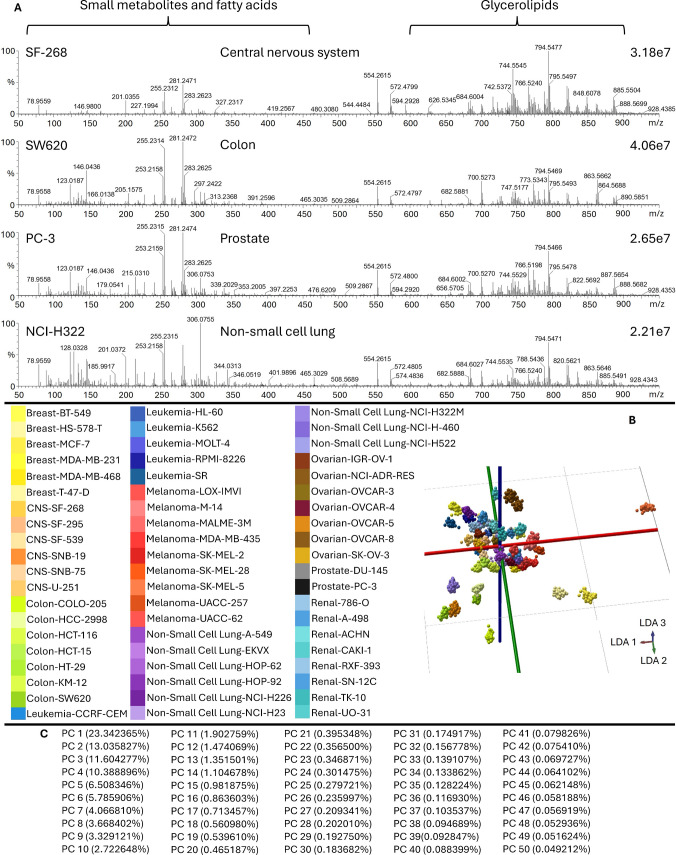
**A** Combined spectra of 50 scans from selected LA-REIMS measurements from NCI60 cell line panel (SF-268 central nervous system carcinoma, SW620 colon carcinoma, PC-3 prostate carcinoma, and NCI-H322 non-small cell lung carcinoma). **B** Pseudo-3D LDA model of sampled NCI60 data set in the 670–1000 m/z range with 0.1 binning. **C** PC variance of sampled NCI60 data set in the 670–1000 m/z range with 0.1 binning

**Fig. 9:**
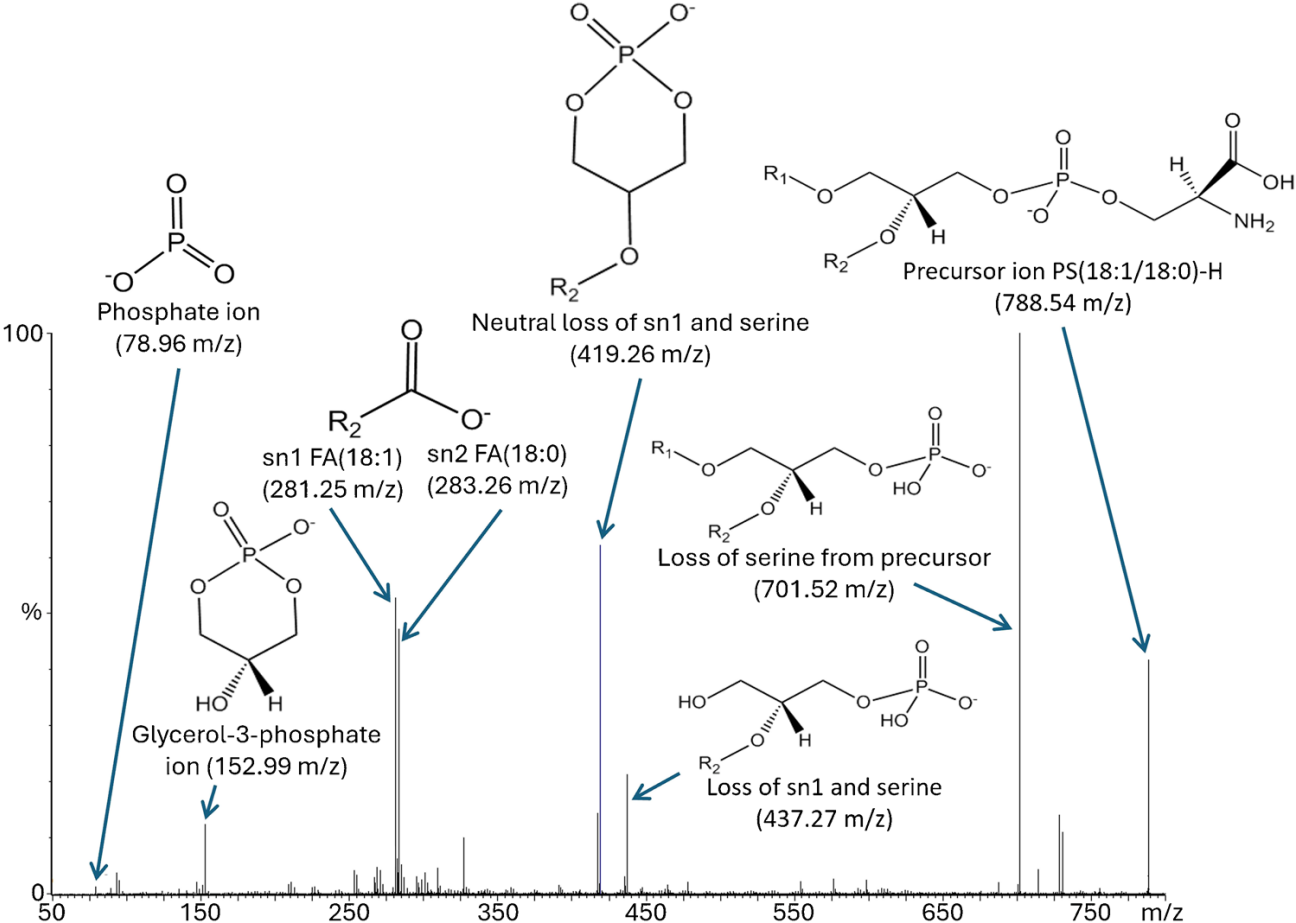
MSMS peak identification example for 788 m/z—PS (36:1) from the NCI60 panel, identified by MSMS, exact mass measurements, and LipidMaps database

In the late 1980 s, the US National Cancer Institute (NCI) sought to replace animal tumor models with human tumor cell lines for drug screening. The cell lines represent leukemia, melanoma, non-small lung cancer, and cancers of the colon, brain, ovary, breast, kidney, and prostate. Over time, the NCI60 panel has become a powerful tool for cancer research and the development of effective drugs [42], with in-depth analysis of the proteome, transcriptome, and gene mutations of cell lines [43, 44]. Lipidomic analysis of the entire panel was performed using REIMS and bipolar forceps, which provided detailed lipidomic information about the cell lines [45]. These results underlined the importance of lipidome analysis, both in the context of lipid biochemistry and a means for phenotyping. Although the obtained data was meaningful, the methodical process required a considerable amount of time, as it was designed to be a reproducibility study. The total of 997 samples were analyzed resulting in 7072 spectra acquired in 18 different batches throughout 6 months. Test spectra were acquired in separate batches resulting in a set of 467 samples and 3339 spectra. Experiments using the new system resulted in similar, rich metabolic and lipidomic profiles (Fig. 8A). Model building was performed using 72 PCA dimensions and 59 LDA dimensions in the 670–1000 m/z range at 0.1 binning (Fig. 8B, Fig. S3). Seventy-two PCA dimensions were selected, because they yielded the best cross-validation results. Fifty-nine LDA dimensions were the maximum number of LDA components, having a total of 60 classes. The 670–1000 m/z range was selected to fully focus on the lipid region and to avoid interfering adducted media in the lower 600 m/z range. As there are 60 classes to separate, the overall variance is already relatively low in the principal components (Fig. 8C). Cell line–identification capabilities were demonstrated using a sample cohort of 470 spectra not utilized for model building. Four hundred sixty-seven out of 470 samples were classified correctly (98.7% correct classification rate). A 100% correct cross-validation score was achieved when using the “leave 20% out” cross-validation method for the full dataset. Misidentifications of the test set occurred in a few samples of four cell lines: 786-0 (renal), A549 (non-small cell lung), HCT-116 (colon), and NCI-H522 (non-small cell lung) cell lines. The 786-0 renal cell line was also identified as the DU-145 (prostate) cell line, the A549 cell line was identified as the HL-60 leukemia cell line, and another non-small lung cell line HOP-62. The HCT-116 colon cell line was identified as MDA-MB-435 melanoma cell line, while the NCI-H522 cell line was identified as another non-small cell lung cell line NCI-H23. Additionally, the results were examined at the molecular level revealing characteristic ceramides and several phospholipids such as PEs, PCs, and PIs for the cell lines (Fig. 9, Table 3). In addition, molecules specific for only a few cell lines, such as SMs, PGs, Pas, and PT, were also identified. This large-scale study has shown that we can reproducibly acquire within seconds a rich and unique lipid profile from cell line samples using our novel technology.

**Table 3:** Summary of all identified species from the NCI60 panel

Measured Mass	Abundant in Cell Lines	Theoretical Mass	Identified Species	Adduct
656.573	HT29, OVCAR-3	656.575	Cer(40:1)	[M+Cl]^-^
682.590	MCF7, A549, MOLT-4	682.591	Cer(d42:2)	[M+Cl]^-^
684.602	ACNH, A549, HT29	684.607	Cer(d42:1)	[M+Cl]^-^
687.544	ACHN, MCF7	687.545	PE-Cer(d36:1)	[M-H]^-^
700.528	K562, SR, HT29, OVCAR-3	700.529	PE(P-34:1)	[M-H]^-^
714.510	OVCAR-3, ACHN	714.508	PE(34:0)	[M-H]^-^
716.525	All Cell Lines	716.524	PE(34:1)	[M-H]^-^
728.558	SN12C, KM12	728.560	PE(P-36:1)	[M-H]^-^
737.538	SN12C, KM12, HT29, ACHN	737.537	SM(d34:1)	[M+Cl]^-^
742.541	MCF7	742.539	PE(36:2)	[M-H]^-^
744.555	All Cell Lines	744.555	PE(36:1)	[M-H]^-^
747.516	ACHN, A549	747.518	PG(34:1) PA(40:6 check )	[M-H]^-^
770.571	ACHN, A549, MOLT-4	770.571	PE(38:2)	[M-H]^-^
773.535	HCT-116	773.534	PG(36:2)	[M-H]^-^
788.542	MCF7	788.545	PS(36:1)	[M-H]^-^
794.547	All Cell Lines	794.547	PC(34:1)	[M+Cl]^-^
796.548	All Cell Lines	796.550	PC(34:0)	[M+Cl]^-^
802.562	ACHN	802.560	PT(36:1)	[M-H]^-^
820.560	All Cell Lines	820.563	PC(36:2)	[M+Cl]^-^
822.570	K562, SR	822.565	PC(36:1)	[M+Cl]^-^
848.600	MCF7	848.590	PC(38:2)	[M+Cl]^-^
861.551	HCT-116, MOLT-4, KM12, SN12C	861.550	PI(36:2)	[M-H]^-^
863.566	All Cell Lines	863.566	PI(36:1)	[M-H]^-^
885.550	HCT116, ACHN	885.550	PI(38:4)	[M-H]^-^
887.566	K562, NCI-H23, OVCAR-3	887.566	PI(38:3)	[M-H]^-^
889.581	MCF-7	889.581	PI(38:2)	[M-H]^-^

## Conclusions

We have successfully developed and characterized an automated plate sampling system for ambient LA-REIMS that is capable of high-throughput and sample preparation–free analysis of industrial well plates for native samples. We were able to successfully obtain metabolic profiles from several sample types including cell lines, biofluids, and biopsy samples and assess the feasibility of quantification of amino acids in human blood serum. The applicability was demonstrated using a method for measuring the NCI60 cell line panel pilot project, which study showed that the system is capable of rapid, large-scale, high-throughput mass spectral analysis.

The strength of the AmbiSampler platform lies in the high-throughput analysis of native samples under ambient conditions without the need for any sample preparation. A 2 Hz analysis frequency was successfully achieved, and since no sample carryover was detected in these experiments, even 4 Hz is likely to be feasible with a faster and more sensitive mass spectrometer. Using the data obtained from the platform, we were able to successfully characterize and classify with high accuracy the whole NCI60 cancer cell line panel (98.7% correct classification rate).

These results show that our platform can be a powerful tool in molecular biology, metabolic research, and any high-throughput applications where little sample preparation and rapid turnaround times are desired.

## Supplementary Information

Below is the link to the electronic supplementary material.

Supplementary file1 (DOCX 2.44 MB)

## Data Availability

All data will be made available upon request to the corresponding authors.
