# Supplementary material for: Characterization of blood serum and cell lines by an automated high-throughput well plate sampling system using laser ablation-rapid evaporative ionization mass spectrometry
Source: Anal Bioanal Chem. 2026 Apr 28;418(12):3773–89. doi: 10.1007/s00216-026-06484-4 (PMC13221428; doi:10.1007/s00216-026-06484-4)
Supplement: Supplementary file 1 — Supplementary file1 (DOCX 2.44 MB) [file 216_2026_6484_MOESM1_ESM.docx]

Supplementary Information

Characterization of blood serum and cell lines by an automated high-throughput well plate sampling system using Laser Ablation - Rapid Evaporative Ionization Mass Spectrometry

Gabriel S. Horkovics-Kováts^1,2,3^, Daniel Simon^4,8^, Richárd Schäffer^2^, István Pap^2,4^, Zahra Nozari^4^, Alexander König^4^, Sonja Decking-Paede^5^, Roland Szand^2^, Adrienn Molnár^1,2,3^, Gitta Schlosser^3^, Nóra Kucsma^6^, Gergely Szakács^6,7^ Zoltán Takáts^4,8^* and Júlia Balog^2^*

^1^Hevesy György PhD School of Chemistry, ELTE Eötvös Loránd University, H-1117 Budapest, Hungary; ^2^Waters Research Center, H-1031 Budapest, Hungary; ^3^MTA-ELTE Lendület (Momentum) Ion Mobility Mass Spectrometry Research Group, Institute of Chemistry, ELTE Eötvös Loránd University, H-1117 Budapest, Hungary; ^4^Department of Immunomedicine, University of Regensburg, D-93053 Regensburg, Germany; ^5^Department of Otorhinolaryngology, University Hospital Regensburg, D-93053 Regensburg, Germany;^6^Institute of Molecular Life Sciences, HUN-REN Research Centre for Natural Sciences, H-1117 Budapest, Hungary; ^7^Center for Cancer Research, Medical University of Vienna, 1090 Vienna, Austria; ^8^Department of Metabolism, Digestion and Reproduction, Imperial College London, SW7 2AZ London, United Kingdom


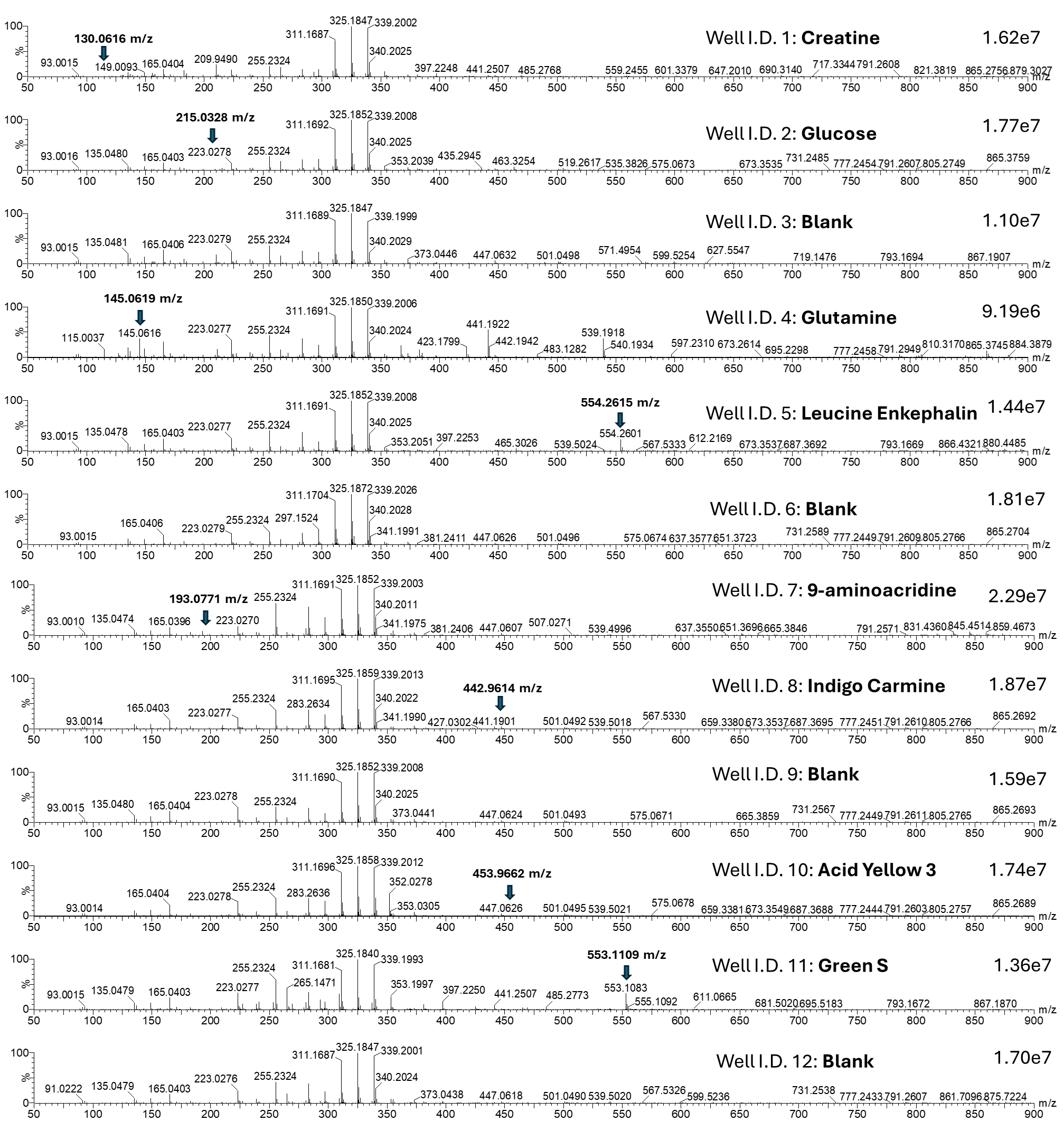


Fig. S1. Laser Ablation - Rapid Evaporative Ionization Mass Spectrometry spectra for each compound of the high-throughput and carryover assessment for creatine, glucose, glutamine, leucine-enkephalin, 9-aminoacridine, indigo carmine, acid yellow 3 and green S with 4 additional blanks (Negative ion mode). The spectra are combined from 5 consecutive scans, lock massed on palmitic acid (255.2324 m/z) and are showcased from 50 – 900 m/z.


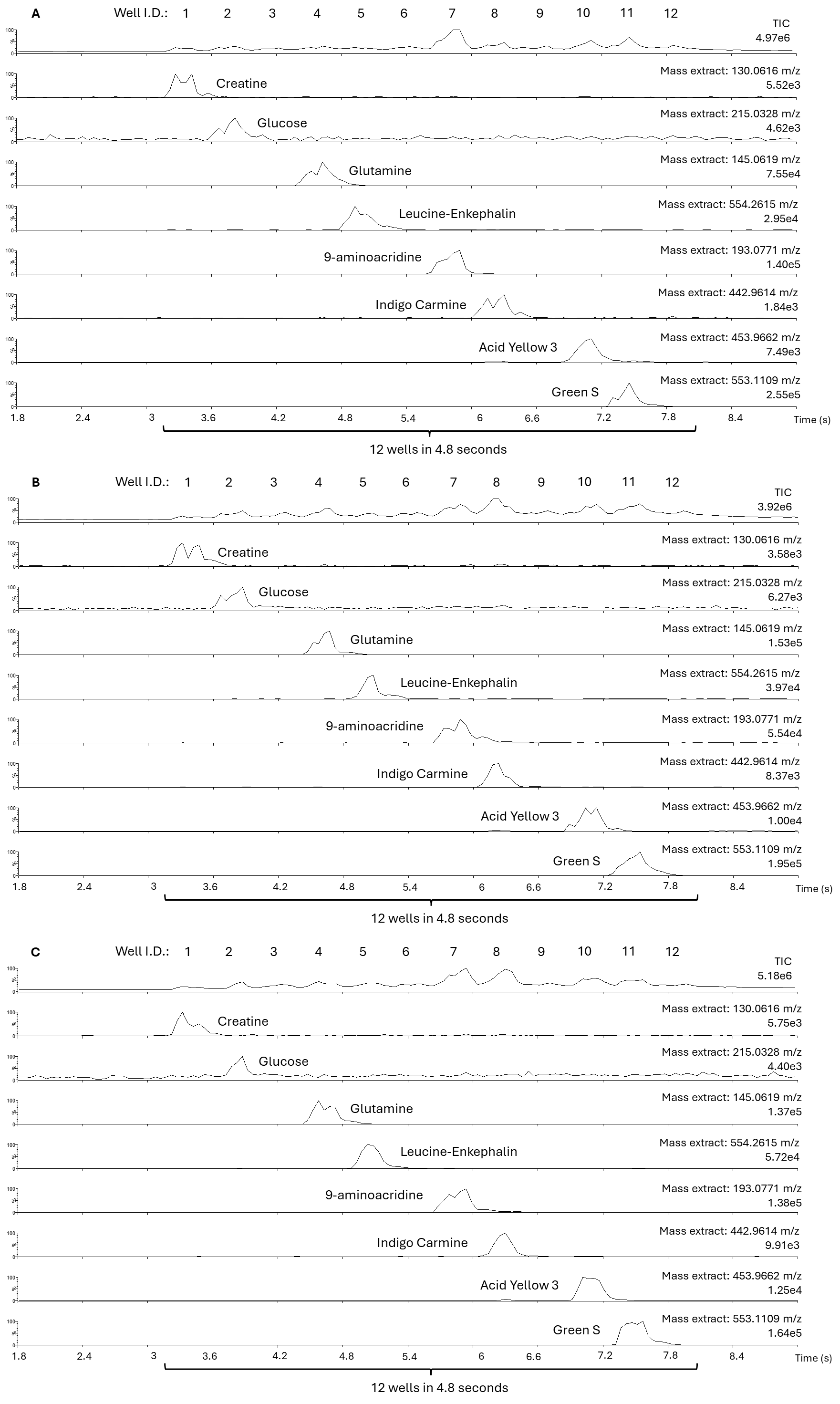


Fig. S2. Triplicate mass spectrometry analysis via AmbiSampler of compound set containing creatine, glucose, glutamine, leucine-enkephalin, 9-aminoacridine, indigo carmine, acid yellow 3 and green S with 4 additional blanks (A = 1^st^ run; B = 2^nd^ run; C = 3^rd^ run). In each run, 12 wells were measured in 4.8 seconds.


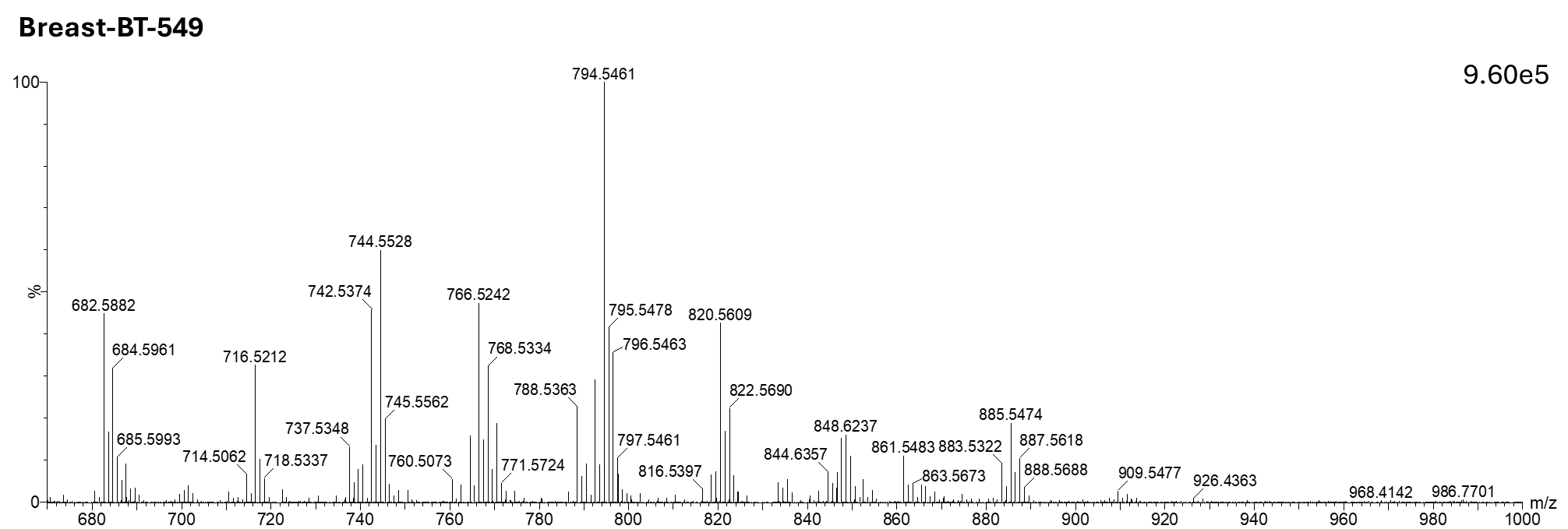


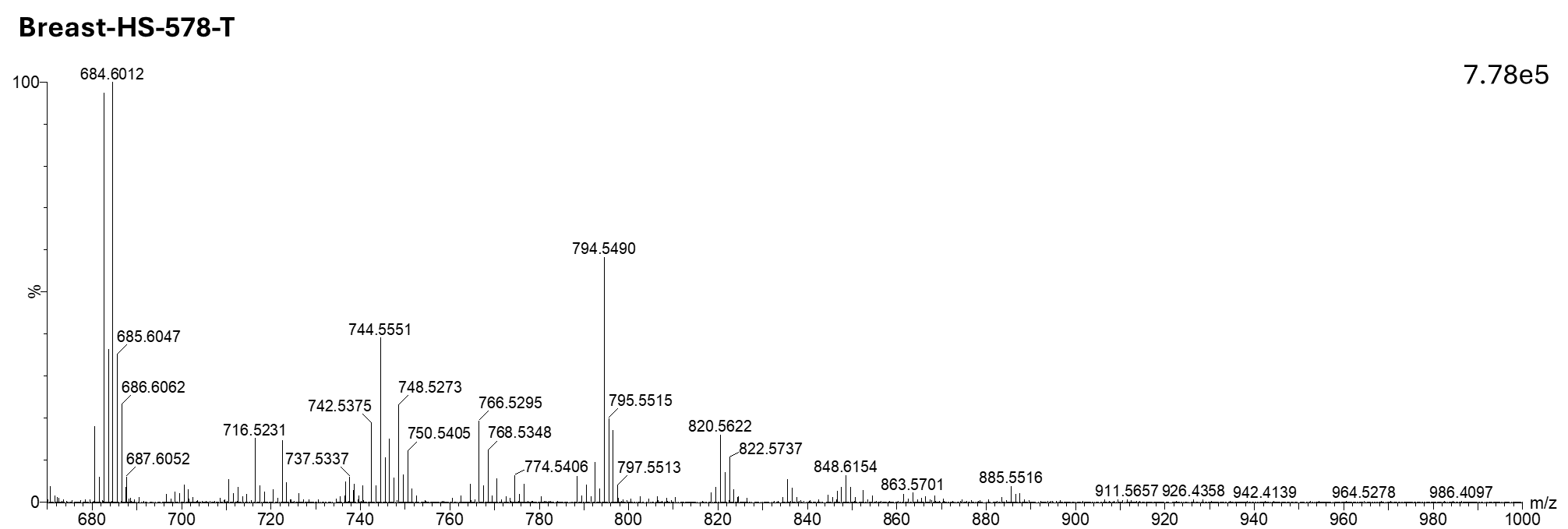


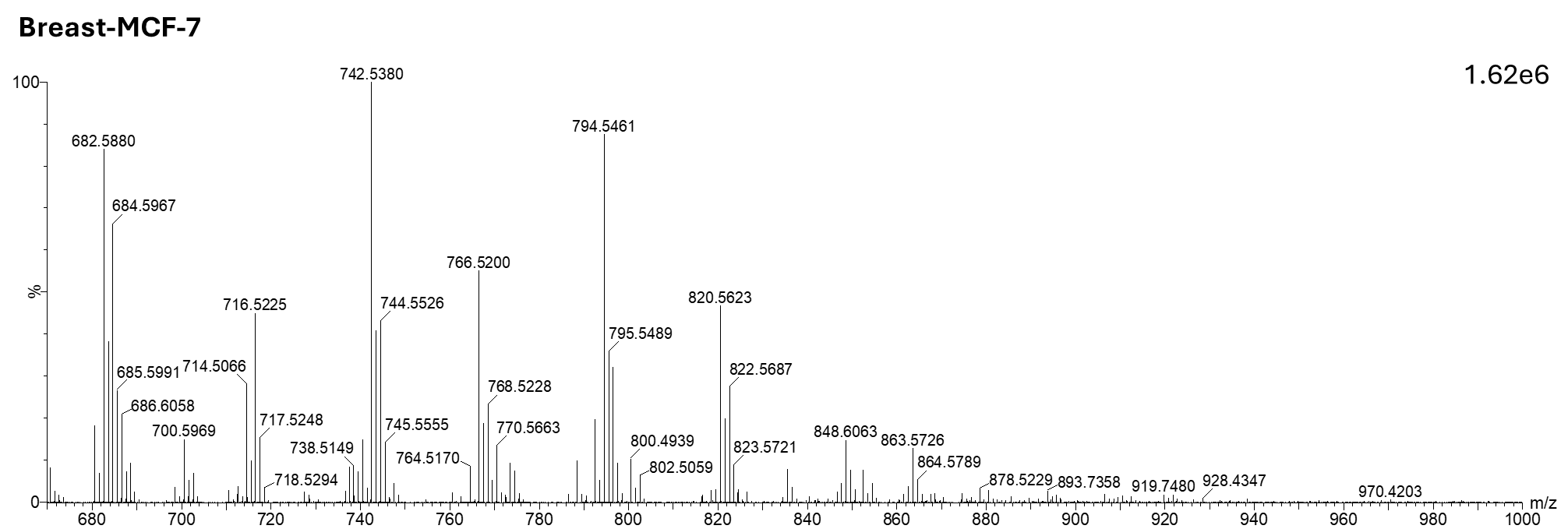


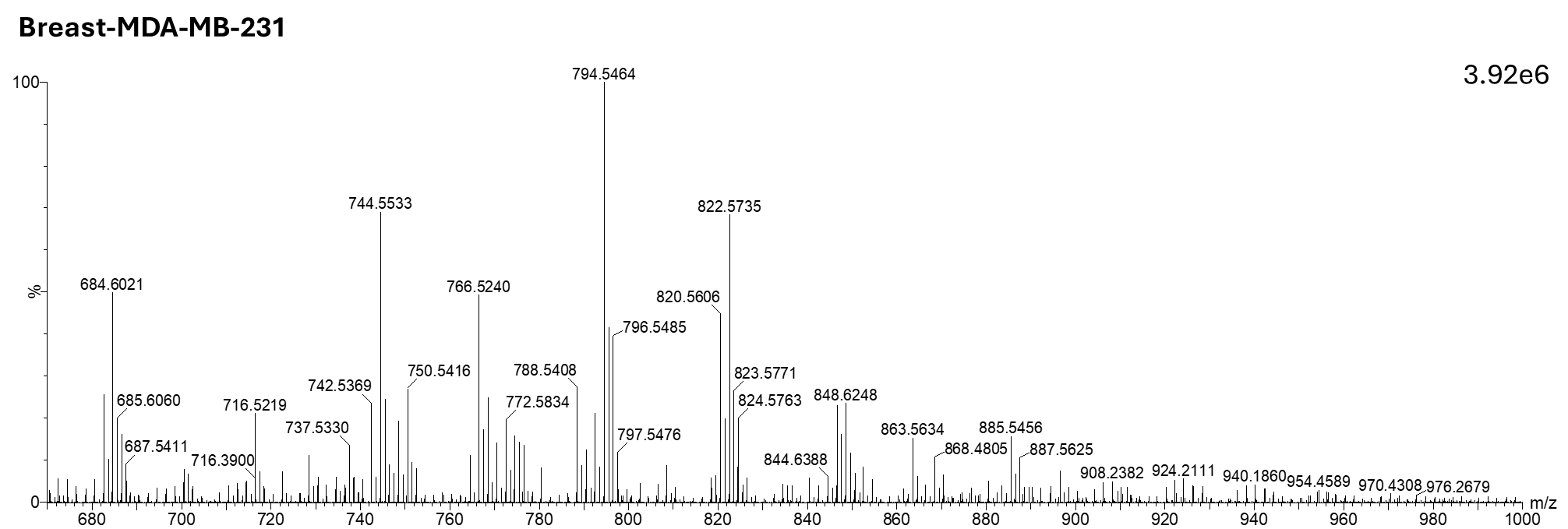


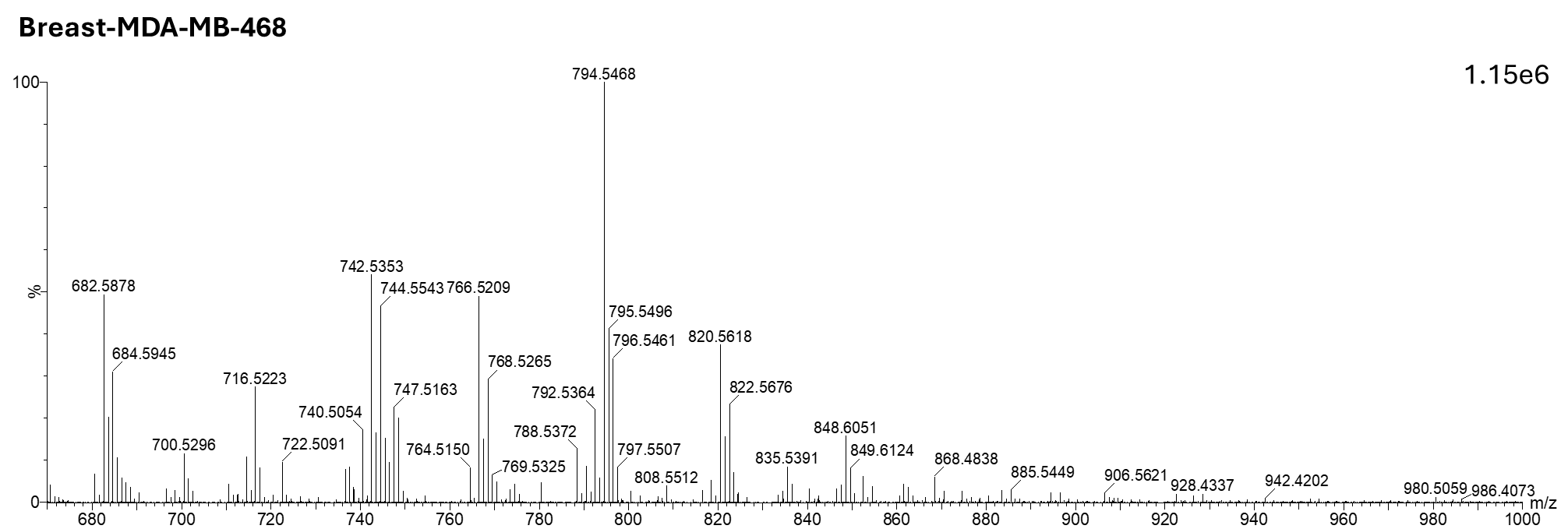


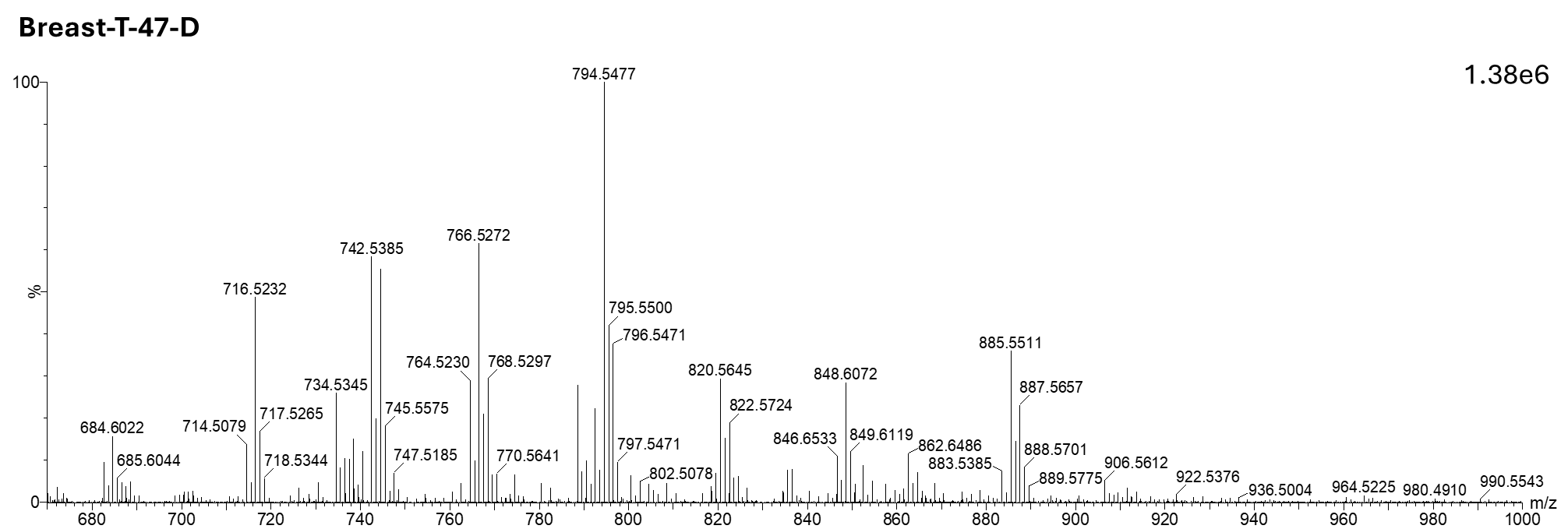


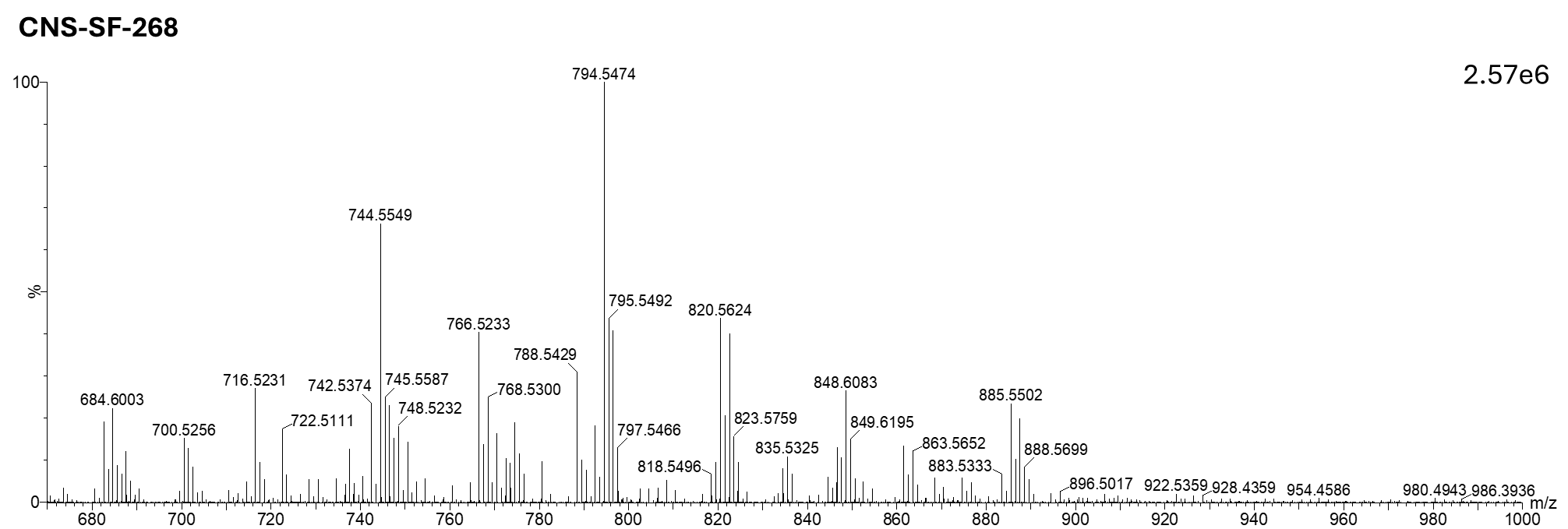


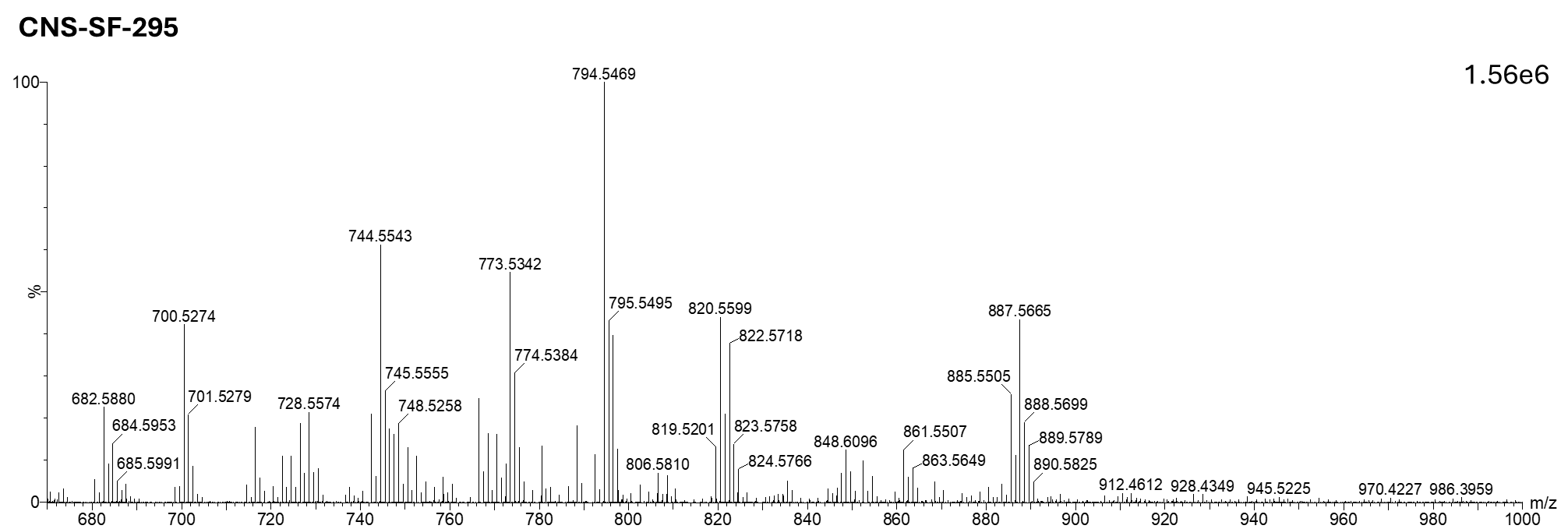


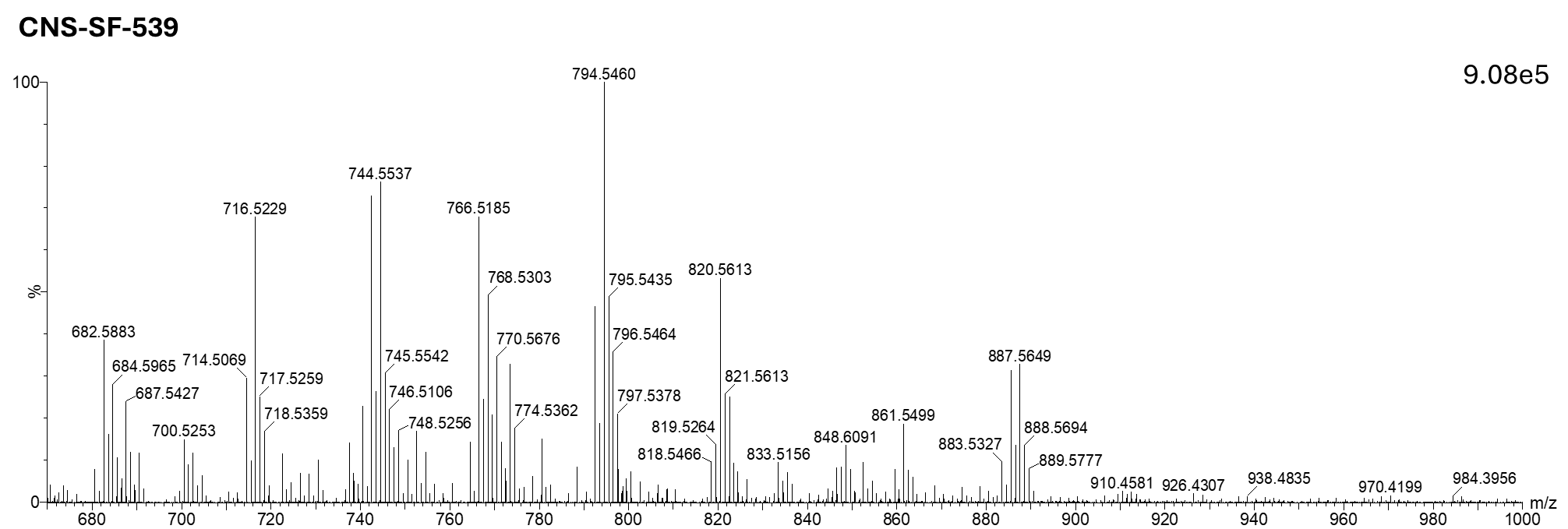


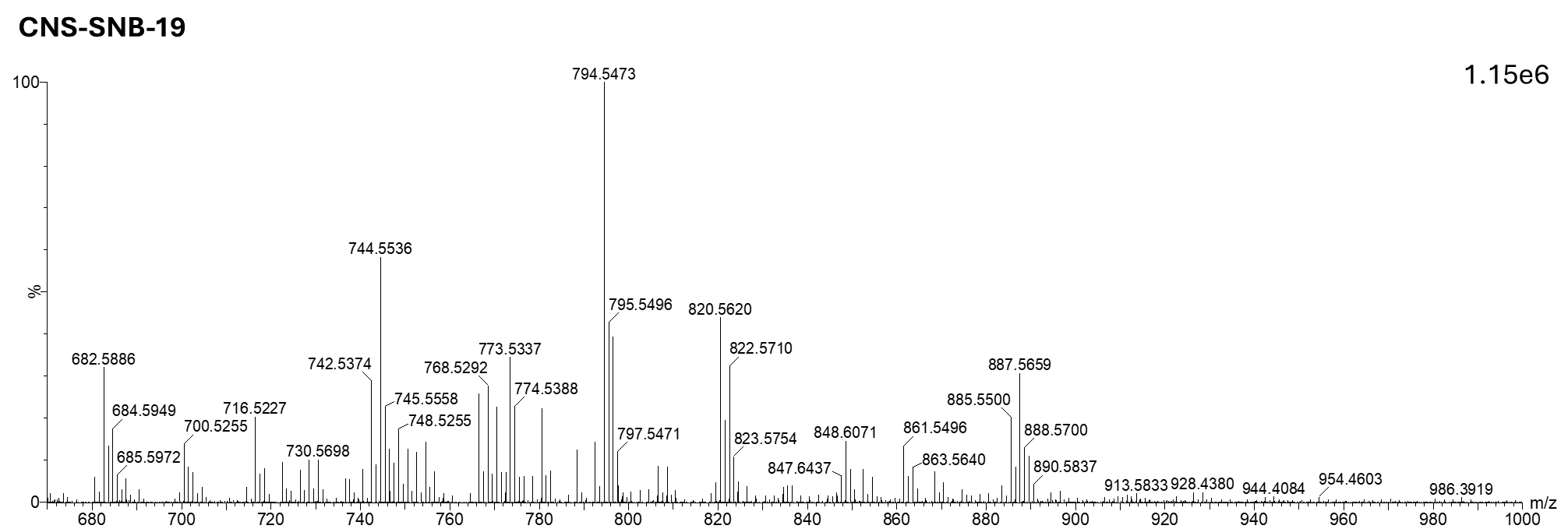


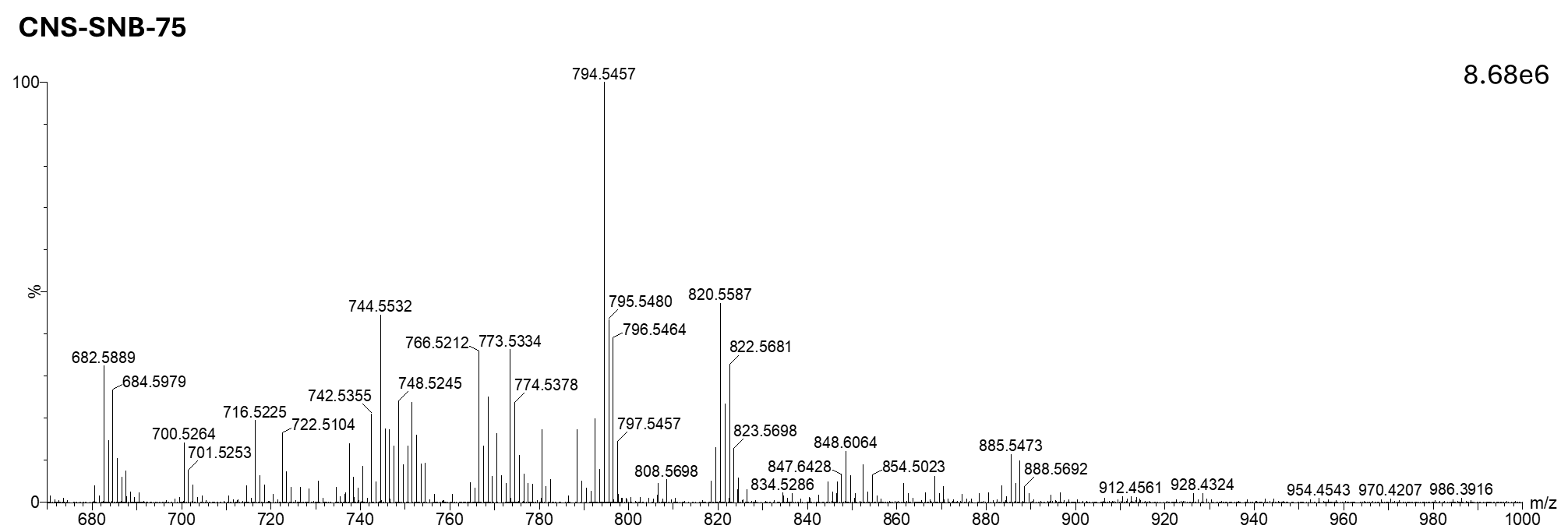


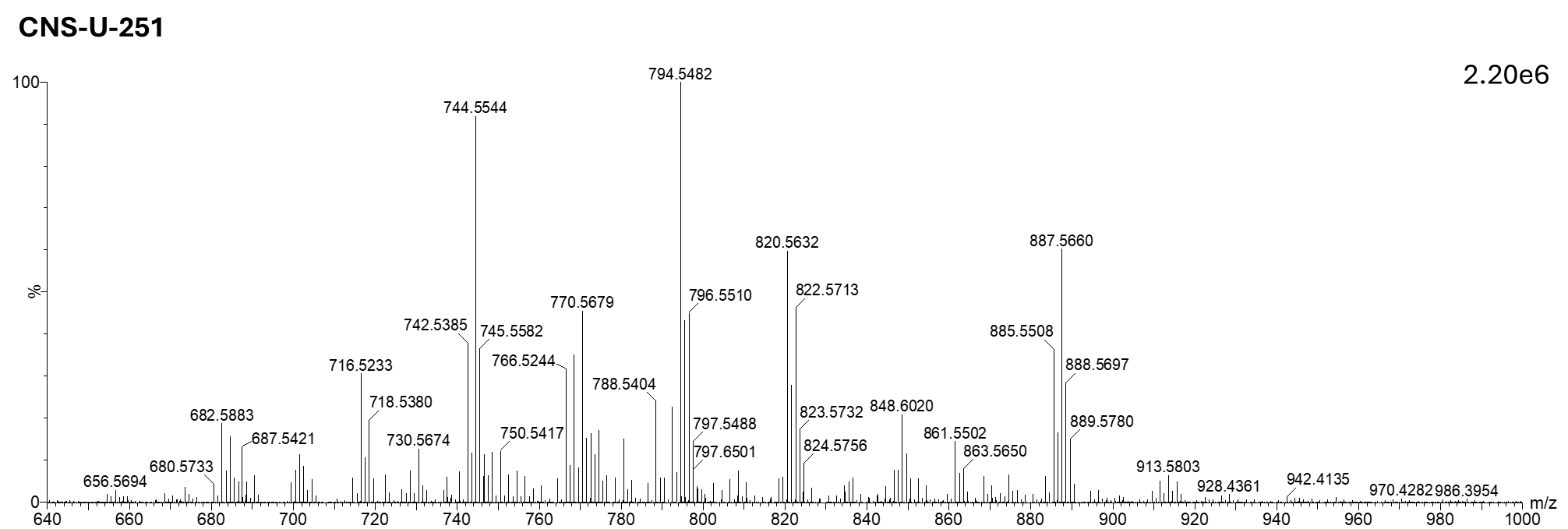


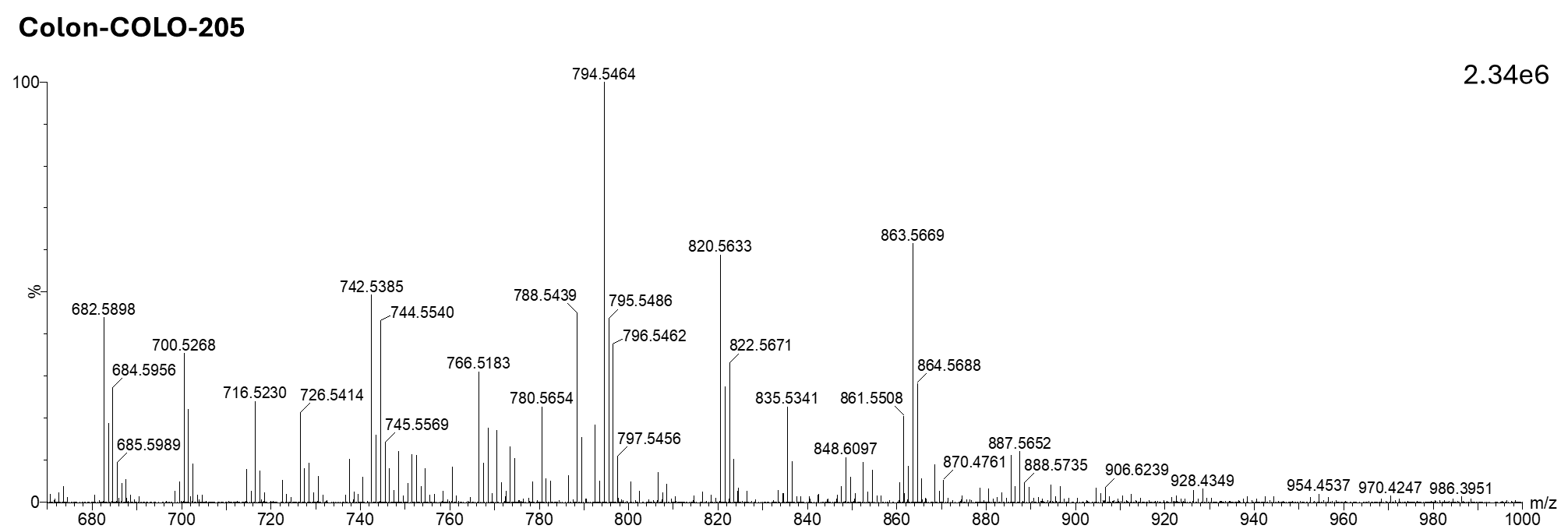


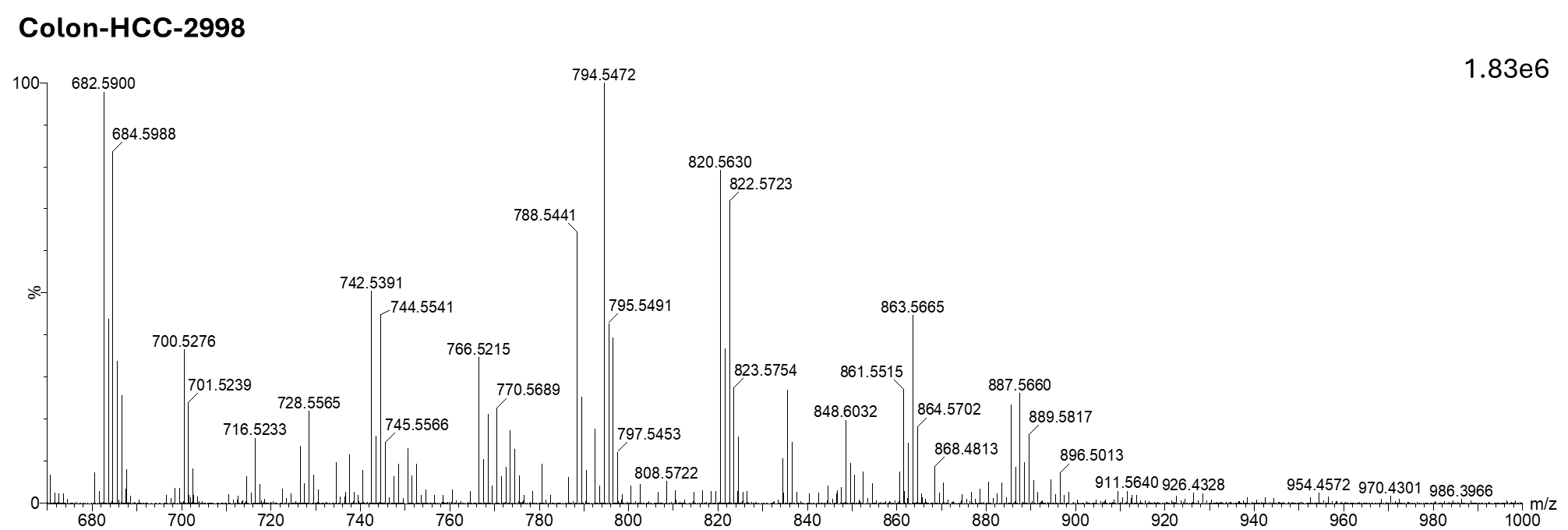


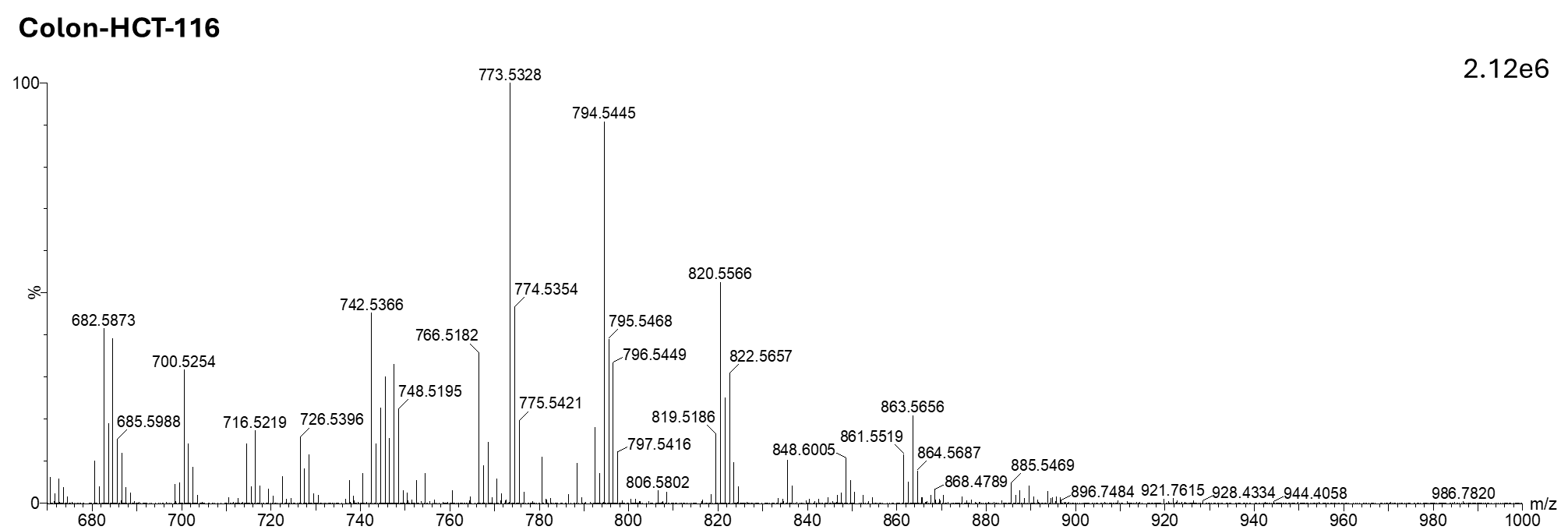


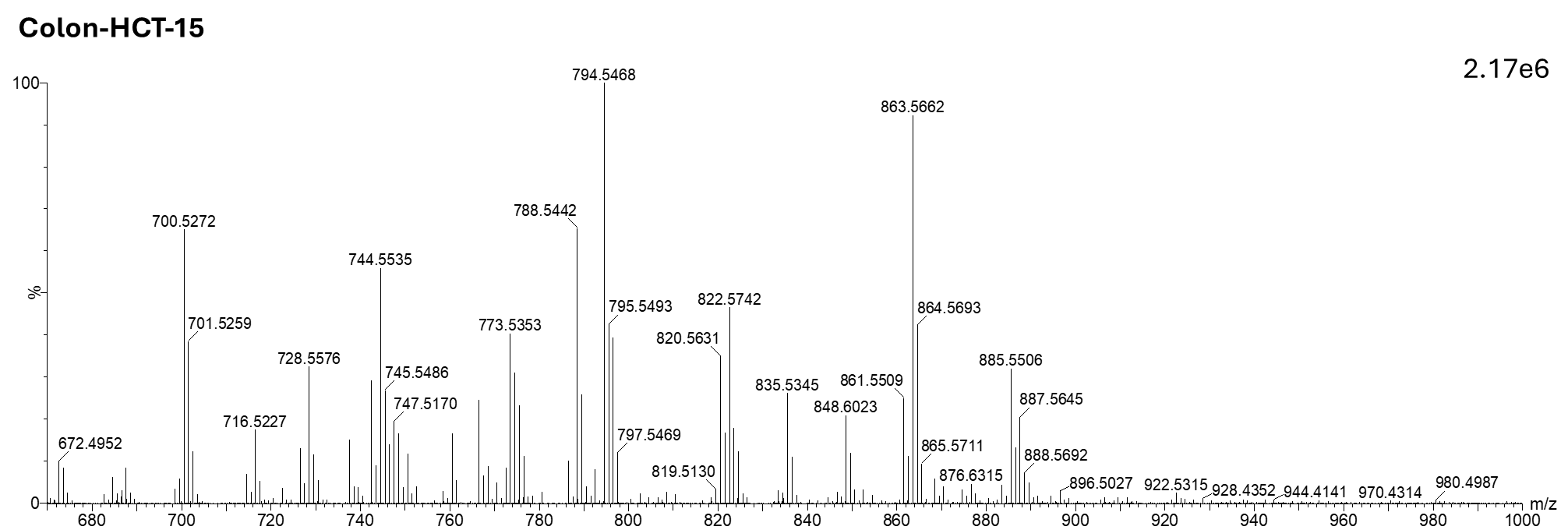


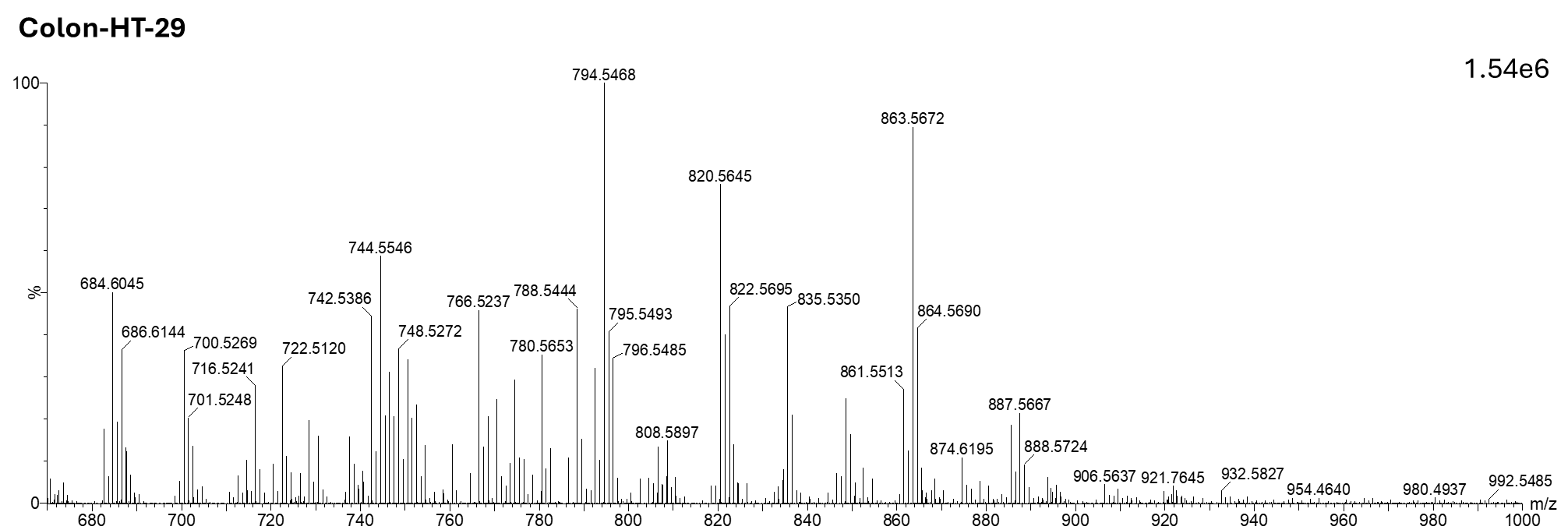


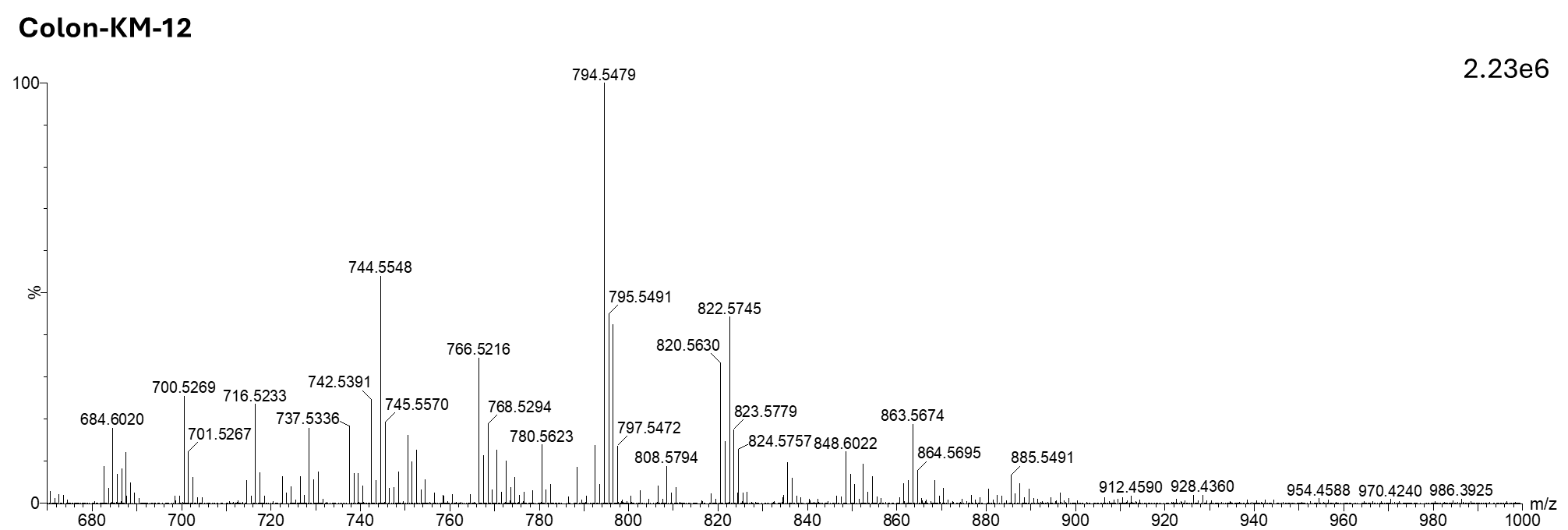


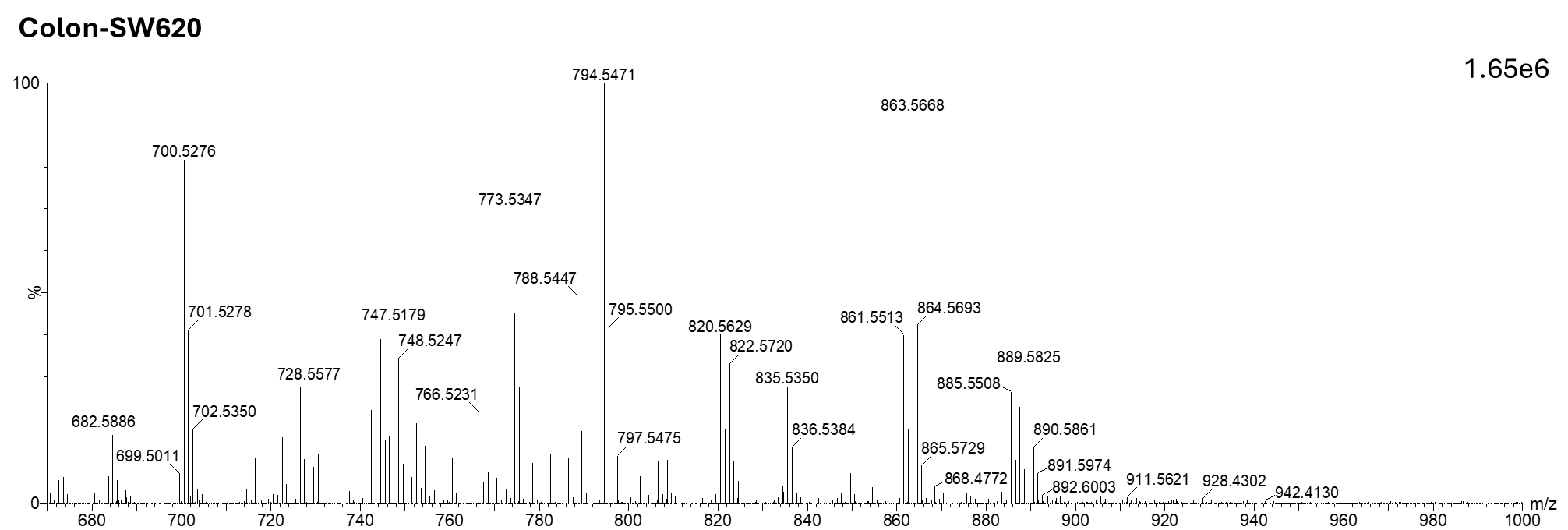


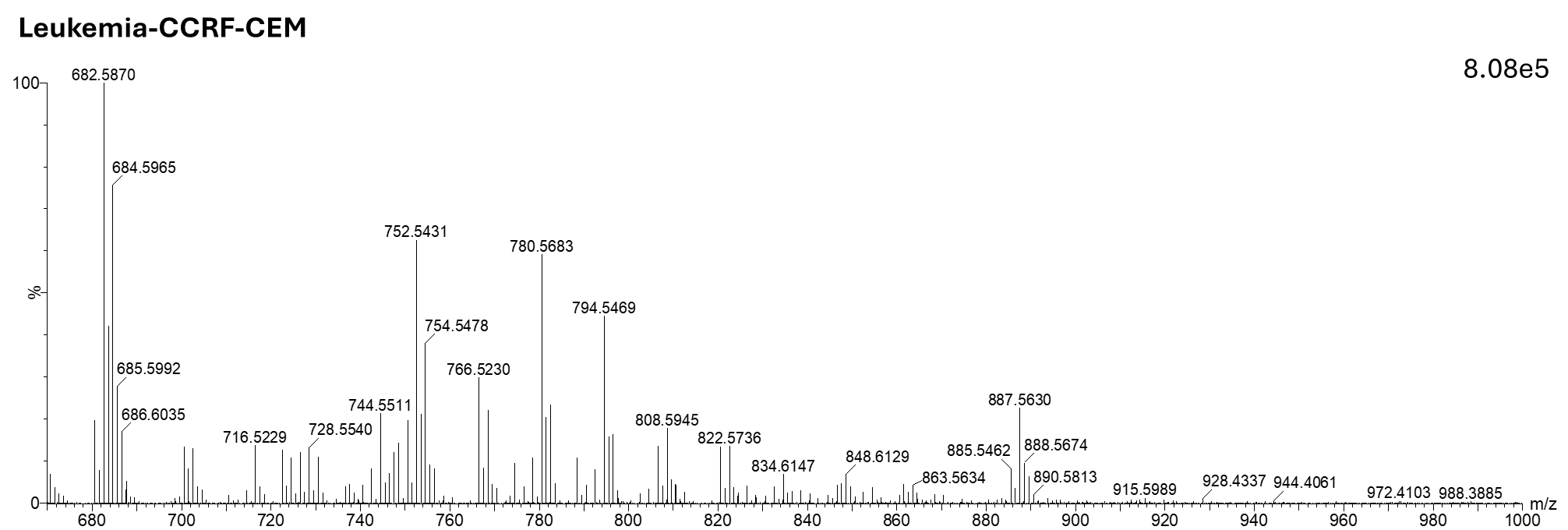


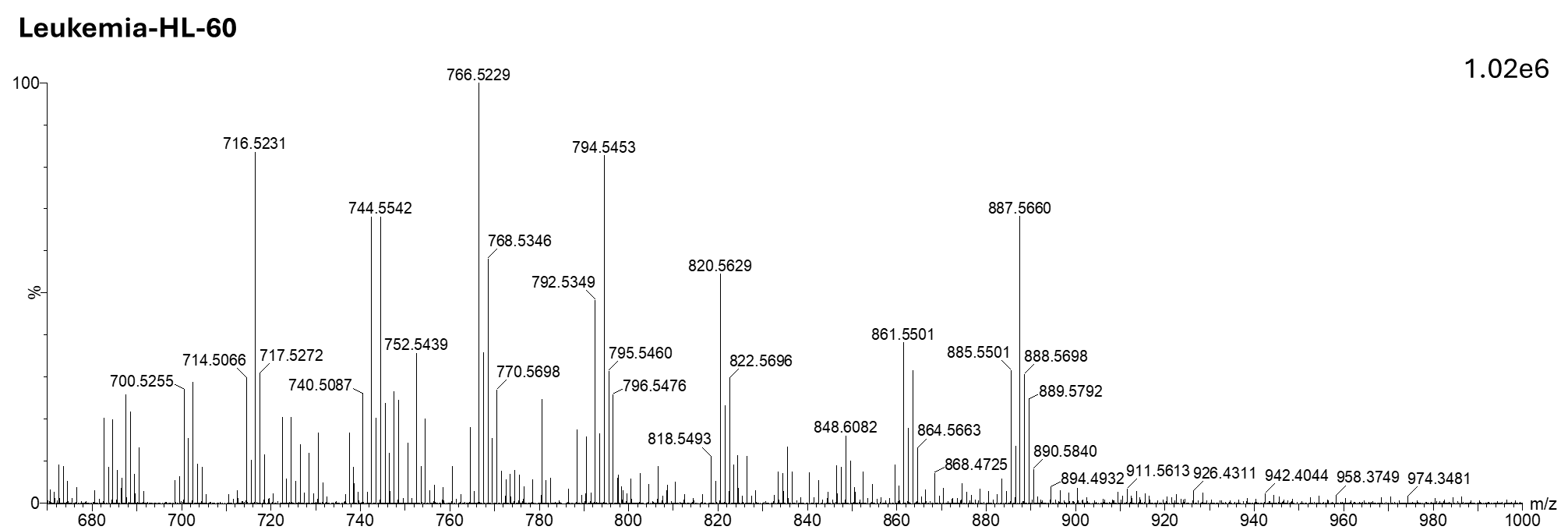


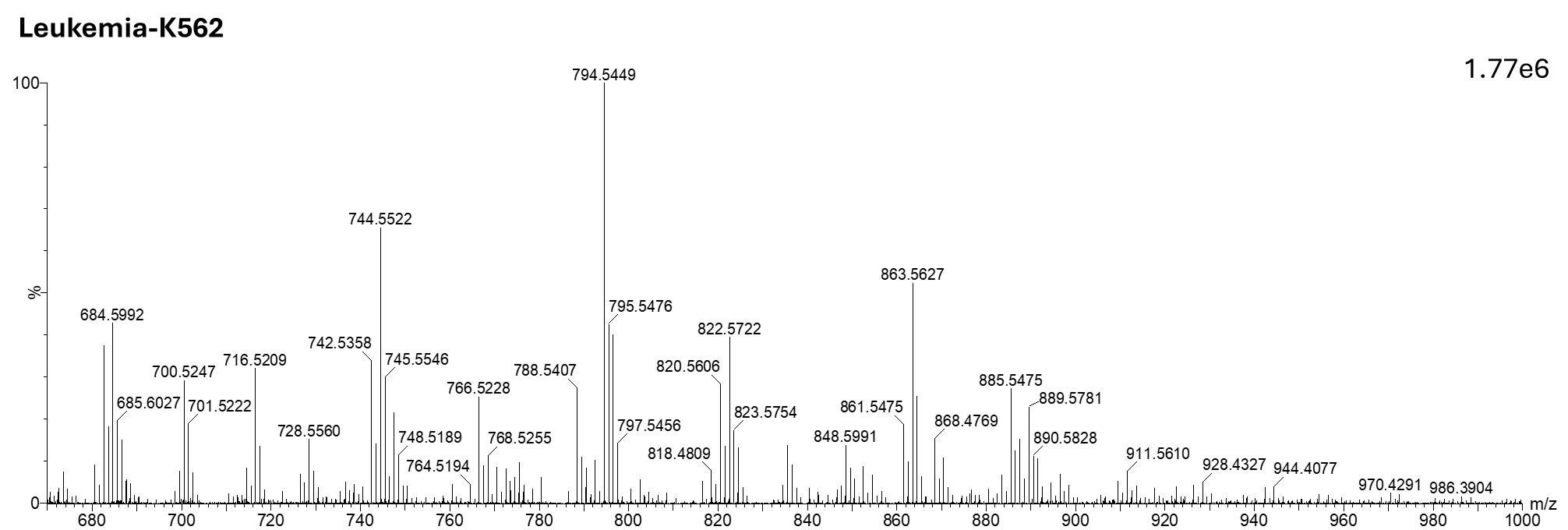


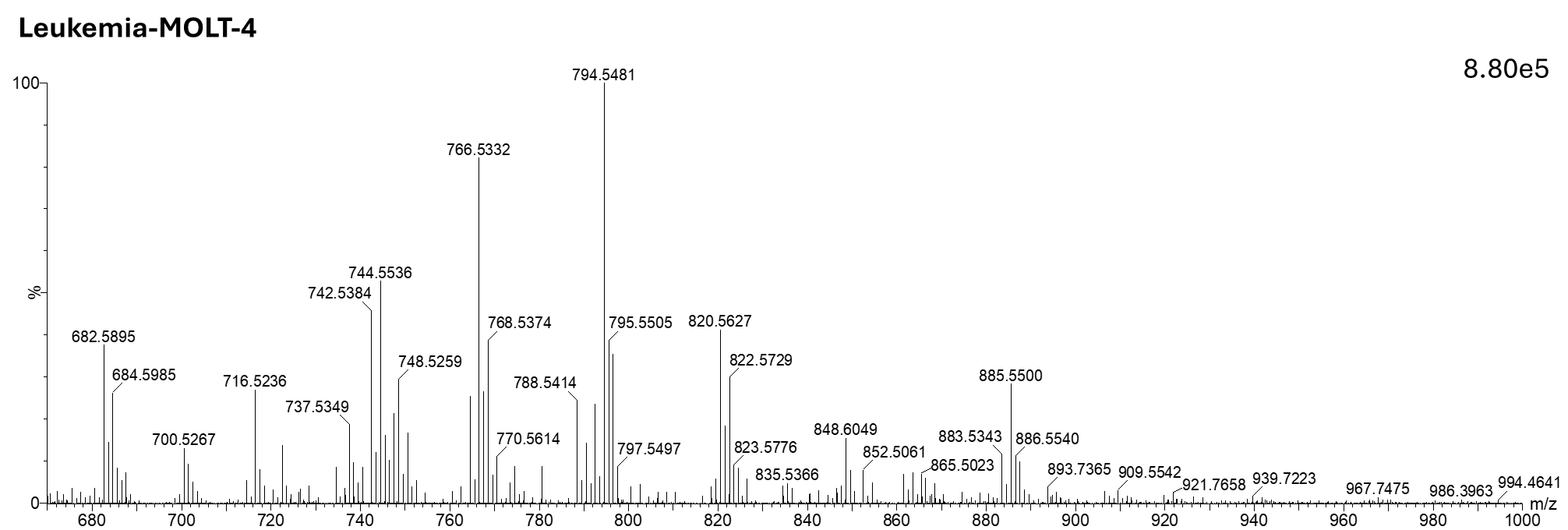


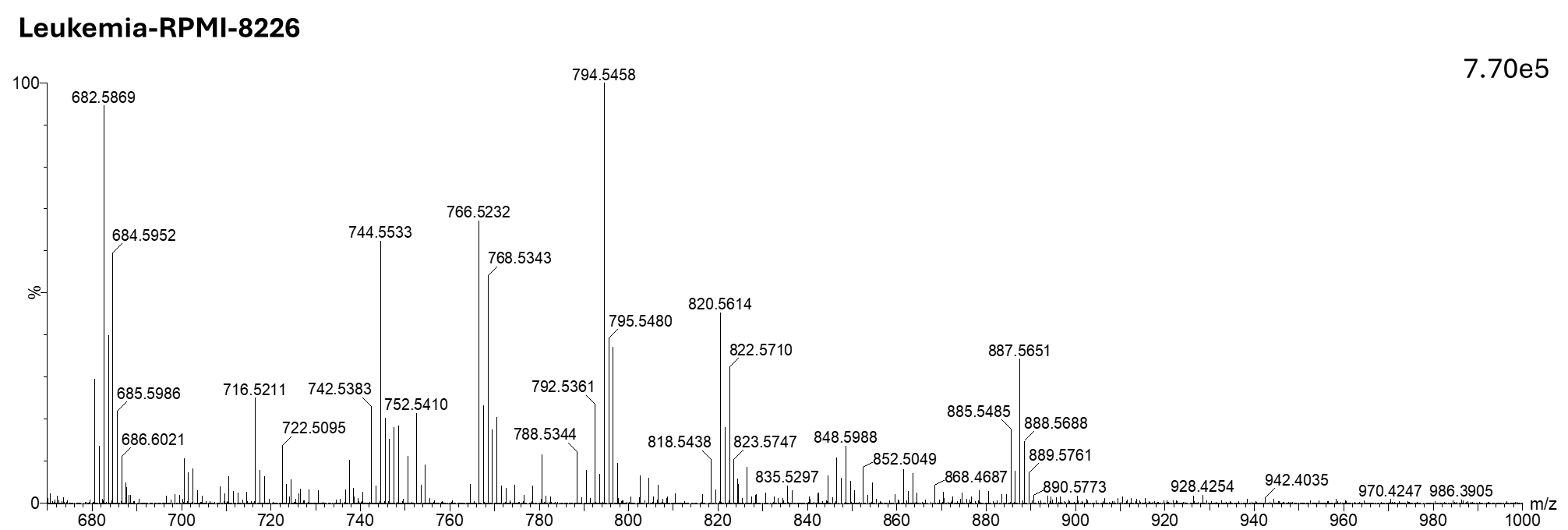


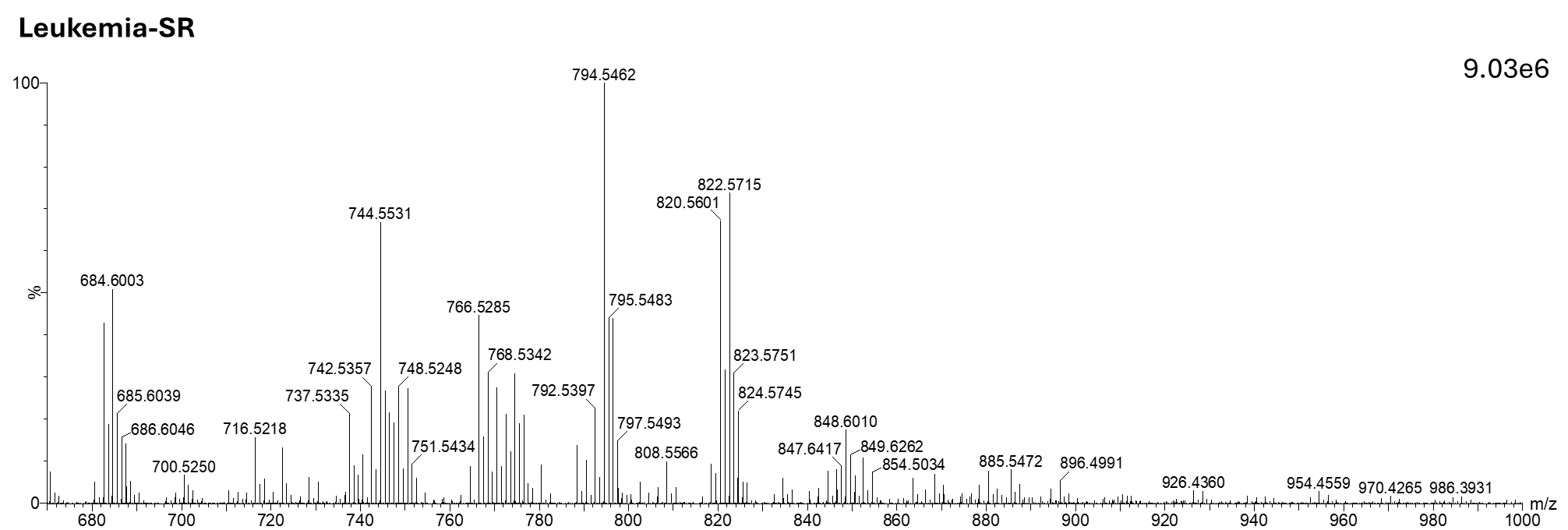


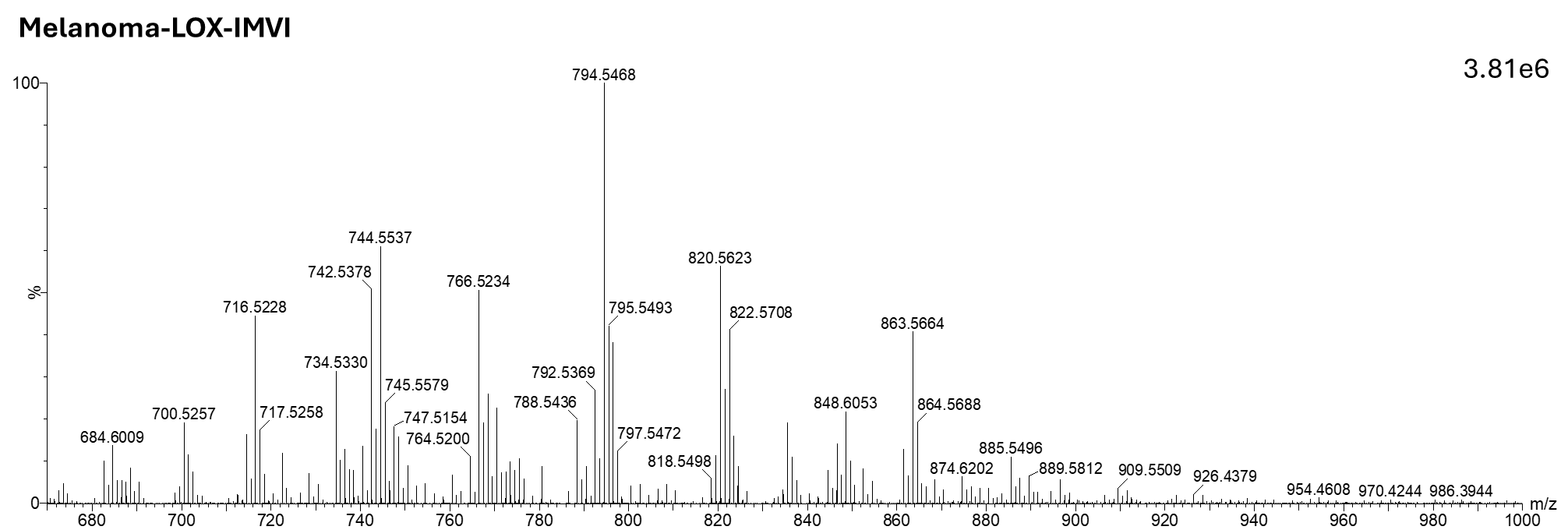


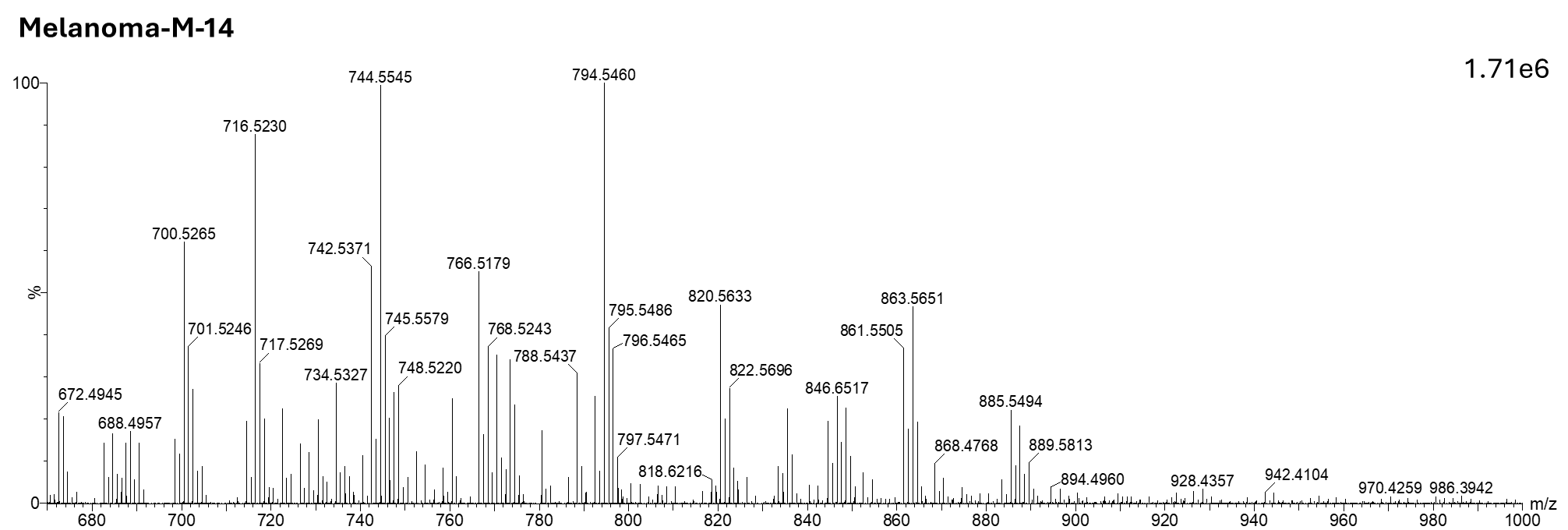


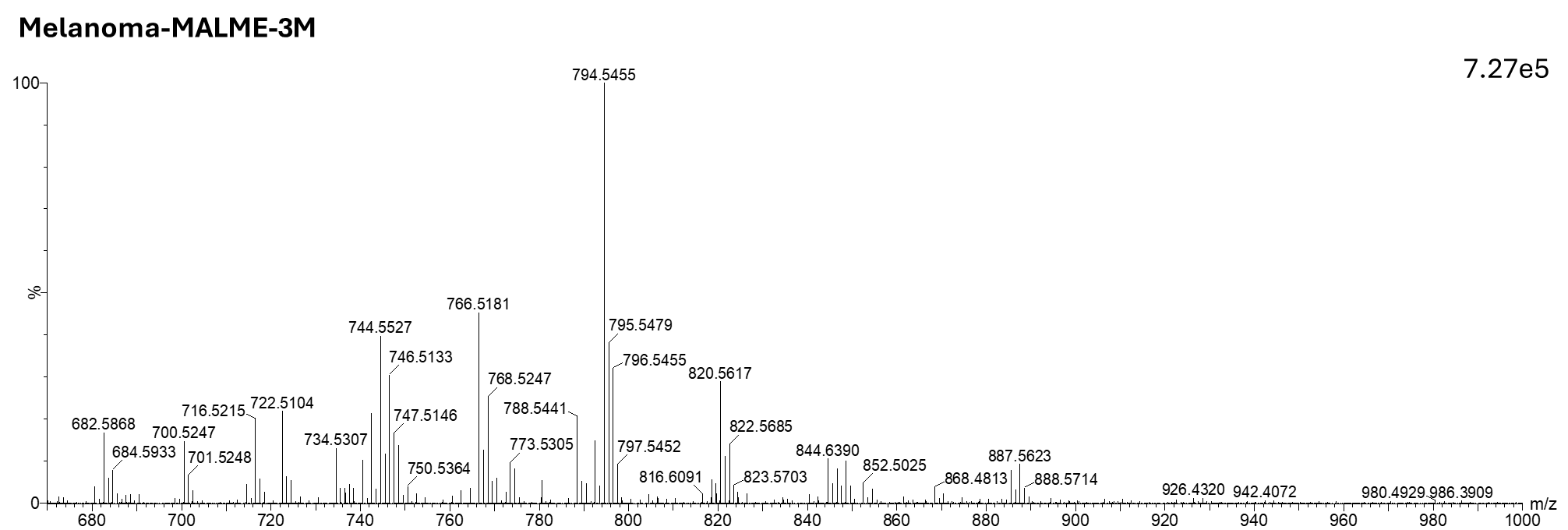


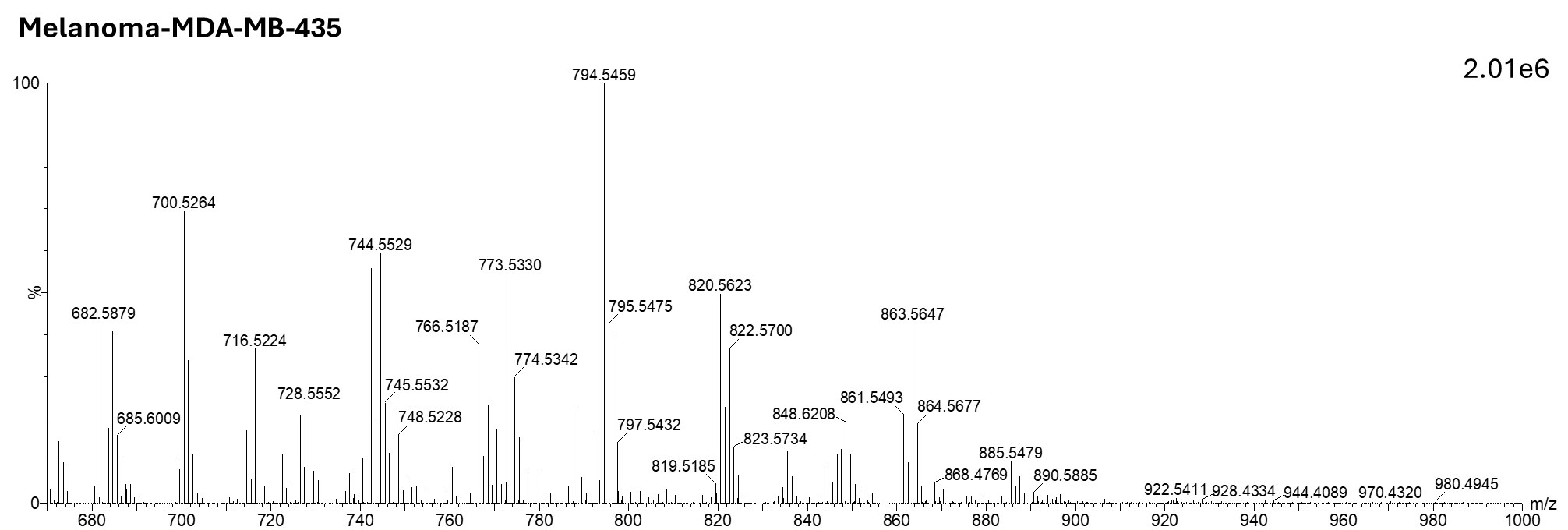


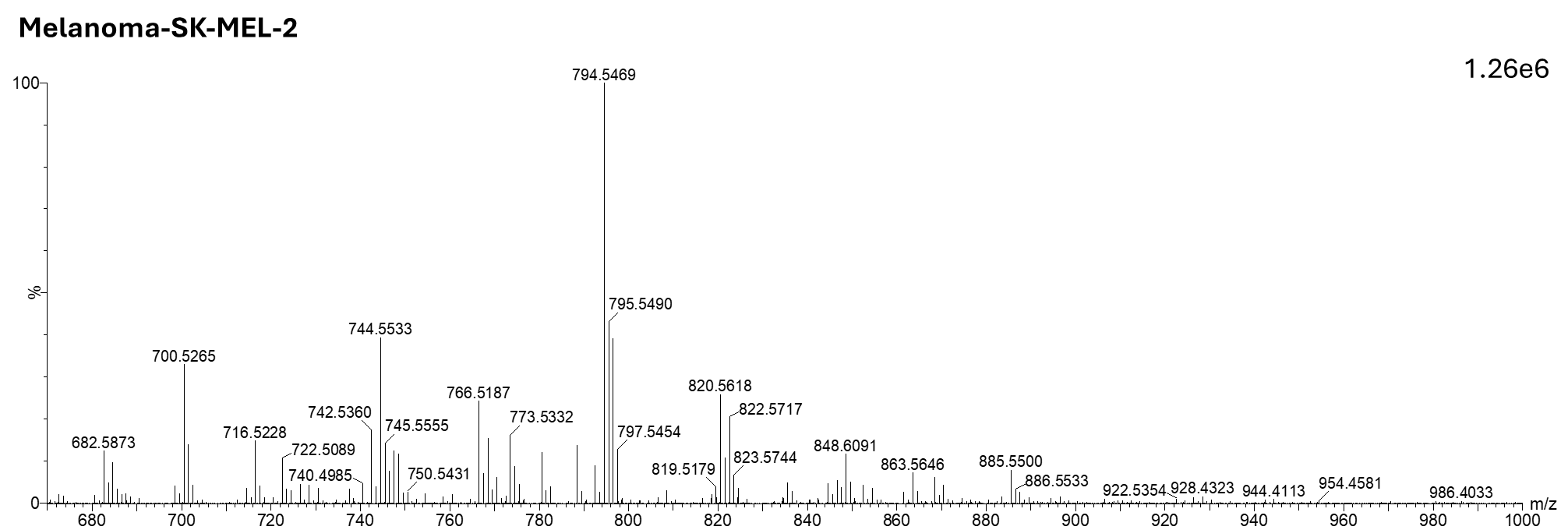


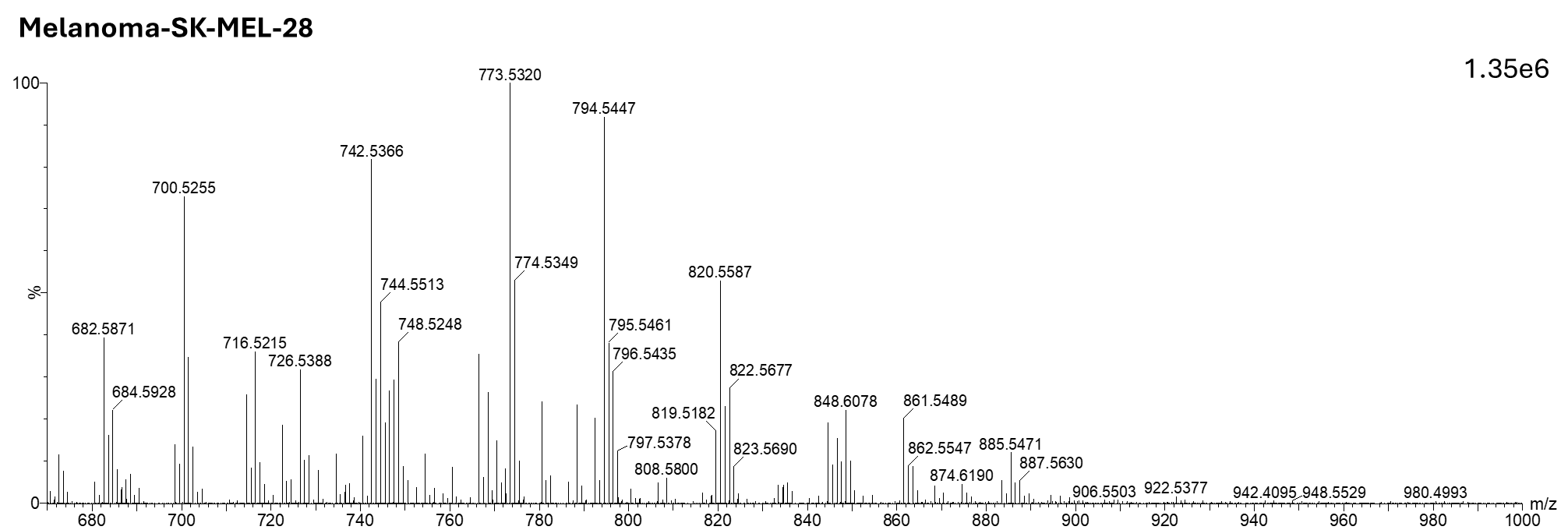


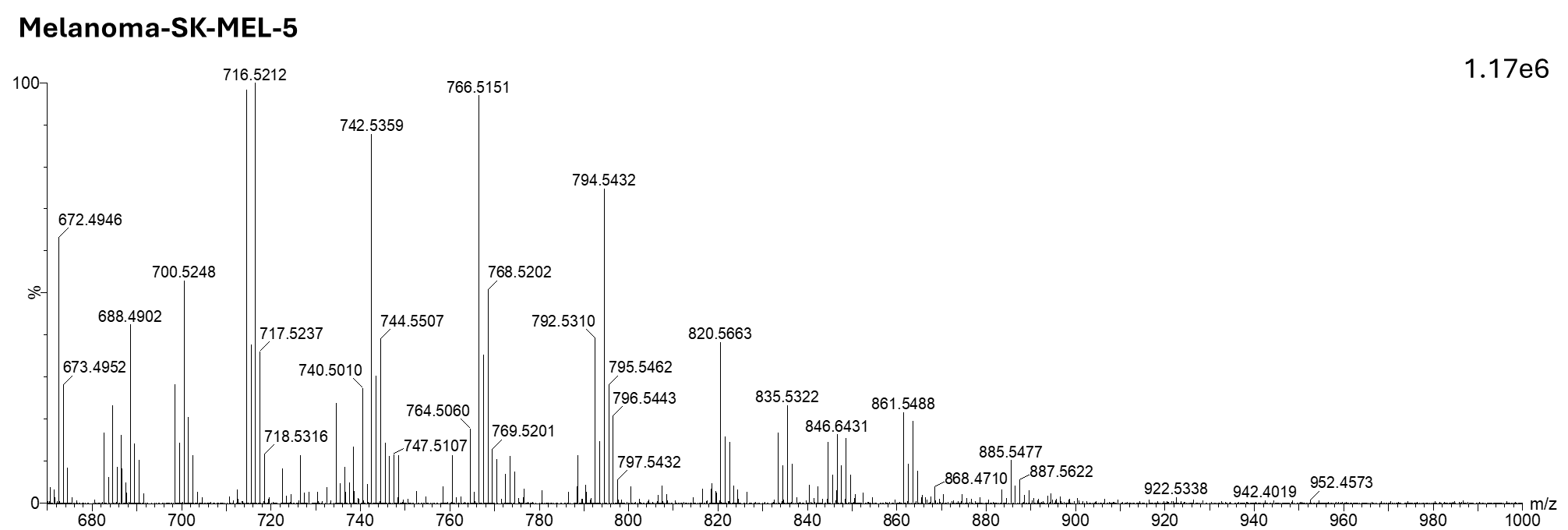


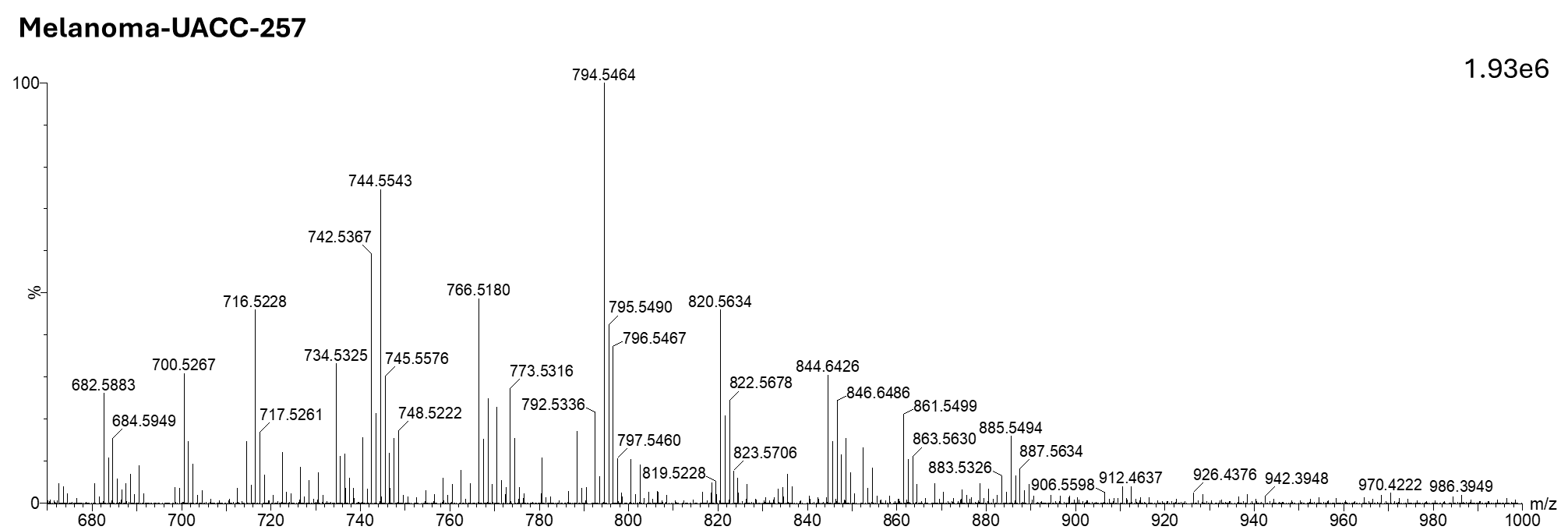


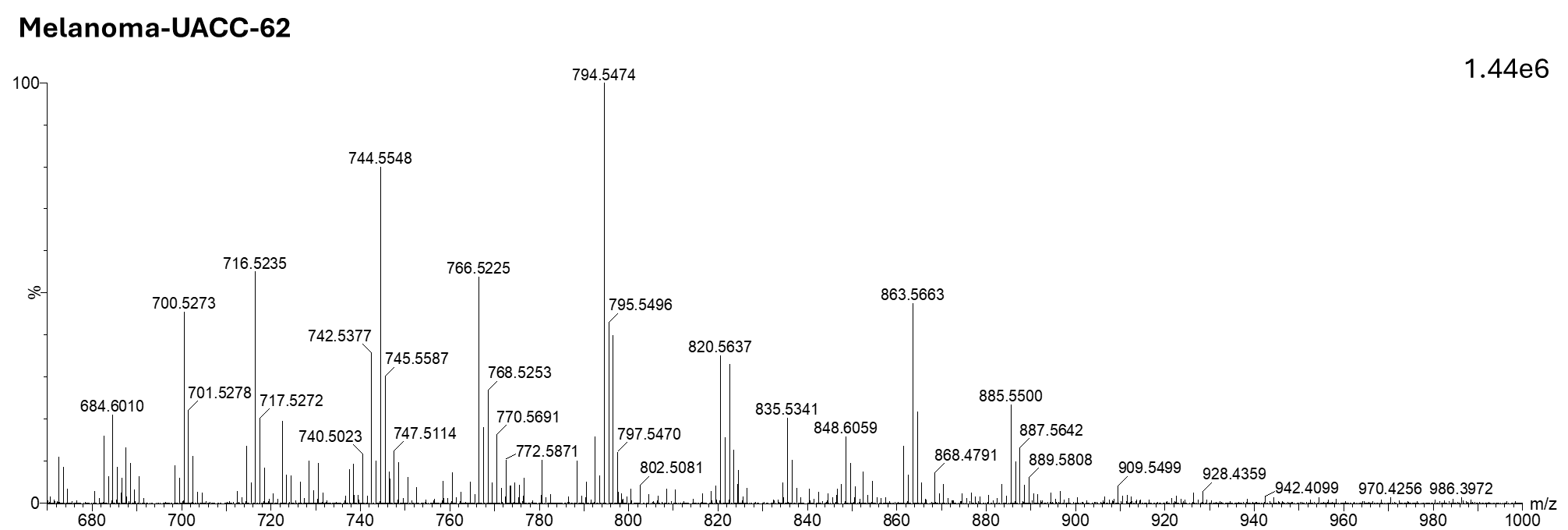


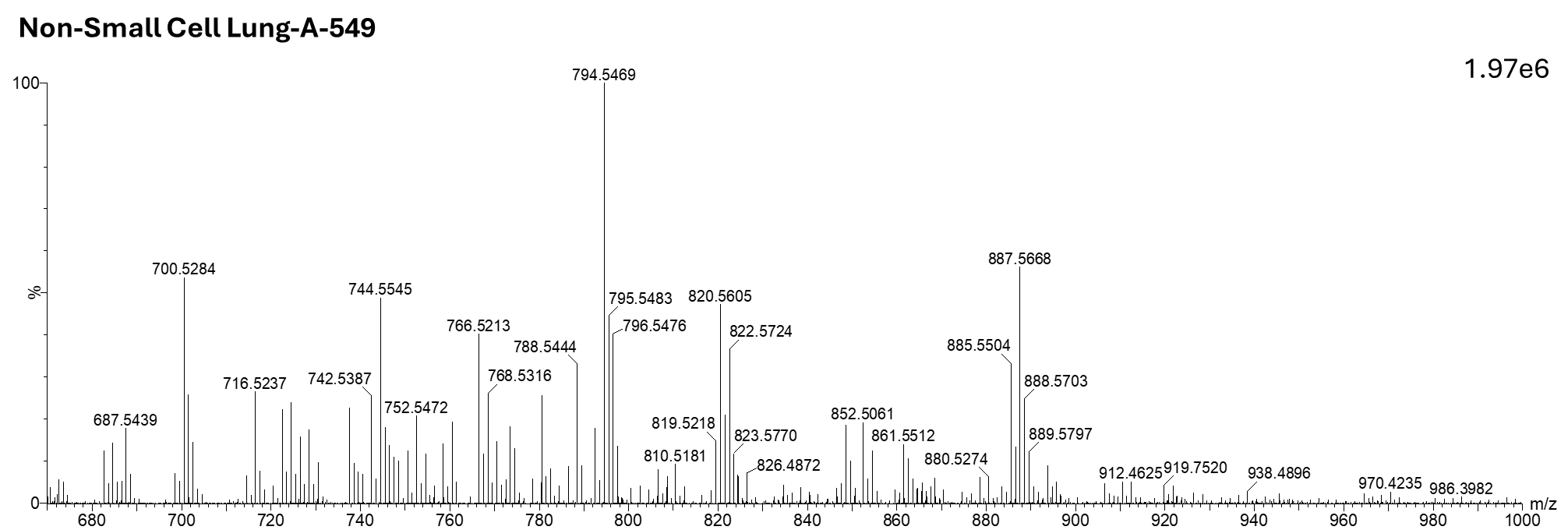


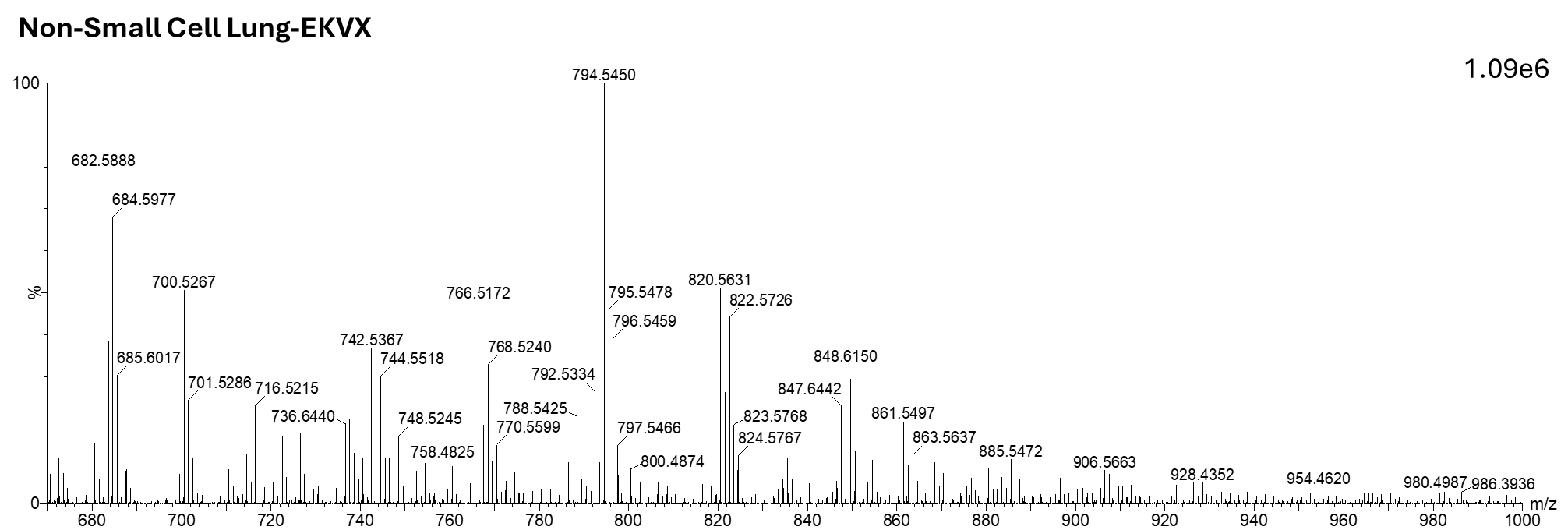


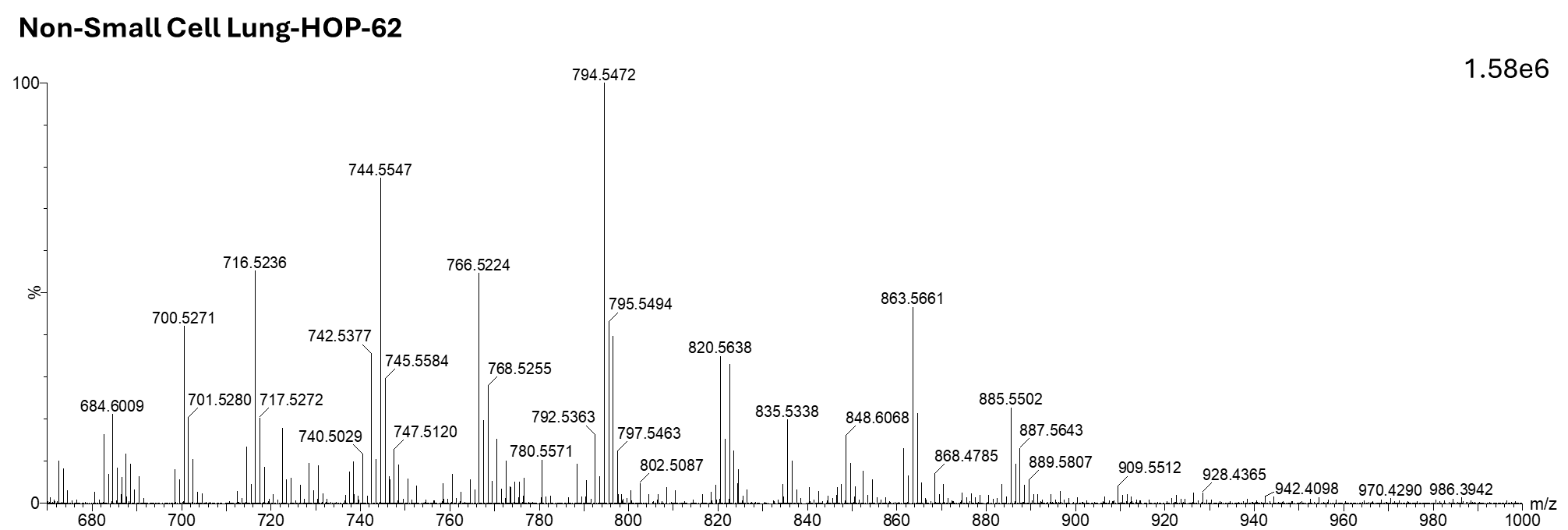


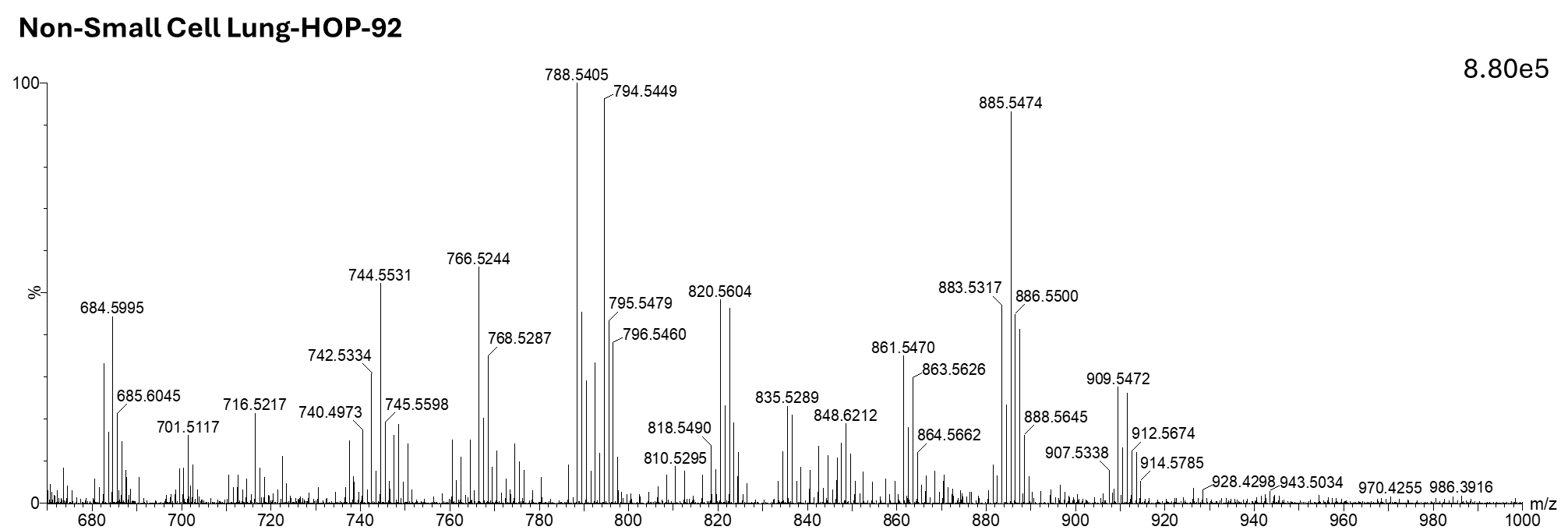


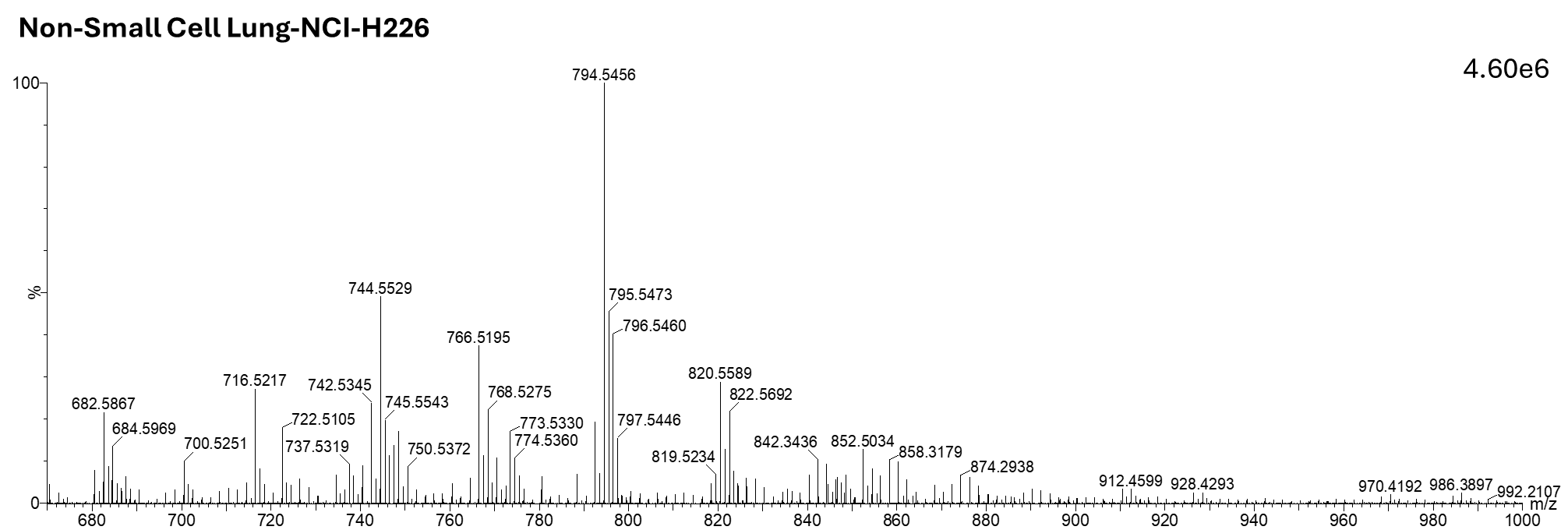


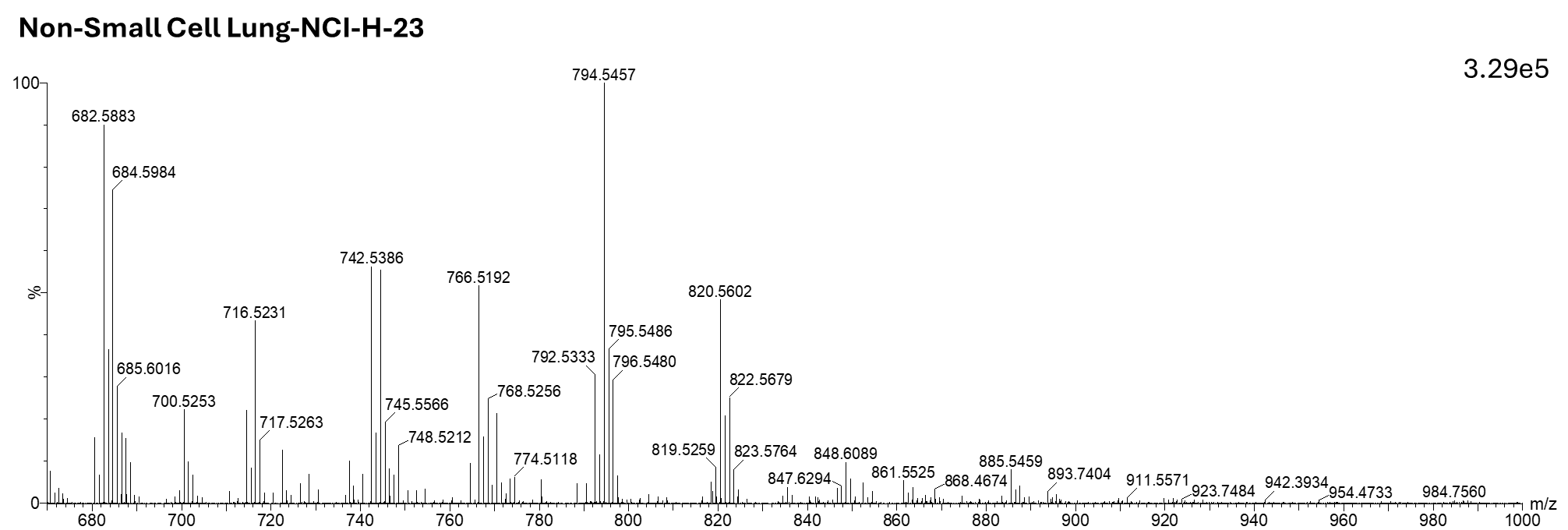


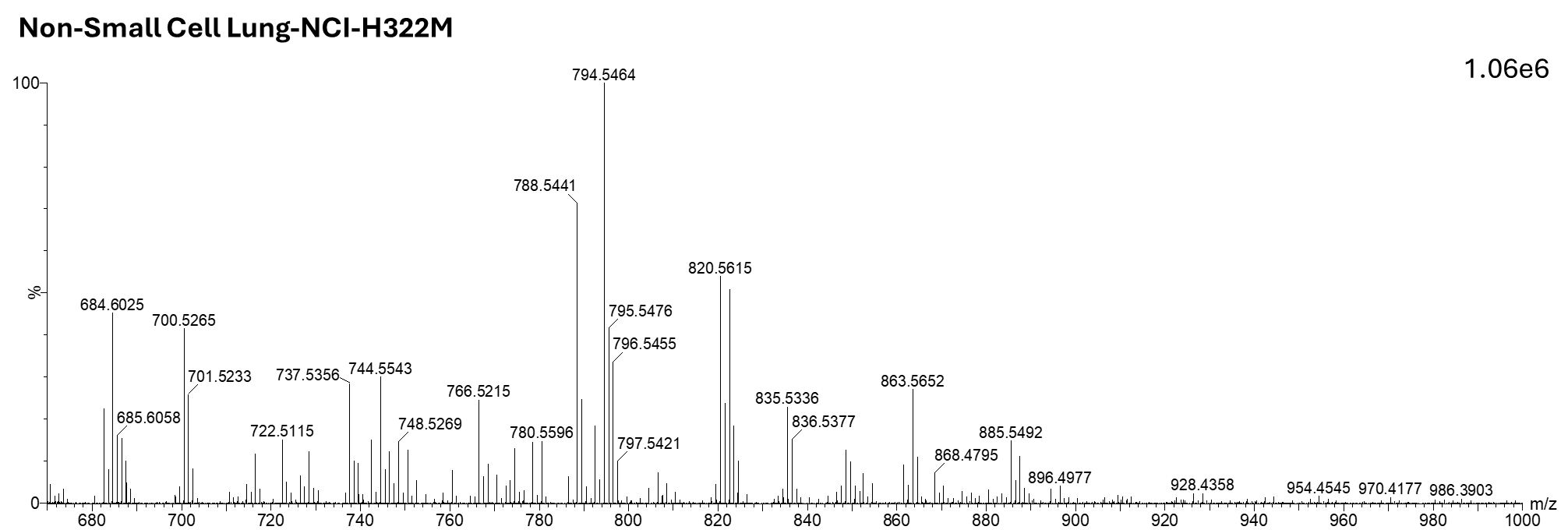


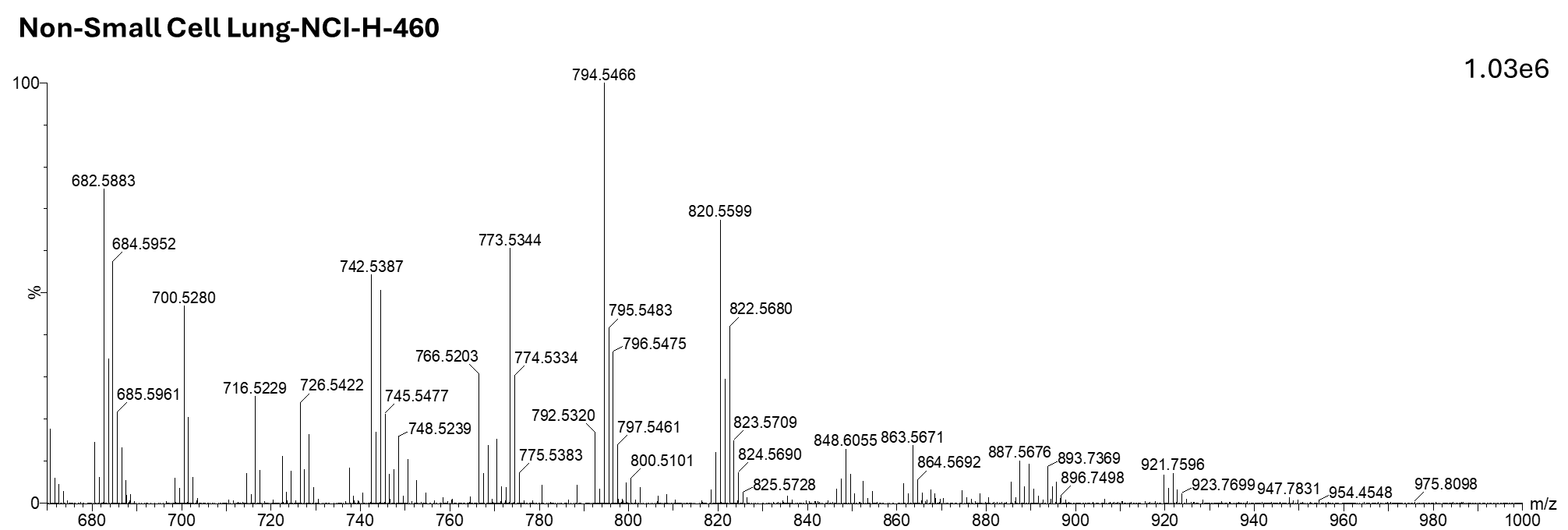


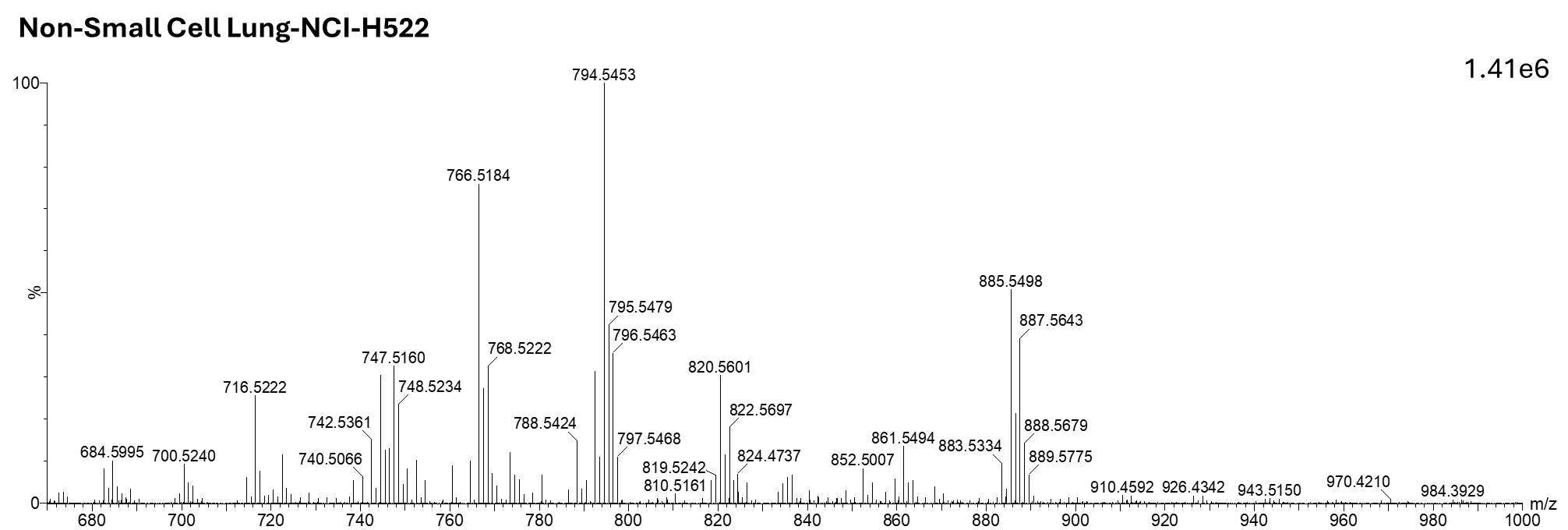


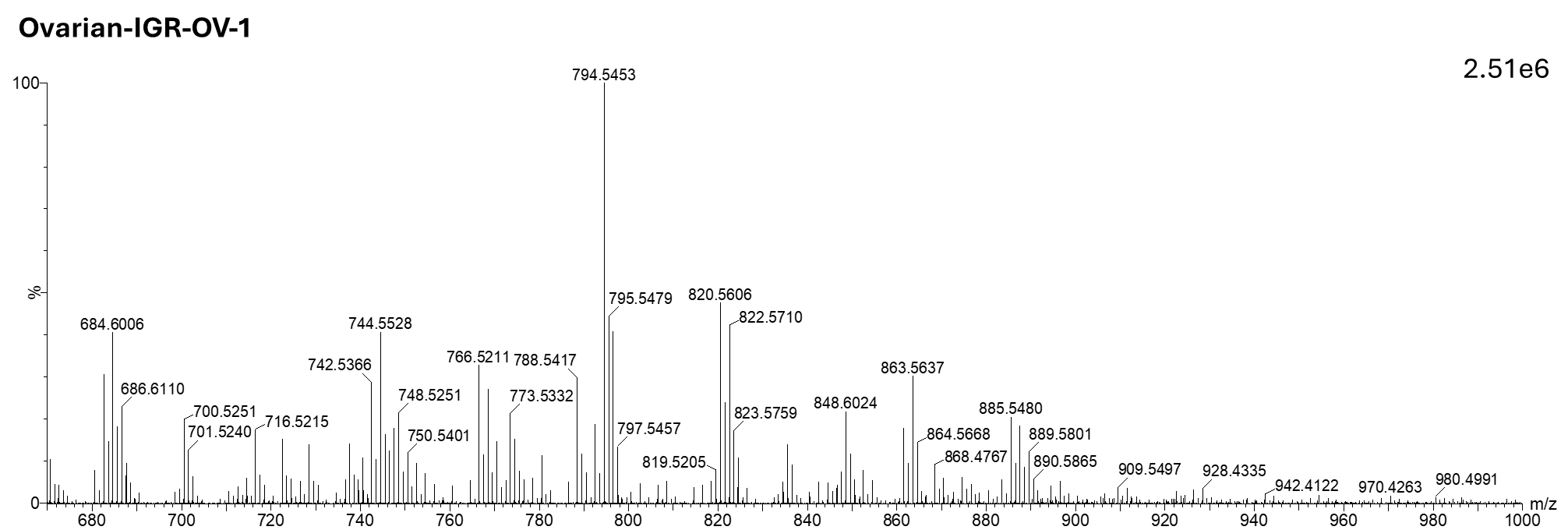


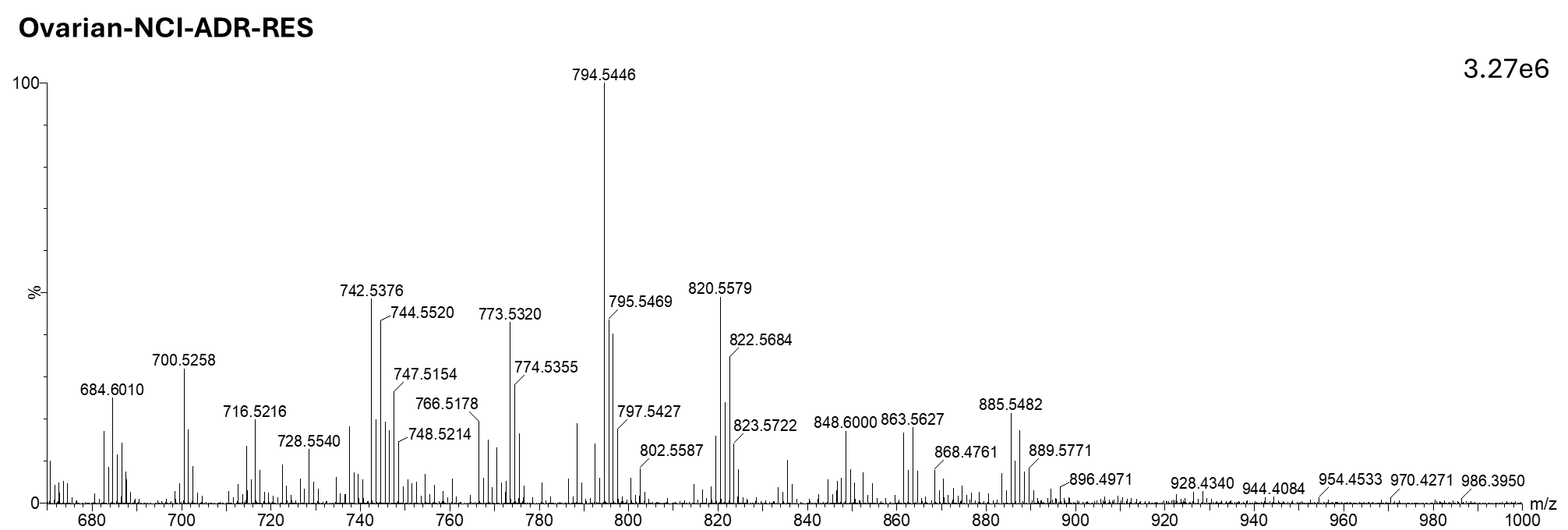


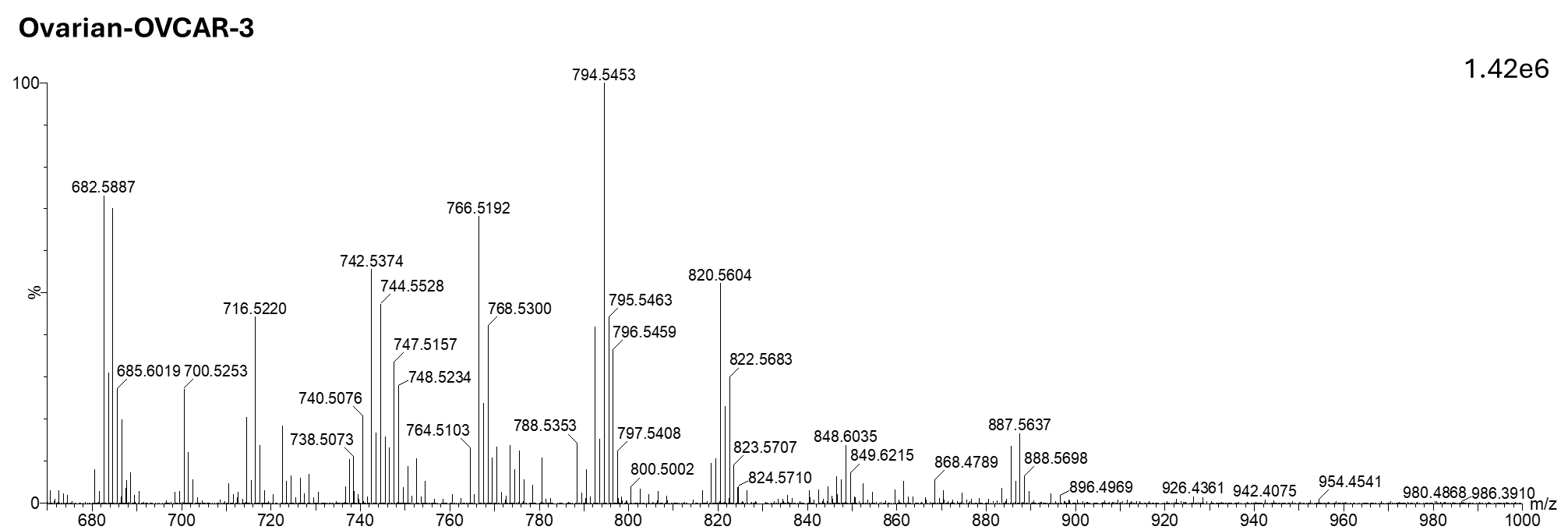


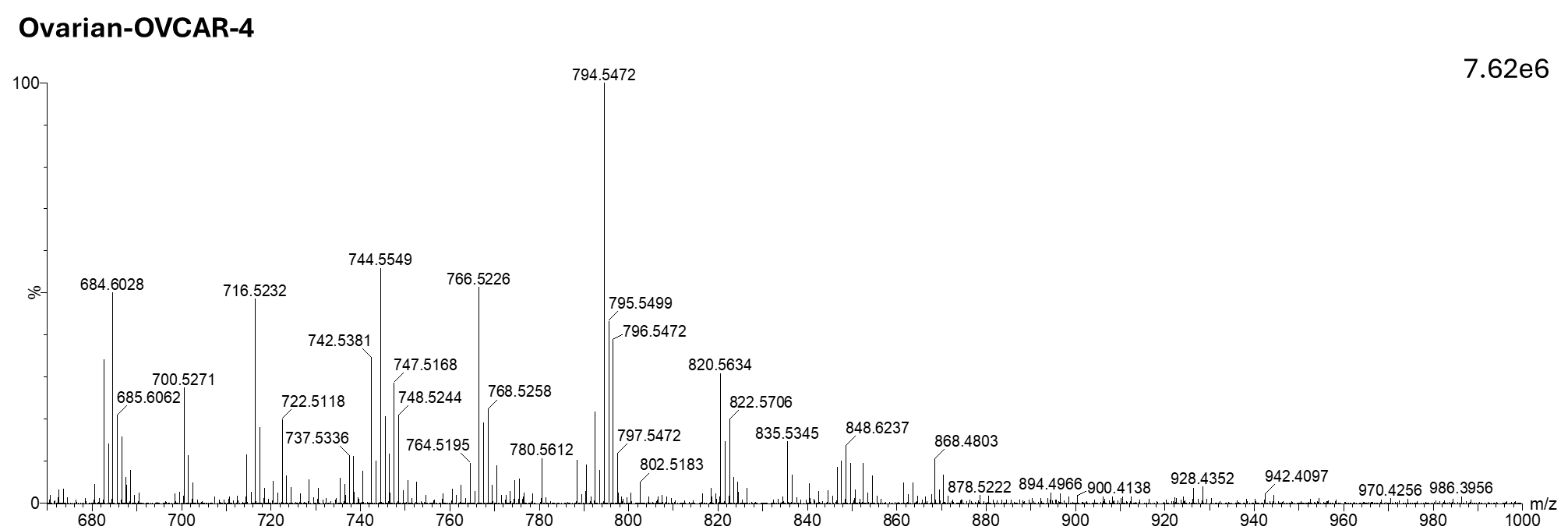


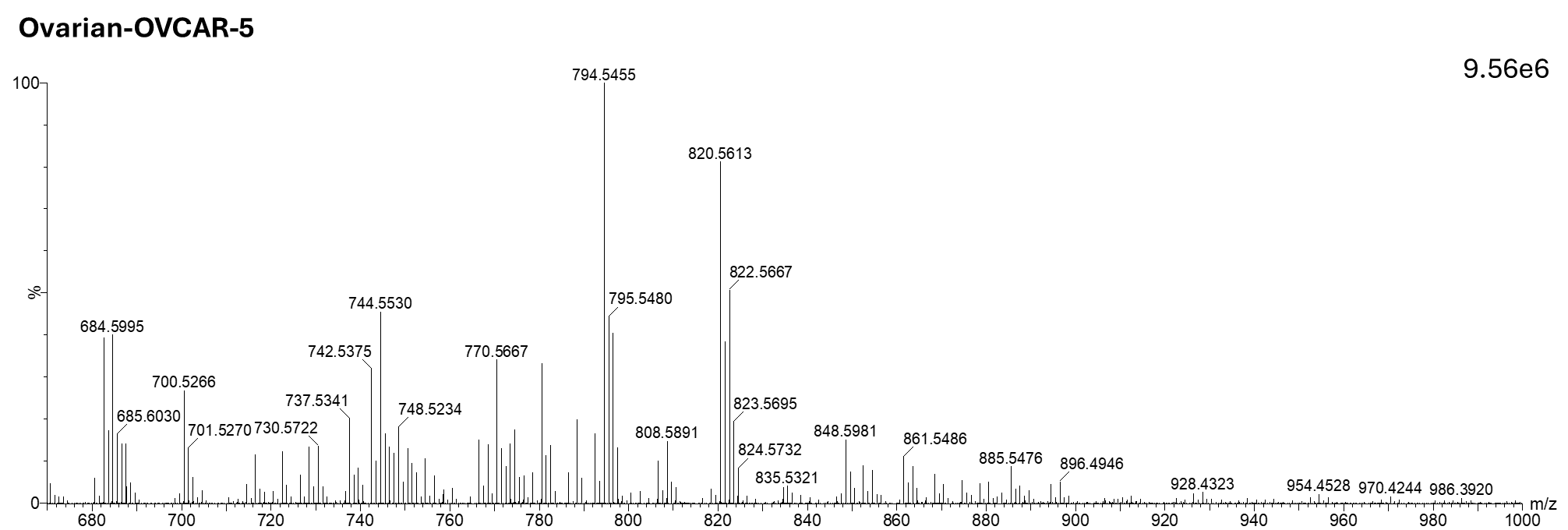


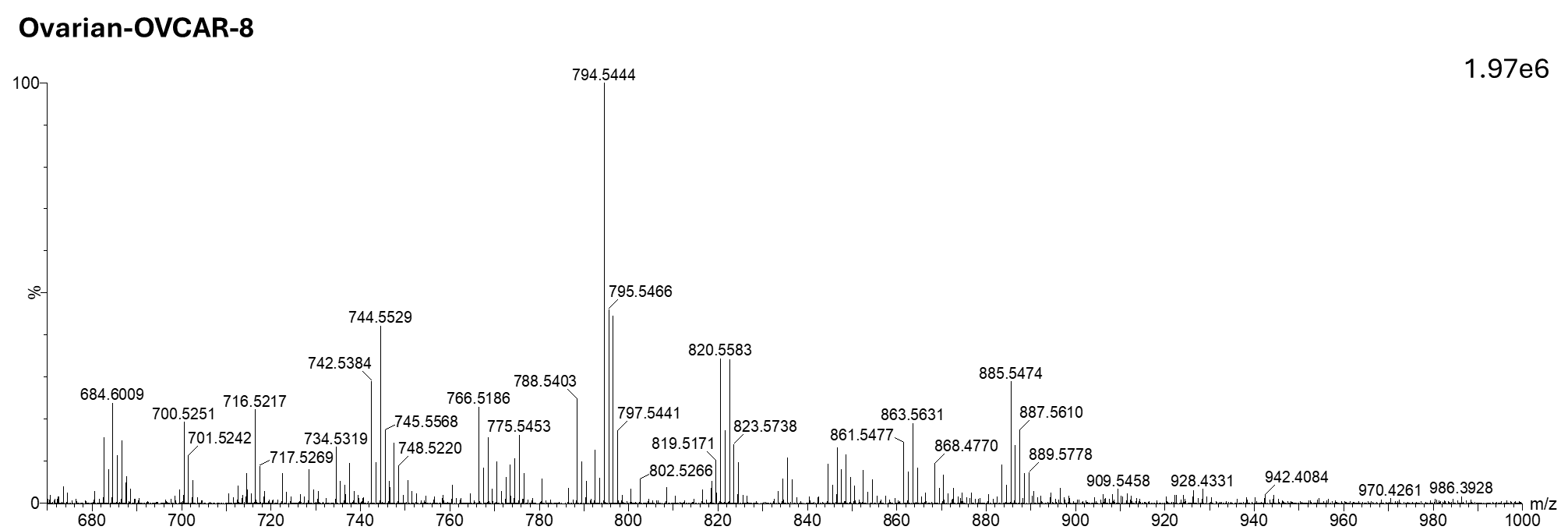


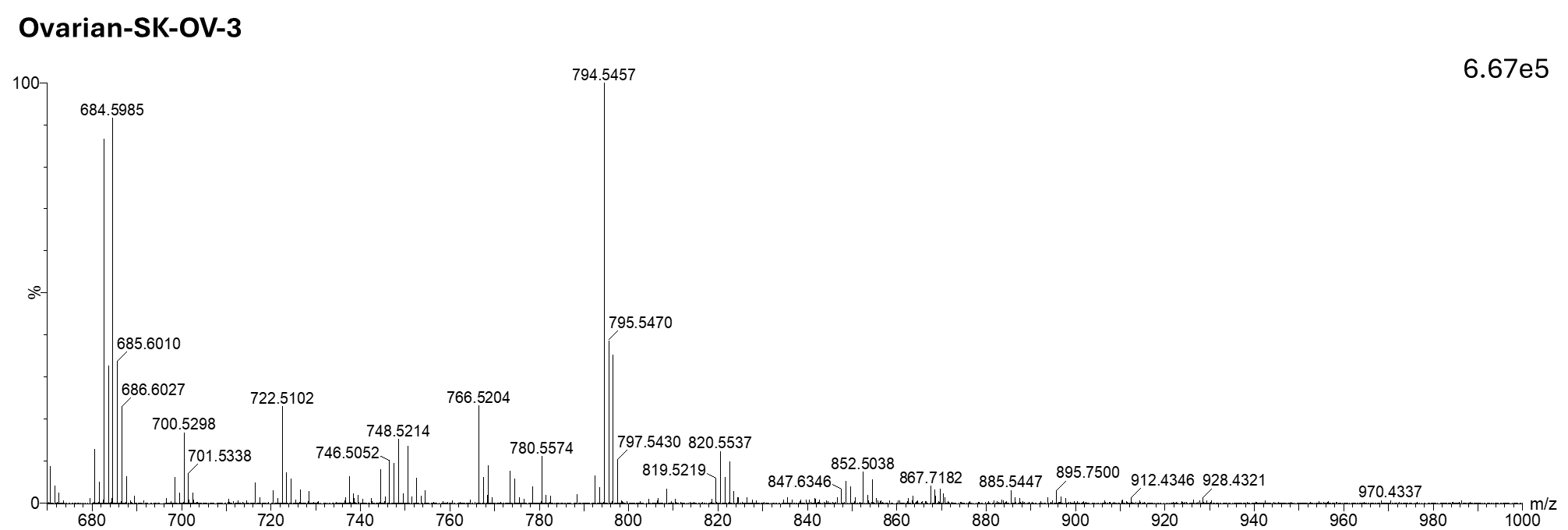


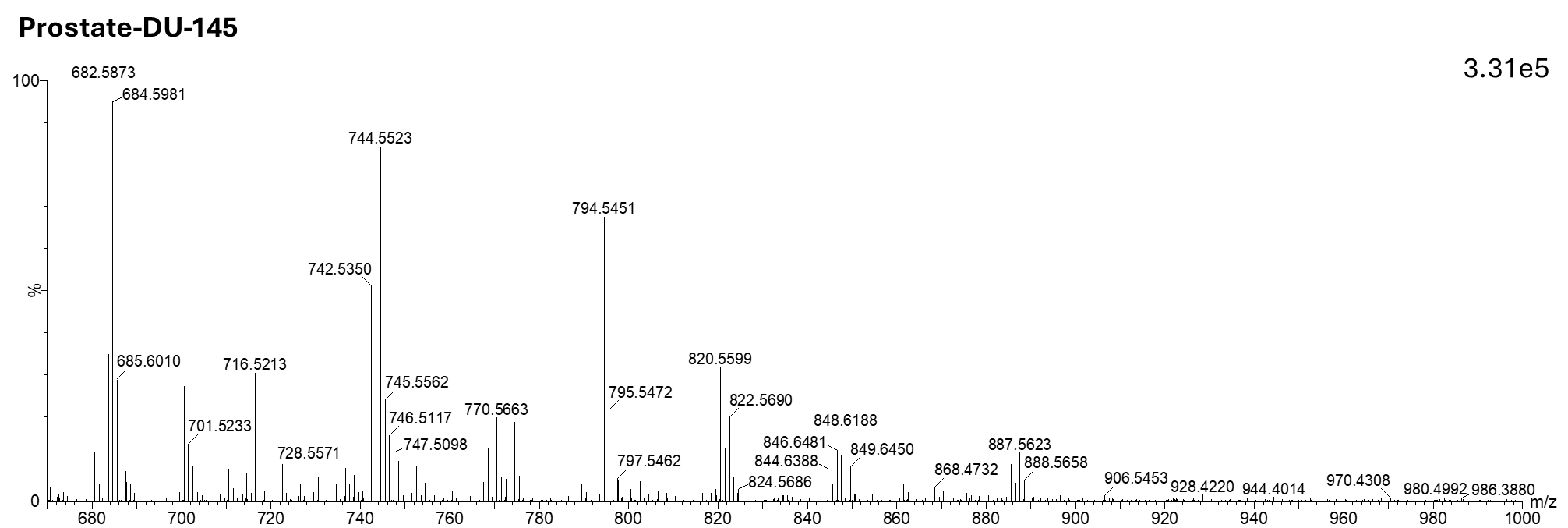


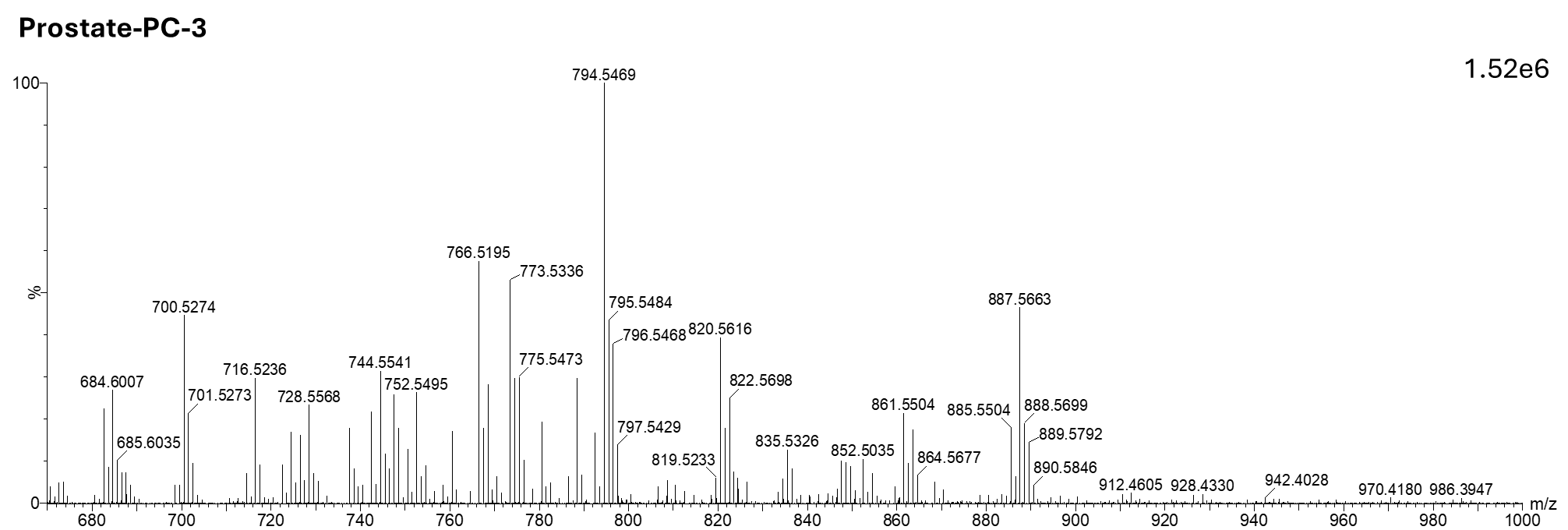


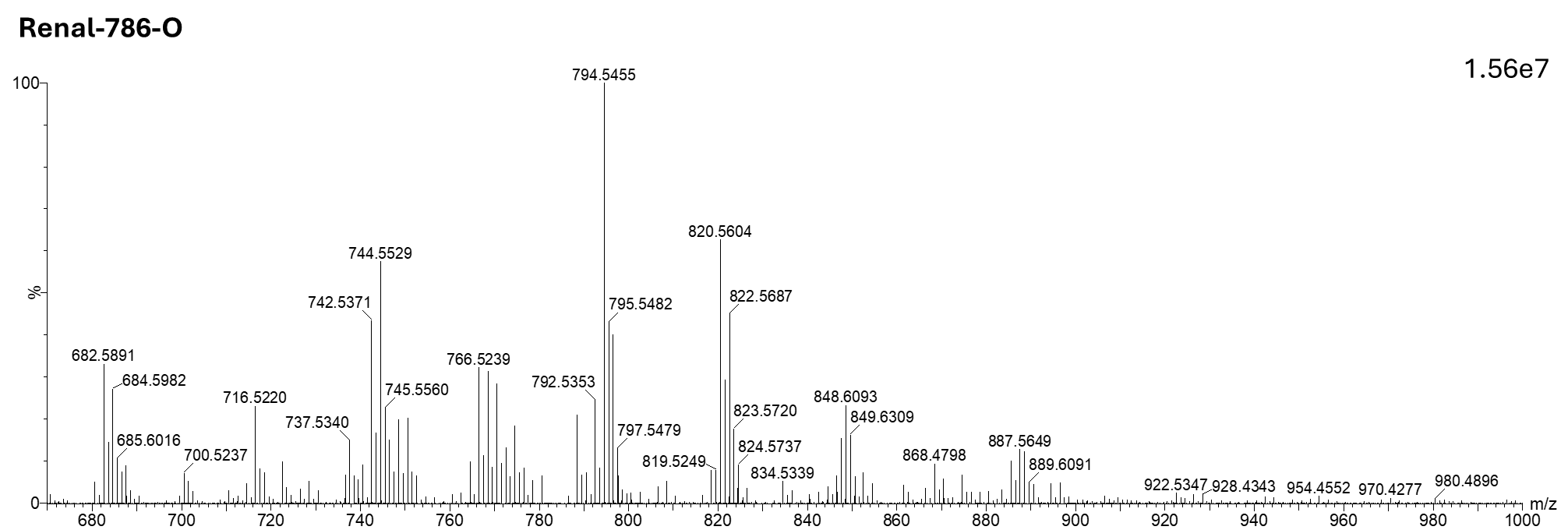


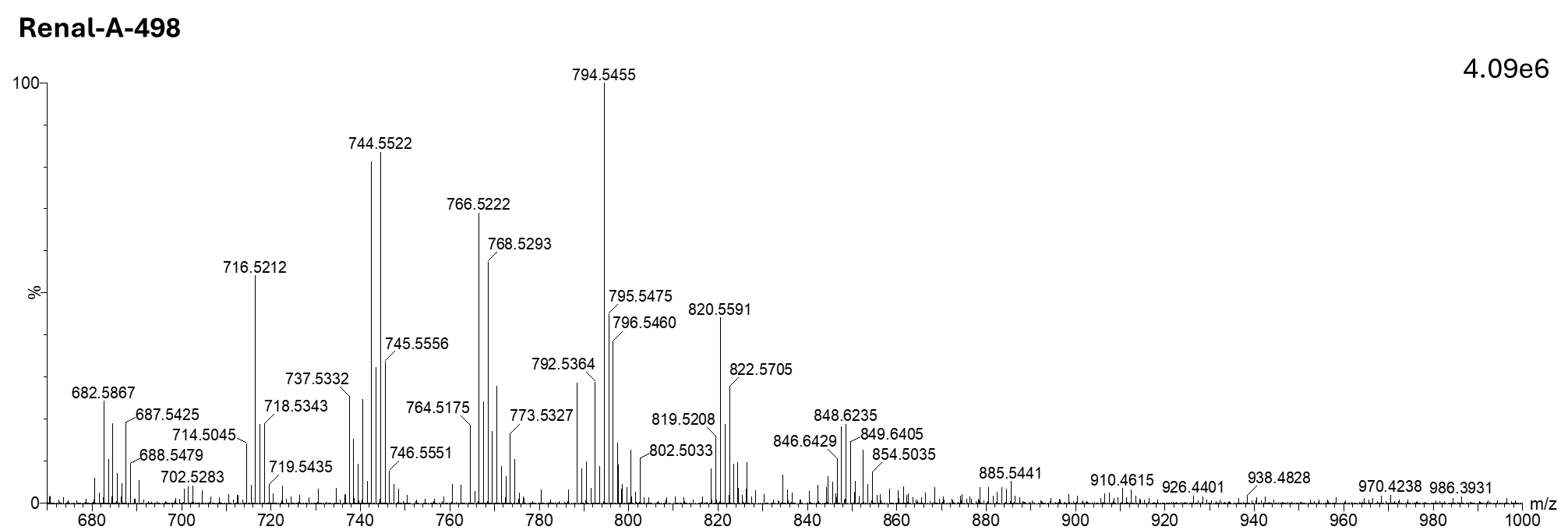


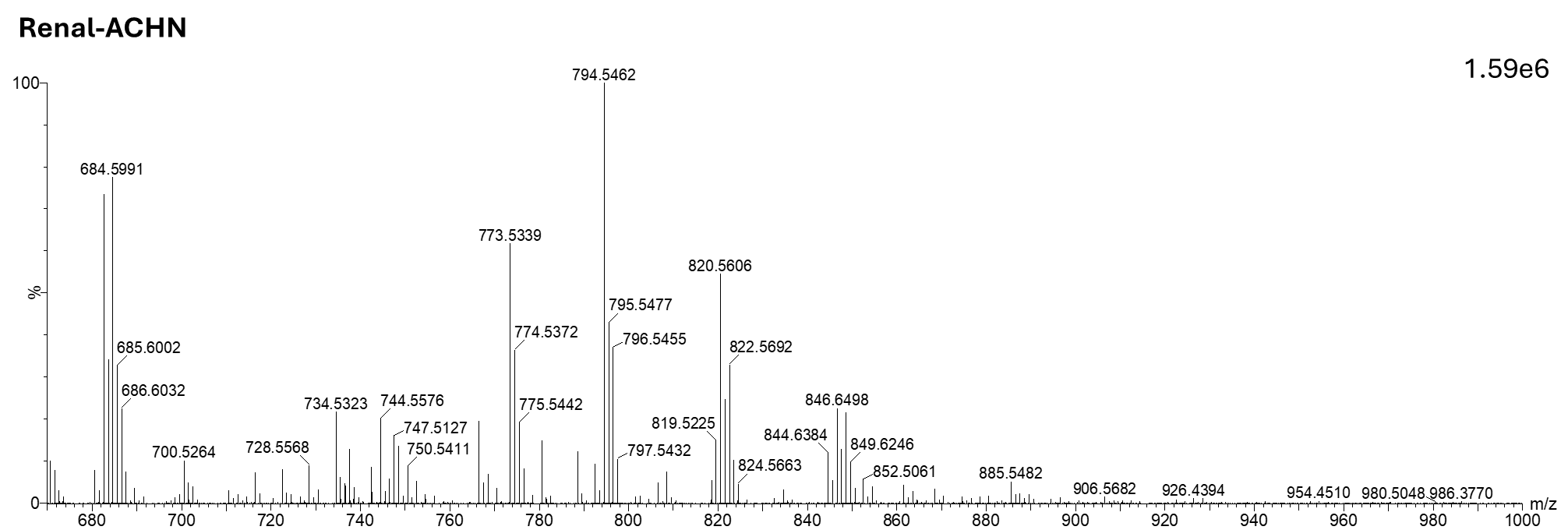


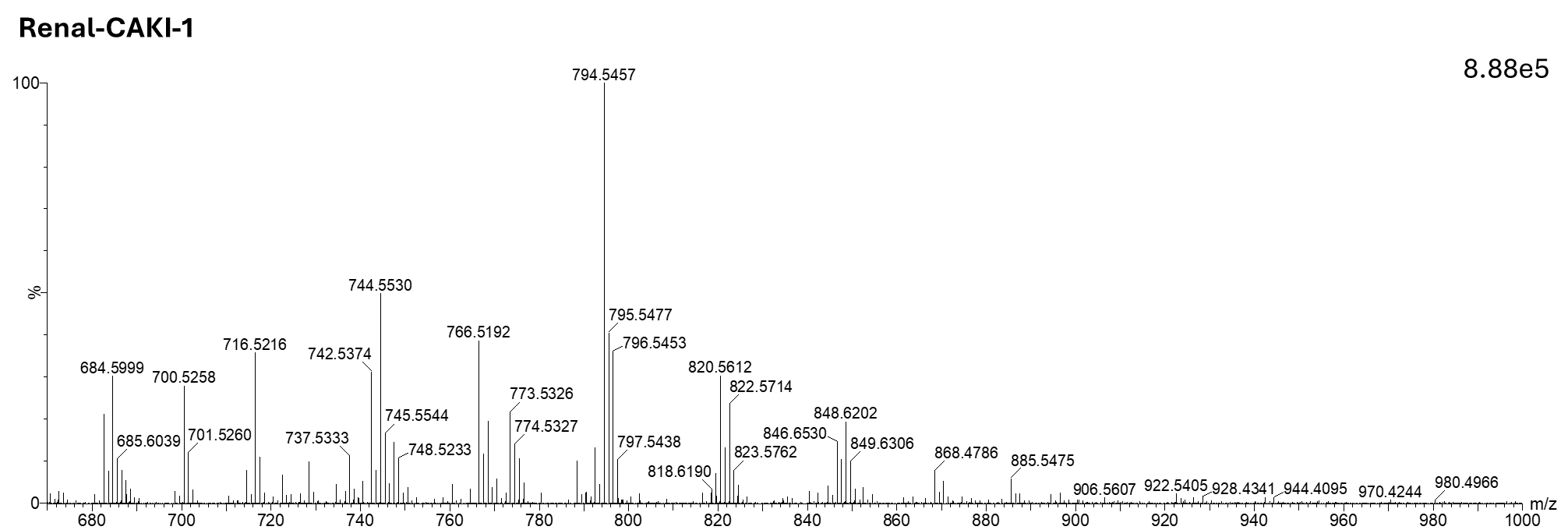


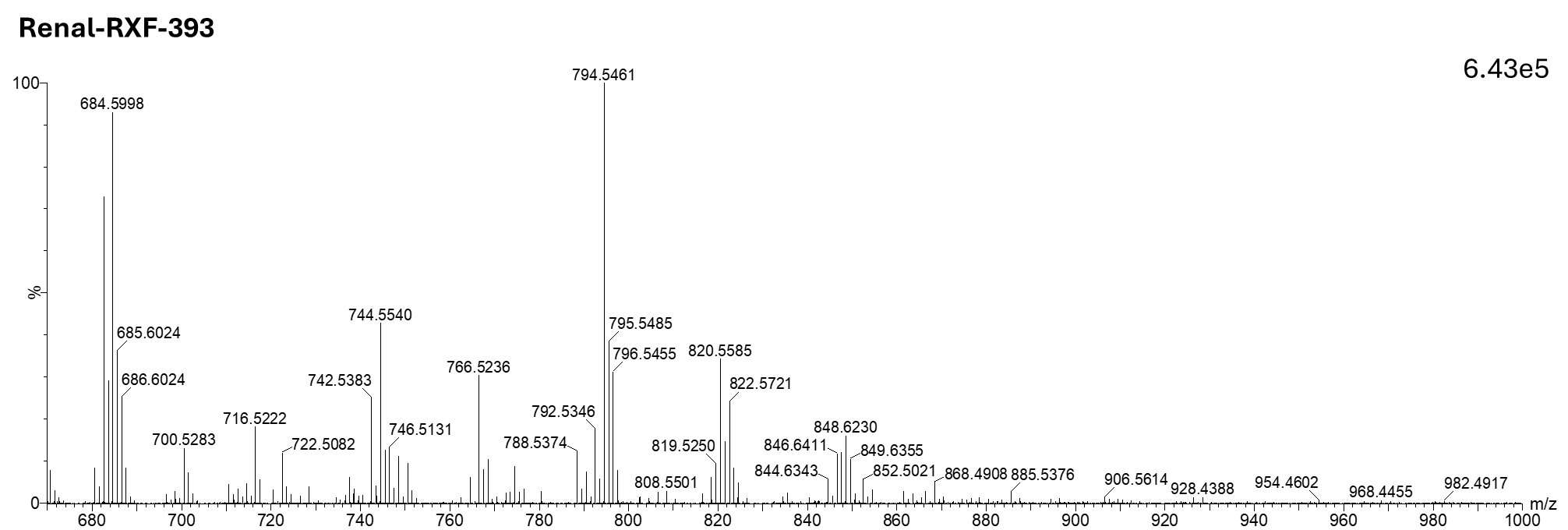


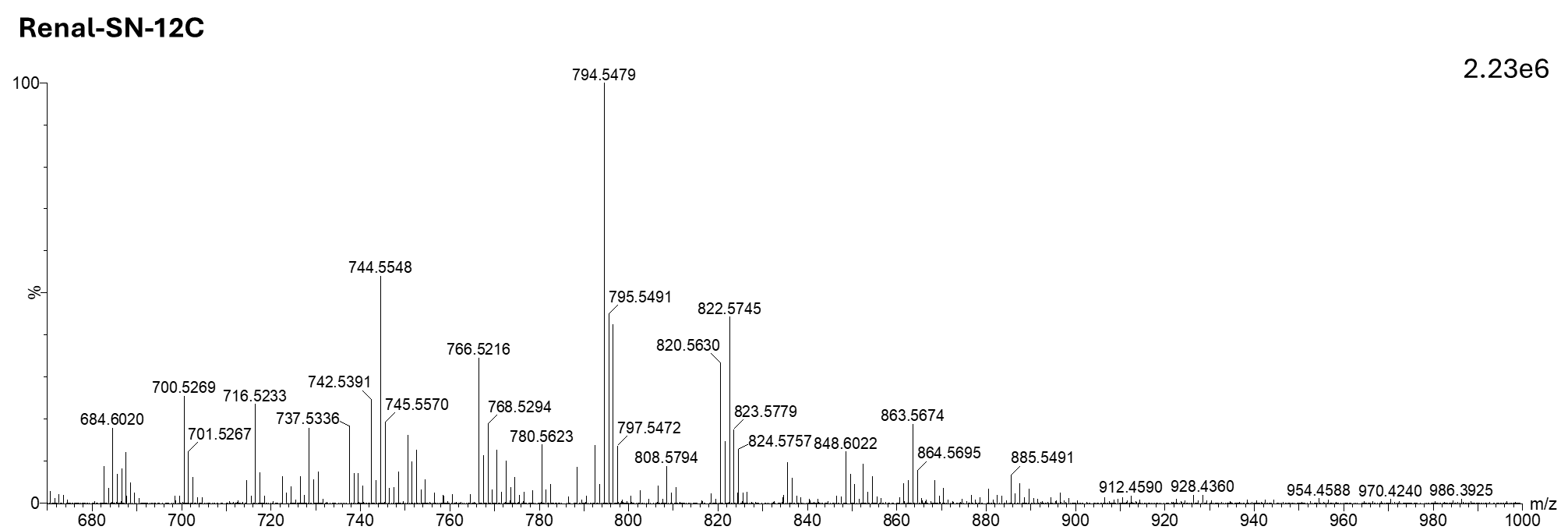


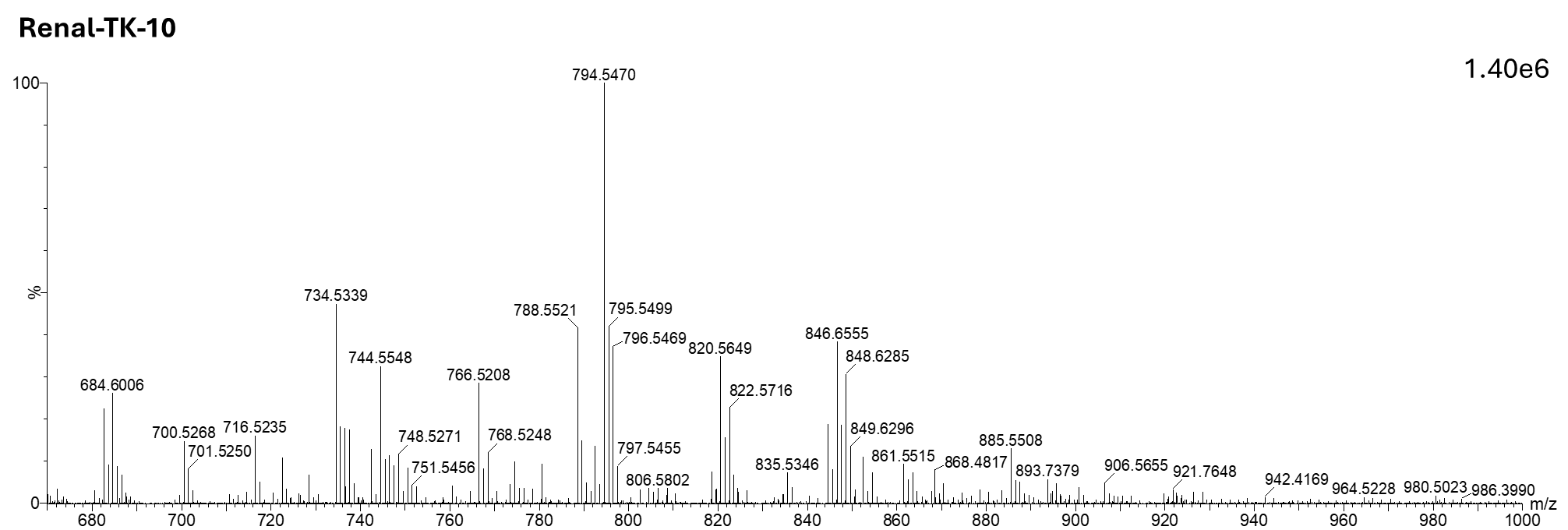


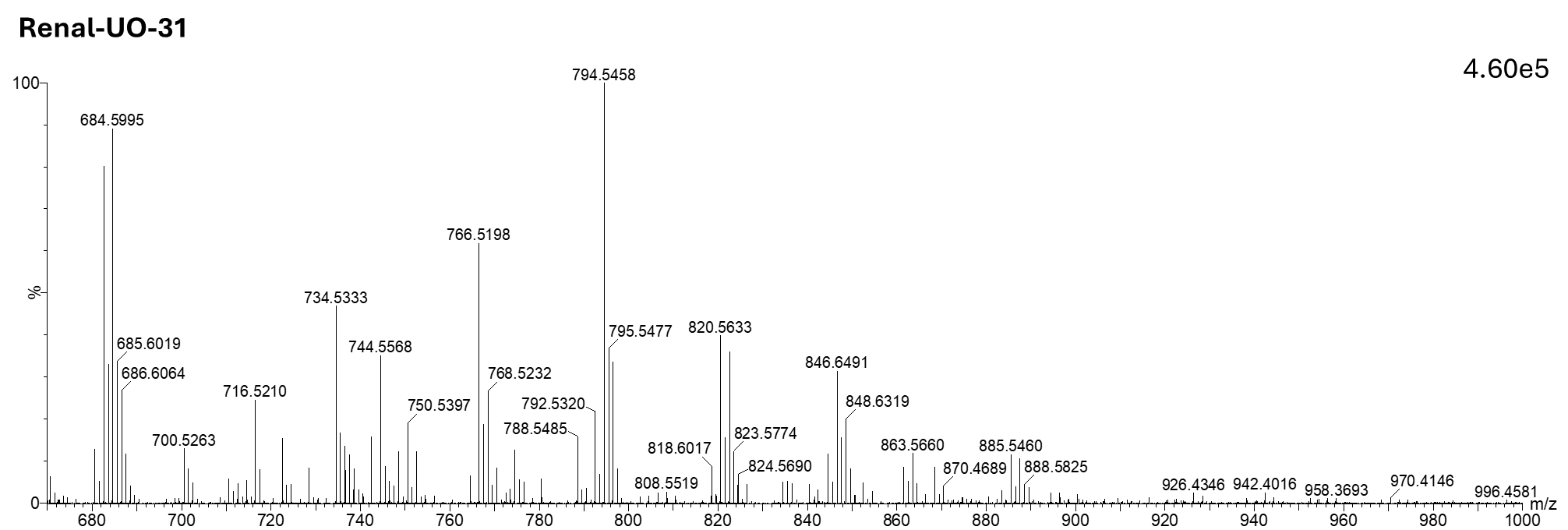


Fig. S3. NCI60 Laser Ablation - Rapid Evaporative Ionization Mass Spectrometry spectra for each cell line (Negative ion mode). The spectra are combined from 5 consecutive scans, lock massed on Leucine Enkephalin (554.2615 m/z) and are showcased from 670 – 1000 m/z to focus on the phospholipid region

Table S1. Peak annotations of pooled human blood serum based on inhouse database (negative ion mode)

| **Feature** | **Intensity** | **Compound name** | **Ppm error** | **Adduct** | **Molecular formula** |
| --- | --- | --- | --- | --- | --- |
| 89.0238 | 5938000 | Lactic acid | 6.948431 | [M-H]- | C3H6O3 |
| 201.0372 | 4265000 | Phenyllactic acid | 23.89774 | [M+Cl]- | C9H10O3 |
| 215.0321 | 9967000 | Paraxanthine_Theobromine_Theophylline | 9.426783 | [M+Cl]- | C7H8N4O2 |
| 215.0321 | 9967000 | Sorbose_Myo-inositol_Glucose | 3.216161 | [M+Cl]- | C6H12O6 |
| 217.0292 | 3412000 | 4-Hydroxyphenyllactic acid_3-(3-hydroxyphenyl)-3-hydroxypropionic acid (HPHPA)_Homovanillic acid | 8.70683 | [M+Cl]- | C9H10O4 |
| 227.2009 | 2903000 | Fusobacteria Marker 1 (FA (14:0))_Myristic acid FA(14:0) | 3.316789_3.317229 | [M-H]- | C14H28O2 |
| 253.2167 | 2988000 | Hexadecenoic acid FA(16:1) | 2.384434 | [M-H]- | C16H30O2 |
| 255.2324 | 32260000 | Palmitic acid FA(16:0) | 2.170096 | [M-H]- | C16H32O2 |
| 279.2324 | 16040000 | Linoleic Acid FA(18:2) | 1.983577 | [M-H]- | C18H32O2 |
| 281.2479 | 24070000 | Oleic Acid FA(18:1) | 2.502697 | [M-H]- | C18H34O2 |
| 283.2634 | 10160000 | Stearic acid FA(18:0) | 3.014782 | [M-H]- | C18H36O2 |
| 295.2271 | 3915000 | HydroxyLinoleic acid FA(18:2(OH)) | 2.603006 | [M-H]- | C18H32O3 |
| 303.2321 | 4558000 | Arachidonic acid FA(20:4) | 2.815921 | [M-H]- | C20H32O2 |
| 313.1907 | 20990000 | a-Linolenic Acid FA(18:3)_1_a-Linolenic Acid FA(18:3)_2 | 10.478426 | [M+Cl]- | C18H30O2 |
| 319.2268 | 4858000 | Stearic acid FA(18:0) | 44.267444 | [M+Cl]- | C18H36O2 |
| 337.1905 | 9127000 | Eicosapentaenoic acid FA(20:5) | 10.325748 | [M+Cl]- | C20H30O2 |
| 339.2045 | 16910000 | Arachidonic acid FA(20:4) | 15.128934 | [M+Cl]- | C20H32O2 |
| 341.2139 | 9894000 | Eicosatrienoic acid FA(20:3) | 33.355911 | [M+Cl]- | C20H34O2 |
| 395.1491 | 2866000 | Cortisone | 35.366614 | [M+Cl]- | C21H28O5 |
| 397.1607 | 6207000 | Cortisol | 45.384304 | [M+Cl]- | C21H30O5 |
| 530.301 | 4337000 | LPC(0:0/16:0)_LPC(16:0/0:0) | 1.680514 | [M+Cl]- | C24H50NO7P |
| 629.4886 | 4744000 | DG(16:0/18:1) | 4.967309 | [M+Cl]- | C37H70O5 |
| 684.603 | 3222000 | Cer(d18:1/24:0)_Cer(d19:1/23:0) | 5.400298 | [M+Cl]- | C42H83NO3 |
| 699.5469 | 9085000 | SM(d18:2/16:0)_1_SM(d18:2/16:0)_2 | 3.218694 | [M-H]- | C39H77N2O6P |
| 700.5502 | 4645000 | PE(O-34:2) | 30.740241 | [M-H]- | C39H76NO7P |
| 701.5475 | 4044000 | PA(18:0/18:1) | 49.630493 | [M-H]- | C39H75O8P |
| 701.5475 | 4044000 | SM(d18:1/16:0) | 18.24288 | [M-H]- | C39H79N2O6P |
| 715.5427 | 22330000 | SM(d19:1/16:0)_SM(d35:1); SM(d17:1/18:0) \| SM(d18:1/17:0) | 46.464083 | [M-H]- | C40H81N2O6P |
| 716.5455 | 10620000 | PE(16:0/18:1) | 30.587158 | [M-H]- | C39H76NO8P |
| 718.5437 | 3793000 | PE(18:0/16:0)_PC(16:0/15:0) | 6.222792 | [M-H]- | C39H78NO8P |
| 718.5437 | 3793000 | PC(O-16:0/16:0) | 44.413113 | [M-H]- | C40H82NO7P |
| 737.5322 | 2851000 | SM(d18:1/16:0) | 6.476258 | [M+Cl]- | C39H79N2O6P |
| 739.5402 | 5445000 | Rhizobiales Marker 1 (PA (39:3)) | 16.048635 | [M-H]- | C42H77O8P |
| 739.5402 | 5445000 | SM(d18:0/16:0) | 16.802834 | [M+Cl]- | C39H81N2O6P |
| 792.5295 | 4156000 | PE(18:0/22:5)_PE(20:1/20:4) | 32.027536 | [M-H]- | C45H80NO8P |
| 792.5295 | 4156000 | SHexCer(d18:2/16:0(OH)) | 45.144105 | [M-H]- | C40H75NO12S |
| 792.5295 | 4156000 | PC(16:0/18:2) | 2.595203 | [M+Cl]- | C42H80NO8P |
| 794.5414 | 5275000 | PE(18:0/22:4) | 36.66582 | [M-H]- | C45H82NO8P |
| 794.5414 | 5275000 | SHexCer(d18:1/16:0(OH)) | 40.309682 | [M-H]- | C40H77NO12S |
| 794.5414 | 5275000 | PC(16:0/18:1)_1_PC(16:0/18:1)_2_PC(16:0/18:1)_3 | 7.308288 | [M+Cl]- | C42H82NO8P |
| 820.559 | 3082000 | PC(36:2); PC(16:0/20:2) \| PC(18:1/18:1)_PC(18:0/18:2) | 4.700286 | [M+Cl]- | C44H84NO8P |
| 893.7314 | 3747000 | TG(52:2) | 6.313468 | [M+Cl]- | C55H102O6 |

Table S2. Peak annotations of K562 cell line based on inhouse database (negative ion mode)

| **Feature** | **Intensity** | **Compound name** | **Ppm error** | **Adduct** | **Molecular formula** |
| --- | --- | --- | --- | --- | --- |
| 89.022 | 750400 | Lactic acid | 27.167602 | [M-H]- | C3H6O3 |
| 93.0322 | 220700 | Phenol | 25.674107 | [M-H]- | C6H6O |
| 96.9643 | 60720 | Sulphate | 43.259235 | [M-H]- | H2SO4 |
| 123.0188 | 122600 | Butyric acid | 24.642618 | [M+Cl]- | C4H8O2 |
| 124.006 | 100100 | Taurine | 11.205623 | [M-H]- | C2H7NO3S |
| 128.0332 | 292600 | Oxoproline_Pyroglutamic acid | 16.532001 | [M-H]- | C5H7NO3 |
| 129.0535 | 249600 | Ketoleucine_3-Methyl-2-oxovaleric acid | 17.152127 | [M-H]- | C6H10O3 |
| 134.045 | 54750 | Adenine | 16.550735 | [M-H]- | C5H5N5 |
| 135.0302 | 156700 | Hypoxanthine | 7.661783 | [M-H]- | C5H4N4O |
| 137.0353 | 272600 | Nicotinamide N-oxide_Urocanic acid | 2.561231 | [M-H]- | C6H6N2O2 |
| 137.0353 | 272600 | Valeric acid (FA (5:0)) | 15.919586 | [M+Cl]- | C5H10O2 |
| 143.1058 | 282000 | Caprylic acid (FA (8:0)) | 13.651112 | [M-H]- | C8H16O2 |
| 145.0596 | 97700 | Glutamine | 15.62492 | [M-H]- | C5H10N2O3 |
| 146.044 | 587400 | Glutamic acid | 12.890333 | [M-H]- | C5H9NO4 |
| 146.044 | 587400 | Histamine | 34.567702 | [M+Cl]- | C5H9N3 |
| 157.1215 | 317500 | Pelargonic Acid (FA(9:0)) | 12.108826 | [M-H]- | C9H18O2 |
| 171.1374 | 292400 | Capric acid FA(10:0) | 9.662201 | [M-H]- | C10H20O2 |
| 199.169 | 436600 | Lauric acid FA(12:0) | 6.796092 | [M-H]- | C12H24O2 |
| 201.0368 | 698800 | Phenyllactic acid | 21.908011 | [M+Cl]- | C9H10O3 |
| 203.0123 | 228800 | Oxaloglutarate | 36.590434 | [M-H]- | C7H8O7 |
| 203.0123 | 228800 | p-Hydroxymandelic acid | 3.151149 | [M+Cl]- | C8H8O4 |
| 213.1849 | 95400 | Veillonella Marker 1 (FA (13:0)) | 5.176606 | [M-H]- | C13H26O2 |
| 217.0112 | 118900 | 1-Methyluric acid | 10.098824 | [M+Cl]- | C6H6N4O3 |
| 227.2007 | 537700 | Fusobacteria Marker 1 (FA (14:0))_Myristic acid FA(14:0) | 4.197065_4.197505 | [M-H]- | C14H28O2 |
| 253.2169 | 890400 | Hexadecenoic acid FA(16:1) | 1.594599 | [M-H]- | C16H30O2 |
| 255.2324 | 1343000 | Palmitic acid FA(16:0) | 2.170096 | [M-H]- | C16H32O2 |
| 265.1476 | 1789000 | Veillonella Marker 2 (FA (13:0;O)) | 37.700523 | [M+Cl]- | C13H26O3 |
| 265.1476 | 1789000 | Atenolol | 30.797671 | [M-H]- | C14H22N2O3 |
| 267.2323 | 129900 | Heptadecenoic acid FA(17:1) | 2.446854 | [M-H]- | C17H32O2 |
| 279.0385 | 103900 | Pseudouridine_Uridine | 1.568527 | [M+Cl]- | C9H12N2O6 |
| 279.2325 | 165400 | Linoleic Acid FA(18:2) | 1.625453 | [M-H]- | C18H32O2 |
| 281.2485 | 1299000 | Oleic Acid FA(18:1) | 0.369353 | [M-H]- | C18H34O2 |
| 283.2637 | 440800 | Stearic acid FA(18:0) | 1.9557 | [M-H]- | C18H36O2 |
| 293.1794 | 651800 | Flavobacteria Marker 1 (FA (15:0;O)) | 32.390654 | [M+Cl]- | C15H30O3 |
| 311.1698 | 483200 | Octadecatetraenoic acid FA(18:4) | 27.417333 | [M+Cl]- | C18H28O2 |
| 337.2055 | 174900 | Eicosapentaenoic acid FA(20:5) | 34.159032 | [M+Cl]- | C20H30O2 |
| 339.2017 | 436000 | Arachidonic acid FA(20:4) | 23.383416 | [M+Cl]- | C20H32O2 |
| 365.2411 | 117900 | Docosapentaenoic acid FA(22:5)_1_Docosapentaenoic acid FA(22:5)_2 | 43.310583 | [M+Cl]- | C22H34O2 |
| 365.2411 | 117900 | MG(16:0) | 14.541635 | [M+Cl]- | C19H38O4 |
| 367.2451 | 73820 | Docosatetraenoic acid FA(22:4) | 11.349552 | [M+Cl]- | C22H36O2 |
| 391.263 | 143300 | MG(18:1)_1_MG(18:1)_2 | 2.398955 | [M+Cl]- | C21H40O4 |
| 393.2717 | 152200 | Tetracosapentaenoic acid FA(24:5) | 38.442891 | [M+Cl]- | C24H38O2 |
| 393.2717 | 152200 | 23-Norcholic acid | 17.943184 | [M-H]- | C23H38O5 |
| 441.2551 | 51460 | 7-Ketodeoxycholic acid | 31.226495 | [M+Cl]- | C24H38O5 |
| 465.3073 | 149300 | Bacteroidetes Marker 2 (Cholesterol Sulfate)_Cholesterol Sulfate_1_Cholesterol Sulfate_2 | 6.220487_6.222636_6.222636 | [M-H]- | C27H46O4S |
| 572.4857 | 169800 | Bacteroidaceae Marker 4 (Cer (34:1;O2))_Cer(d18:1/16:0) | 7.345949_7.342456 | [M+Cl]- | C34H67NO3 |
| 595.3021 | 383900 | LPI(0:0/18:2)_LPI(18:2/0:0) | 22.195644 | [M-H]- | C27H49O12P |
| 682.5957 | 63140 | Cer(d18:1/24:1)_Cer(d18:2/24:0) | 6.816556 | [M+Cl]- | C42H81NO3 |
| 684.6071 | 61510 | Cer(d18:1/24:0)_Cer(d19:1/23:0) | 0.588542 | [M+Cl]- | C42H83NO3 |
| 700.5342 | 252200 | PE(O-34:2) | 7.900348 | [M-H]- | C39H76NO7P |
| 701.5302 | 139000 | PA(18:0/18:1) | 24.969499 | [M-H]- | C39H75O8P |
| 701.5302 | 139000 | SM(d18:1/16:0) | 42.9022 | [M-H]- | C39H79N2O6P |
| 716.5317 | 53900 | PE(16:0/18:1) | 11.327499 | [M-H]- | C39H76NO8P |
| 726.5501 | 55050 | PE(O-18:1/18:2) and/or PE(P-18:0/18:2) | 7.964304 | [M-H]- | C41H78NO7P |
| 726.5501 | 55050 | PC(O-16:0/14:0) | 40.064802 | [M+Cl]- | C38H78NO7P |
| 728.5662 | 134300 | PE(O-18:1/18:1) and/or PE(P-18:0/18:1) | 8.559927 | [M-H]- | C41H80NO7P |
| 742.5475 | 205000 | PE(18:0/18:2)_PC(15:0/18:2) | 11.136678 | [M-H]- | C41H78NO8P |
| 742.5475 | 205000 | PC(O-16:0/18:2) | 37.86036 | [M-H]- | C42H82NO7P |
| 744.5627 | 403800 | PC(O-16:0/18:1) | 38.362363 | [M-H]- | C42H84NO7P |
| 744.5627 | 403800 | PE(36:1); PE(18:0/18:1) \| PE(18:1/18:0)_PC(33:1); PC(15:0/18:1) \| PC(16:0/17:1) | 10.504558 | [M-H]- | C41H80NO8P |
| 747.5236 | 52420 | PG(34:1) | 7.275301 | [M-H]- | C40H77O10P |
| 788.5514 | 56490 | PS(18:0/18:1) | 8.492125 | [M-H]- | C42H80NO10P |
| 788.5514 | 56490 | PC(O-16:1/22:6) and/or PC(P-16:0/22:6) | 10.85977 | [M-H]- | C46H80NO7P |
| 794.557 | 278400 | PE(18:0/22:4) | 17.032572 | [M-H]- | C45H82NO8P |
| 794.557 | 278400 | PC(16:0/18:1)_1_PC(16:0/18:1)_  2_PC(16:0/18:1)_3 | 12.325536 | [M+Cl]- | C42H82NO8P |
| 796.5569 | 57590 | PC(34:0); PC(18:0/16:0) \| PC(16:0/18:0) | 7.47823 | [M+Cl]- | C42H84NO8P |
| 820.574 | 63320 | PC(36:2); PC(16:0/20:2) \| PC(18:1/18:1)_PC(18:0/18:2) | 13.579849 | [M+Cl]- | C44H84NO8P |
| 822.5837 | 77800 | PC(18:0/18:1)_PC(36:1); PC(16:0/20:1) \| PC(18:0/18:1) | 6.3131 | [M+Cl]- | C44H86NO8P |
| 822.5837 | 77800 | PE(42:4) | 22.039314 | [M-H]- | C47H86NO8P |
| 848.6113 | 153100 | PC(38:2) | 20.200964 | [M+Cl]- | C46H88NO8P |
| 861.5605 | 170400 | PI(36:2); PI(18:0/18:2)\|PI(18:1/18:1) | 12.357288 | [M-H]- | C45H83O13P |
| 863.5762 | 655400 | PI(18:0/18:1) | 12.386345 | [M-H]- | C45H85O13P |
| 887.5764 | 53910 | PI(18:0/20:3)_1_PI(18:0/20:3)_  2_PI(18:0/20:3)_3 | 12.27675 | [M-H]- | C47H85O13P |

Table S3. Peak annotations of mouse brain tissue based on inhouse database (negative ion mode)

| **Feature** | **Intensity** | **Compound name** | **Ppm error** | **Adduct** | **Molecular formula** |
| --- | --- | --- | --- | --- | --- |
| 89.0236 | 104200 | Lactic acid | 9.195005 | [M-H]- | C3H6O3 |
| 128.034 | 49380 | Oxoproline_Pyroglutamic acid | 10.283725 | [M-H]- | C5H7NO3 |
| 135.0304 | 34150 | Hypoxanthine | 6.180644 | [M-H]- | C5H4N4O |
| 145.0608 | 56110 | Glutamine | 7.352587 | [M-H]- | C5H10N2O3 |
| 146.0448 | 30590 | Glutamic acid | 7.412602 | [M-H]- | C5H9NO4 |
| 146.0448 | 30590 | Histamine | 29.09009 | [M+Cl]- | C5H9N3 |
| 151.0269 | 70280 | Xanthine | 4.968808 | [M-H]- | C5H4N4O2 |
| 174.0399 | 57240 | N-Acetylaspartic acid | 5.148103 | [M-H]- | C6H9NO5 |
| 201.0375 | 175000 | Phenyllactic acid | 25.390037 | [M+Cl]- | C9H10O3 |
| 227.2008 | 33750 | Fusobacteria Marker 1 (FA (14:0))_Myristic acid FA(14:0) | 3.756927_3.757367 | [M-H]- | C14H28O2 |
| 253.2164 | 68400 | Hexadecenoic acid FA(16:1) | 3.569187 | [M-H]- | C16H30O2 |
| 255.2324 | 348800 | Palmitic acid FA(16:0) | 2.170096 | [M-H]- | C16H32O2 |
| 265.1474 | 52270 | Veillonella Marker 2 (FA (13:0;O)) | 38.454791 | [M+Cl]- | C13H26O3 |
| 265.1474 | 52270 | Atenolol | 31.551944 | [M-H]- | C14H22N2O3 |
| 267.0727 | 59080 | Inosine | 2.96952 | [M-H]- | C10H12N4O5 |
| 279.2327 | 29900 | Linoleic Acid FA(18:2) | 0.909205 | [M-H]- | C18H32O2 |
| 281.2482 | 314100 | Oleic Acid FA(18:1) | 1.436025 | [M-H]- | C18H34O2 |
| 283.2637 | 340700 | Stearic acid FA(18:0) | 1.9557 | [M-H]- | C18H36O2 |
| 303.2323 | 213400 | Arachidonic acid FA(20:4) | 2.156362 | [M-H]- | C20H32O2 |
| 309.2789 | 30090 | Eicosenoic acid FA(20:1) | 3.246509 | [M-H]- | C20H38O2 |
| 311.1691 | 69800 | Octadecatetraenoic acid FA(18:4) | 29.666847 | [M+Cl]- | C18H28O2 |
| 327.2328 | 128600 | Docosahexaenoic FA(22:6) | 0.470246 | [M-H]- | C22H32O2 |
| 331.264 | 45890 | Docosatetraenoic acid FA(22:4) | 0.766699 | [M-H]- | C22H36O2 |
| 339.2008 | 59620 | Arachidonic acid FA(20:4) | 26.036643 | [M+Cl]- | C20H32O2 |
| 598.4999 | 56800 | Cer(d18:2/18:0) | 4.592202 | [M+Cl]- | C36H69NO3 |
| 600.5145 | 156500 | Cer(d16:1/20:0)_Cer(d18:1/18:0) | 2.836442 | [M+Cl]- | C36H71NO3 |
| 673.4861 | 52710 | Elizabethkingia Marker 1 (DG (38:7)) | 38.121646 | [M+Cl]- | C41H66O5 |
| 673.4861 | 52710 | PA(16:0/18:1) | 7.003044 | [M-H]- | C37H71O8P |
| 699.5021 | 66240 | PA(36:2);PA(18:0/18:2) \| PA(18:1/18:1) | 7.242947 | [M-H]- | C39H73O8P |
| 700.5281 | 117600 | PE(O-34:2) | 0.807362 | [M-H]- | C39H76NO7P |
| 701.528 | 72640 | PA(18:0/18:1) | 21.833418 | [M-H]- | C39H75O8P |
| 701.528 | 72640 | SM(d18:1/16:0) | 46.038067 | [M-H]- | C39H79N2O6P |
| 702.5385 | 35400 | PE(O-34:1) | 8.277884 | [M-H]- | C39H78NO7P |
| 716.5298 | 64050 | PE(16:0/18:1) | 8.675807 | [M-H]- | C39H76NO8P |
| 718.543 | 33110 | PE(18:0/16:0)_PC(16:0/15:0) | 5.248593 | [M-H]- | C39H78NO8P |
| 718.543 | 33110 | PC(O-16:0/16:0) | 45.387262 | [M-H]- | C40H82NO7P |
| 722.516 | 91330 | PE(O-16:1/20:4) and/or PE(P-16:0/20:4) | 4.133379 | [M-H]- | C41H74NO7P |
| 723.515 | 42170 | SM(d17:1/16:0) | 8.743875 | [M+Cl]- | C38H77N2O6P |
| 726.5494 | 138600 | PE(O-18:1/18:2) and/or PE(P-18:0/18:2) | 7.000839 | [M-H]- | C41H78NO7P |
| 726.5494 | 138600 | PC(O-16:0/14:0) | 39.101307 | [M+Cl]- | C38H78NO7P |
| 727.5497 | 82670 | SM(d18:1/18:1)_SM(d18:2/18:0) | 36.076756 | [M-H]- | C41H81N2O6P |
| 727.5497 | 82670 | PA(38:2) | 29.371255 | [M-H]- | C41H77O8P |
| 728.5634 | 128300 | PE(O-18:1/18:1) and/or PE(P-18:0/18:1) | 4.716729 | [M-H]- | C41H80NO7P |
| 729.5682 | 55910 | Gardnerella Marker 1 (MGDG (32:0)) | 21.898939 | [M-H]- | C41H78O10 |
| 729.5682 | 55910 | Rhodospirillales Marker 5 (PA (39:1;O)) | 16.676134 | [M-H]- | C42H83O7P |
| 729.5682 | 55910 | SM(d16:1/20:0)_SM(d18:1/18:0) | 32.070791 | [M-H]- | C41H83N2O6P |
| 738.512 | 56130 | PE(16:0/20:4) | 5.513035 | [M-H]- | C41H74NO8P |
| 738.512 | 56130 | PE(O-34:1) | 12.177826 | [M+Cl]- | C39H78NO7P |
| 742.5451 | 71390 | PE(18:0/18:2)_PC(15:0/18:2) | 7.904525 | [M-H]- | C41H78NO8P |
| 742.5451 | 71390 | PC(O-16:0/18:2) | 41.092354 | [M-H]- | C42H82NO7P |
| 744.5605 | 108400 | PC(O-16:0/18:1) | 41.317004 | [M-H]- | C42H84NO7P |
| 744.5605 | 108400 | PE(36:1); PE(18:0/18:1) \| PE(18:1/18:0)_PC(33:1); PC(15:0/18:1) \| PC(16:0/17:1) | 7.549773 | [M-H]- | C41H80NO8P |
| 746.5171 | 228800 | PE(O-16:1/22:6) and/or PE(P-16:0/22:6) | 5.47401 | [M-H]- | C43H74NO7P |
| 747.5148 | 185900 | PG(34:1) | 4.496988 | [M-H]- | C40H77O10P |
| 748.5279 | 194900 | PE(O-16:0/22:6)_PE(O-16:1/22:5) and/or PE(P-16:0/22:5)_PE(O-18:2/20:4) and/or PE(P-18:1/20:4) | 1.020108 | [M-H]- | C43H76NO7P |
| 749.528 | 74840 | PG(34:0) | 7.753593 | [M-H]- | C40H79O10P |
| 750.5485 | 314400 | PE(O-18:1/20:4) and/or PE(P-18:0/20:4) | 5.577179 | [M-H]- | C43H78NO7P |
| 751.5512 | 136600 | SM(d19:1/16:0)_SM(d35:1); SM(d17:1/18:0) \| SM(d18:1/17:0) | 1.898177 | [M+Cl]- | C40H81N2O6P |
| 754.5825 | 35510 | PC(O-16:0/16:0) | 40.034097 | [M+Cl]- | C40H82NO7P |
| 754.5825 | 35510 | PE(O-20:1/18:2) and/or PE(P-20:0/18:2) | 9.125421 | [M-H]- | C43H82NO7P |
| 762.5134 | 151900 | PE(O-18:1/18:2) and/or PE(P-18:0/18:2) | 9.955896 | [M+Cl]- | C41H78NO7P |
| 763.5164 | 67630 | Staphylococcaceae Marker 1 (PG (35:0)) | 43.299854 | [M-H]- | C41H81O10P |
| 763.5164 | 67630 | SM(d18:1/18:1)_SM(d18:2/18:0) | 47.444772 | [M+Cl]- | C41H81N2O6P |
| 763.5164 | 67630 | Narasin | 21.235455 | [M-H]- | C43H72O11 |
| 763.5164 | 67630 | PA(38:2) | 14.918592 | [M+Cl]- | C41H77O8P |
| 764.5265 | 87160 | PE(O-18:1/18:1) and/or PE(P-18:0/18:1) | 13.265002 | [M+Cl]- | C41H80NO7P |
| 764.5265 | 87160 | PC(14:0/18:2) | 34.327549 | [M+Cl]- | C40H76NO8P |
| 764.5265 | 87160 | PE(38:5) | 3.818613 | [M-H]- | C43H76NO8P |
| 765.5294 | 32620 | Gardnerella Marker 1 (MGDG (32:0)) | 0.651079 | [M+Cl]- | C41H78O10 |
| 765.5294 | 32620 | Rhodospirillales Marker 5 (PA (39:1;O)) | 36.110412 | [M+Cl]- | C42H83O7P |
| 765.5294 | 32620 | Propionibacteriaceae Marker 1 (PI (30:1;O)) | 48.411222 | [M-H]- | C39H75O12P |
| 766.5446 | 552300 | PC(16:0/16:1)_PC(32:1); PC(14:0/18:1) \| PC(16:0/16:1) | 37.433431 | [M+Cl]- | C40H78NO8P |
| 766.5446 | 552300 | PE(18:0/20:4) | 7.007234 | [M-H]- | C43H78NO8P |
| 766.5446 | 552300 | PE(O-36:1) | 10.036602 | [M+Cl]- | C41H82NO7P |
| 768.549 | 64220 | PE(18:0/20:3) | 7.65538 | [M-H]- | C43H80NO8P |
| 768.549 | 64220 | PC(16:0/16:0) | 22.696817 | [M+Cl]- | C40H80NO8P |
| 772.5318 | 83470 | PE(O-40:8) | 4.057351 | [M-H]- | C45H76NO7P |
| 773.533 | 39490 | PG(36:2) | 1.049185 | [M-H]- | C42H79O10P |
| 774.5486 | 410100 | PE(O-18:1/22:6) and/or PE(P-18:0/22:6) | 5.533473 | [M-H]- | C45H79NO7P |
| 775.5524 | 191200 | PG(36:1) | 3.788823 | [M-H]- | C42H81O10P |
| 776.5603 | 137500 | PC(14:0/22:6) | 47.289511 | [M-H]- | C44H76NO8P |
| 776.5603 | 137500 | PC(O-16:1/18:2) and/or PC(P-16:0/18:2) | 30.466586 | [M+Cl]- | C42H80NO7P |
| 776.5603 | 137500 | PE(O-40:6) | 0.430643 | [M-H]- | C45H80NO7P |
| 777.5657 | 45030 | PG(36:0) | 0.756747 | [M-H]- | C42H83O10P |
| 778.579 | 158300 | PE(O-18:1/22:4) and/or PE(P-18:0/22:4)_  PE(O-20:1/20:4) and/or PE(P-20:0/20:4) | 4.348736 | [M-H]- | C45H82NO7P |
| 778.579 | 158300 | PC(O-16:0/18:2) | 34.304465 | [M+Cl]- | C42H82NO7P |
| 779.5832 | 65050 | Rhodospirillales Marker 6 (PA (40:1;O)) | 13.47587 | [M+Cl]- | C43H85O7P |
| 779.5832 | 65050 | Gardnerella Marker 2 (SM (39:5;O)) | 15.823347 | [M-H]- | C44H81N2O7P |
| 779.5832 | 65050 | SM(d19:1/18:0)_SM(d37:1); SM(d17:1/20:0) \| SM(d18:1/19:0) | 0.932138 | [M+Cl]- | C42H85N2O6P |
| 779.5832 | 65050 | TG(44:3) | 16.665012 | [M+Cl]- | C47H84O6 |
| 788.5372 | 78580 | PS(18:0/18:1) | 9.515732 | [M-H]- | C42H80NO10P |
| 788.5372 | 78580 | PC(14:0/20:4) | 46.852769 | [M+Cl]- | C42H76NO8P |
| 788.5372 | 78580 | PC(O-16:1/22:6) and/or PC(P-16:0/22:6) | 28.867278 | [M-H]- | C46H80NO7P |
| 790.5446 | 580500 | PE(18:0/22:6) | 6.788303 | [M-H]- | C45H78NO8P |
| 790.5446 | 580500 | PC(16:0/18:3)_PC(16:1/18:2) | 36.296955 | [M+Cl]- | C42H78NO8P |
| 790.5446 | 580500 | PE(O-20:1/18:2) and/or PE(P-20:0/18:2) | 9.730134 | [M+Cl]- | C43H82NO7P |
| 790.5446 | 580500 | PC(O-38:6) | 39.230378 | [M-H]- | C46H82NO7P |
| 791.5488 | 279800 | SM(d18:1/20:1) | 44.375181 | [M+Cl]- | C43H85N2O6P |
| 792.5547 | 106000 | PE(18:0/22:5)_PE(20:1/20:4) | 0.231631 | [M-H]- | C45H80NO8P |
| 792.5547 | 106000 | PC(16:0/18:2) | 29.201638 | [M+Cl]- | C42H80NO8P |
| 792.5547 | 106000 | PC(O-16:0/22:5) | 46.13258 | [M-H]- | C46H84NO7P |
| 794.572 | 114100 | PE(18:0/22:4) | 1.84555 | [M-H]- | C45H82NO8P |
| 794.572 | 114100 | PC(16:0/18:1)_1_PC(16:0/18:1)_2_PC(16:0/18:1)_3 | 31.204213 | [M+Cl]- | C42H82NO8P |
| 794.572 | 114100 | PC(O-18:0/20:4) | 43.939059 | [M-H]- | C46H86NO7P |
| 834.5345 | 125600 | PS(40:6) | 6.51915 | [M-H]- | C46H78NO10P |
| 834.6282 | 113900 | PC(18:0/22:5) | 31.597127 | [M-H]- | C48H86NO8P |
| 835.5377 | 66070 | PI(16:0/18:1)_1_PI(34:1); PI(16:0/18:1)_2\|PI(18:0/16:1) | 4.184652 | [M-H]- | C43H81O13P |
| 835.6307 | 56550 | SM(d18:1/23:0) | 18.939683 | [M+Cl]- | C46H93N2O6P |
| 835.6307 | 56550 | TG(48:3) | 33.616926 | [M+Cl]- | C51H92O6 |
| 836.6317 | 62310 | PC(18:0/22:4) | 16.998234 | [M-H]- | C48H88NO8P |
| 836.6317 | 62310 | PE(40:1) | 44.874112 | [M+Cl]- | C45H88NO8P |
| 844.6495 | 59720 | PS(40:1) | 49.952705 | [M-H]- | C46H88NO10P |
| 844.6495 | 59720 | GlcCer(d42:2) | 6.664844 | [M+Cl]- | C48H91NO8 |
| 845.6539 | 32240 | SM(d18:2/24:1) | 27.225969 | [M+Cl]- | C47H91N2O6P |
| 846.6539 | 55120 | HexCer(d18:1/24:0) | 6.638418 | [M+Cl]- | C48H93NO8 |
| 848.6426 | 67880 | PC(O-22:1/20:4) and/or PC(P-22:0/20:4) | 13.27335 | [M-H]- | C50H92NO7P |
| 849.6437 | 31540 | SM(d18:1/24:0) | 21.746266 | [M+Cl]- | C47H95N2O6P |
| 849.6437 | 31540 | TG(49:3) | 36.181246 | [M+Cl]- | C52H94O6 |
| 860.6448 | 62180 | LacCer(d18:1/16:0) | 39.895657 | [M-H]- | C46H87NO13 |
| 860.6448 | 62180 | PC(O-40:3) | 16.566249 | [M+Cl]- | C48H92NO7P |
| 861.6487 | 36720 | TG(50:4) | 29.874717 | [M+Cl]- | C53H94O6 |
| 861.6487 | 36720 | SM(d19:1/24:1) | 15.640677 | [M+Cl]- | C48H95N2O6P |
| 862.6556 | 114100 | PC(O-22:0/18:2)_PC(O-40:2) | 10.905305 | [M+Cl]- | C48H94NO7P |
| 863.6594 | 53530 | TG(50:3) | 35.536334 | [M+Cl]- | C53H96O6 |
| 864.6604 | 44480 | PE(42:1) | 40.41336 | [M+Cl]- | C47H92NO8P |
| 864.6604 | 44480 | PC(O-40:1) | 1.668375 | [M+Cl]- | C48H96NO7P |
| 885.5558 | 52100 | PI(38:4); PI(16:0/22:4)\|PI(18:1/20:3)\|PI(18:0/20:4)_1_PI(18:0/20:4)_2 | 6.714947 | [M-H]- | C47H83O13P |
| 886.5605 | 30820 | SHexCer(d18:1/20:0(OH)) | 13.364659 | [M+Cl]- | C44H85NO12S |
| 888.6539 | 59940 | SHexCer(d18:1/24:1) | 33.643498 | [M-H]- | C48H91NO11S |
| 888.6539 | 59940 | PC(O-22:0/20:3) | 8.937798 | [M+Cl]- | C50H96NO7P |
| 888.6539 | 59940 | PE(44:3) | 32.007208 | [M+Cl]- | C49H92NO8P |

Table S4. LA-REIMS AmbiSampler result of MassPREP Peptide Mix Standard (positive ion mode)

| **Component name** | **Molecular weight (g/mol)** | **Expected exact mass in positive ion mode (m/z)** | **Measured mass (m/z)** | **Measured intensity of 5 combined scans** |
| --- | --- | --- | --- | --- |
| Allantoin (V0 marker) | 158.044 | 159.0513 | 159.0491 | 7.57e4 |
| RASG-1 | 1000.494 | 1001.501 | 1001.5336 | 9.44e5 |
| Angiotensin frag. 1–7 | 898.4661 | 899.4734 | 899.5032 | 1.48e6 |
| Bradykinin | 1059.561 | 1060.569 | 1060.6034 | 1.67e6 |
| Angiotensin II | 1045.535 | 1046.542 | 1046.5756 | 1.40e6 |
| Angiotensin I | 1295.678 | 1296.685 | 1296.7267 | 8.63e5 |
| Renin substrate | 1757.925 | 1758.933 | 1758.9741 | 1.59e5 |
| Enolase T35 | 1871.96 | 1872.968 | 1873.004 | 4.77e5 |
| Enolase T37 | 2827.281 | 2828.288 | n/A | n/A |
| Melittin | 2844.754 | 2845.761 | 2845.4519 | 5.08e3 |
